# Emotional Expressive Flexibility and Mental Health: A Systematic Review and Meta-Analysis

**DOI:** 10.3390/brainsci16101041

**Published:** 2026-09-29

**Authors:** Dirk W. Eilert, Philipp Mensah

**Affiliations:** 1 Institute of Psychology, University of Innsbruck, 6020 Innsbruck, Austria; 2 Eilert Institute for Emotion Science, 13585 Berlin, Germany; philipp.mensah@eilert-institute.org

**Keywords:** expressive flexibility, emotional expression, emotion processing, core functions of emotion processing (C-FEP) model, regulatory flexibility, transdiagnostic processes, psychopathology, psychological well-being, resilience, social functioning

## Abstract

**Highlights:**

**What are the main findings?**

- Evidence across 29 studies linked greater overall expressive flexibility with lower psychopathological symptom burden, greater psychological well-being, greater resilience and adaptive functioning, and better social functioning.
- The meta-analyses indicated small associations with fewer depressive and anxiety symptoms and preliminary evidence of an association with higher life satisfaction; effect sizes varied across populations and settings.

**What are the implications of the main findings?**

- Neither enhancing nor suppressing emotional expression is uniformly beneficial. The findings emphasize the mental-health relevance of expressive flexibility—the capacity to flexibly enhance and suppress emotional expression—with associations varying across outcomes and contexts.
- The findings identify emotion expression as a relevant domain for understanding mental health. C-FEP conceptualizes emotion expression as a core function of emotion processing and provides a framework for investigating differential functional profiles and potential transdiagnostic relevance.

**Abstract:**

**Background/Objectives**: The relationship between expressive flexibility—the capacity to enhance and suppress visible emotional responses according to situational demands—and mental health has not been systematically synthesized. This systematic review examined associations across psychopathology, psychological well-being, resilience and adaptive functioning, and social functioning. **Methods**: Following preregistration on the Open Science Framework (10.17605/OSF.IO/BA5HD), PsycINFO, PsycArticles, and PubMed were searched on 23 June 2026, supplemented by citation searching and expert consultation. English-language, peer-reviewed studies of participants aged at least 12 years assessing expressive flexibility and mental health were eligible. Two reviewers independently selected studies and assessed methodological quality using design-specific JBI critical appraisal tools. Narrative synthesis, random-effects meta-analyses, and a structured narrative appraisal of the evidence for each outcome were conducted. **Results**: Twenty-nine studies were included (cumulative analytic sample: *N* = 8357). Appraisals identified limitations in confounding control, handling of incomplete follow-up, and methodological reporting. Greater overall expressive flexibility was generally associated with lower psychopathological symptom burden, greater psychological well-being, greater resilience and adaptive functioning, and better social functioning. Component findings varied. Pooled associations were inverse for depressive symptoms (*k* = 9, *N* = 3622, *r* = −0.14, 95% CI [−0.26, −0.01]) and, exploratorily, anxiety (*k* = 7, *N* = 3134, *r* = −0.13, 95% CI [−0.23, −0.03]). The positive association with life satisfaction was preliminary (*k* = 4, *N* = 1910, *r* = 0.27, 95% CI [0.02, 0.49]). Leave-one-out analyses indicated uncertainty across all three syntheses. Heterogeneity was substantial to very high and all prediction intervals included zero. **Conclusions**: Findings suggest that expressive flexibility is an emotion-expression capacity associated with several dimensions of mental health, primarily in non-clinical samples. Its potential transdiagnostic relevance warrants further investigation, but limited robustness, substantial heterogeneity, and predominantly cross-sectional evidence preclude firm conclusions about generalizability and causal effects.

## 1. Introduction

### 1.1. Emotion Processing as a Transdiagnostic Foundation of Mental Health

Emotions are fundamental to human experience and adaptive functioning. They direct attention toward personally relevant events, influence perception and decision-making, prepare behavioral responses, and communicate information that coordinates social interaction [1,2,3]. These functions depend on emotion processing, understood here as the coordinated interplay of perceiving and understanding emotions in oneself, perceiving and understanding emotions in others, regulating emotional responses, and expressing emotions through verbal and nonverbal channels. Disturbances in these processes are evident across different forms of psychopathology, suggesting that impaired emotion processing constitutes a transdiagnostic liability that may contribute to both symptom overlap and heterogeneity across mental disorders [4,5].

To organize these interrelated processes within a unified framework, the *Core Functions of Emotion Processing (C-FEP) model* distinguishes four interdependent core functions according to their specific roles in emotion processing [6,7]. These comprise intrapersonal emotion perception (impathy), referring to the perception and understanding of one’s own emotional states [8]; interpersonal emotion perception (empathy), referring to the perception and understanding of others’ emotional states; emotion regulation, encompassing processes that influence the emergence, course, experience, and expression of emotion [9]; and emotion expression, encompassing the verbal and nonverbal behaviors through which emotional information becomes available to others. Emotion expression serves a communicative function through which emotional information influences social interaction [3]. Regulatory processes can modify emotional expression [9], while expressive behavior can in turn influence subjective emotional experience [10,11,12,13]. Within C-FEP, the distinction between emotion expression and emotion regulation therefore reflects their different functional contributions within an interacting system [6,7].

The specific contribution of C-FEP becomes clearer when considered in relation to other theoretical models of emotion. Gross’s process model distinguishes regulatory processes according to where they intervene in emotion generation [9], while Scherer’s Component Process Model describes how appraisal processes shape changes in interacting emotion components and drive their synchronization during emotional episodes [14]. C-FEP complements these accounts by bringing the perception of one’s own and others’ emotions, emotion regulation, and emotion expression into a common functional framework for examining their individual and joint relevance to mental health [6,7]. Its organizing focus is the contribution of these functions to emotion processing, allowing shared and differential patterns of association with mental health to be investigated across them. This framework guides the present review’s focus on emotion expression while situating it within the broader interplay among the core functions of emotion processing.

### 1.2. Emotion Expression Shapes Emotional Experience and Social Interaction

Emotion expression serves both intrapersonal and interpersonal functions. At the intrapersonal level, expressive behavior participates in a bidirectional body–emotion system: experimentally induced changes in facial activity, posture and movement, and the affective qualities of one’s own voice can feed back into subjective emotional experience as well as motivational and physiological responding, although the magnitude and robustness of these effects differ across modalities and experimental conditions [10,11,12,13]. Importantly, the intrapersonal effects of expressive behavior do not appear to require deliberate production [15], which is particularly relevant because emotional expressions may arise spontaneously and without conscious communicative intent [16].

At the interpersonal level, emotion expression constitutes the communicative interface through which aspects of an individual’s emotional state become available to others. It encompasses deliberate and spontaneous behavior across multiple channels, including facial movements, vocal cues, gaze, gestures, posture, touch, and language. Contemporary advances have moved beyond a narrow focus on six static facial configurations, describing a high-dimensional repertoire in which more than 20 emotional states are conveyed through multimodal and dynamic expressive patterns [16,17,18]. Beyond signaling an emotional state, expressive patterns can convey information about the eliciting circumstances, behavioral intentions, and the relationship between expresser and perceiver [16]. In turn, these signals influence observers’ emotional responses, situational inferences, and subsequent behavior, thereby contributing to the coordination of social interaction [3,16].

Because emotion expression shapes both internal experience and social interaction, its adaptive value depends not simply on how strongly or frequently emotions are expressed, but also on how expressive behavior is adjusted to situational demands and interpersonal goals.

### 1.3. Adaptive Emotion Expression Requires the Capacity to Enhance and Suppress

Adjustment of emotional expression may involve a discrepancy between what a person feels, what they display, and what a situation requires them to display. Emotional dissonance has been described as a discrepancy between experienced and displayed emotions [19,20]. The more specific term, emotion-rule dissonance, identifies a discrepancy between experienced emotions and those required by a social or occupational role [21]. Research conducted primarily in occupational settings has linked such discrepancies to emotional exhaustion and burnout [19,20,21,22,23], as well as higher job stress and lower intrinsic motivation [24]. Emotional dissonance has also been indirectly associated with poorer in-role performance through its relationship with burnout [19]. These findings highlight the importance of distinguishing emotional experience, expressive behavior, and external display requirements when examining mental health. Conformity with a display requirement does not necessarily indicate a corresponding change in subjective experience. However, these associations do not establish that every discrepancy is maladaptive or that greater expressive flexibility protects against its potential costs. Rather, they motivate examining how individuals adjust their expression and how this adjustment relates to both their emotional experience and the demands of the situation.

Against this background, expressive flexibility describes the capacity to alter the visibility of emotion in either direction as situational requirements change—enhancing expression in some situations and suppressing it in others [25]. Bonanno et al. [25] first operationalized this capacity in a within-subject laboratory paradigm in which participants viewed positive and negative images and were instructed either to respond naturally or to enhance or suppress their visible reactions. Enhancement and suppression abilities were calculated relative to each participant’s natural expressive baseline and then combined into an overall flexibility score. The instructions produced significant differences in observer-rated expression, whereas subjective emotional experience did not differ significantly across conditions [25]. This pattern supports distinguishing expressive modulation from changes in experienced emotion, although the nonsignificant difference in subjective experience does not establish their independence. Burton and Bonanno subsequently developed the Flexible Regulation of Emotional Expression (FREE) Scale, which assesses perceived enhancement and suppression ability across hypothetical social situations and differentiates the regulation of positively and negatively valenced emotional expressions [26]. Across both approaches, expressive flexibility concerns regulatory capacity to modulate emotional expression rather than the habitual frequency of expression or suppression [25,26]. Importantly, these measures assess whether a person can modify emotional expression once a regulatory direction has been specified; they do not by themselves establish whether the person accurately identifies which response is appropriate or adjusts it in response to feedback [27,28].

Although expressive flexibility is assessed under specific experimental or hypothetical conditions, initial evidence suggests that it captures more than momentary task performance. In a three-year follow-up of the original laboratory sample, Westphal et al. observed moderate stability for enhancement and suppression ability (both *r* = 0.44) and greater stability for the summed expressive-flexibility score (*r* = 0.62) [29]. At the same time, its association with adjustment remained context dependent: expressive flexibility was associated with better friend-rated adjustment primarily under high cumulative life stress, and the pattern varied according to whether expressive performance was assessed in the presence of an immediate threat prime [29]. Expressive flexibility may therefore be best understood as a relatively stable individual capacity whose behavioral manifestation and adaptive relevance vary across contexts. However, the stability estimates were based on only 32 undergraduates reassessed under the comparable neutral-prime condition and require replication in larger and more diverse samples.

Within C-FEP, emotion expression constitutes a core function through which emotional information is communicated and social interaction is shaped [3,6,7]. Expressive behavior does not simply mirror subjective experience: in the experimental paradigm described above, instructions altered observable expression without significant differences in reported emotional experience [25]. This finding supports examining expressive flexibility in terms of what individuals can change in their outward expression, without assuming a corresponding change in how they feel. Its assessment therefore concerns the ability to enhance and suppress visible emotional responses, whereas changes in subjective experience constitute a separate outcome to be examined [25,26]. C-FEP thus locates expressive flexibility within the expression function, while acknowledging its conceptualization as a form of emotion regulation in the foundational literature [9,25,26].

### 1.4. Expressive Flexibility as a Potential Transdiagnostic Factor: Review Objectives

Initial prospective research linked expressive flexibility to psychological adjustment following adversity, with prospective evidence from Bonanno et al. [25] and complementary cross-sectional evidence from a three-year follow-up of a partially overlapping cohort by Westphal et al. [29]. Subsequent studies broadened this focus by examining outcomes across negative and positive dimensions of mental health. Dual-factor models of mental health conceptualize psychopathology and subjective well-being as related but distinct dimensions rather than as opposite ends of a single continuum [30,31]. From this perspective, evaluating expressive flexibility as a factor relevant to mental health requires consideration of its associations with both psychopathological symptoms and positive well-being. Overall expressive flexibility or its constituent enhancement and suppression abilities have been associated with fewer depressive, anxiety, and stress symptoms, as well as with greater life satisfaction, resilience, and social functioning or support [26,32,33,34]. However, the strength of these associations varies across mental health outcomes and between overall expressive flexibility, enhancement, and suppression [28,29,32,33]. The breadth of outcomes examined suggests that expressive flexibility may be transdiagnostically relevant, but the available evidence has not yet been systematically synthesized.

Accordingly, the present review aimed to examine associations of overall expressive flexibility and its constituent enhancement and suppression abilities with psychopathology, psychological well-being, resilience and adaptive functioning, and social functioning across adolescent and adult clinical and non-clinical populations. Based on existing theoretical and empirical work, we hypothesized that greater expressive flexibility would be associated with lower levels of psychopathology and psychological distress and with higher levels of psychological well-being, resilience, and adaptive functioning. We further hypothesized that the relationship between expressive flexibility and mental health would extend across different populations and outcome domains, consistent with its proposed role as a transdiagnostic factor.

## 2. Materials and Methods

### 2.1. Protocol, Registration, and Reporting Standards

This systematic review was reported in accordance with the Preferred Reporting Items for Systematic Reviews and Meta-Analyses (PRISMA) 2020 statement [35]. The review protocol was prospectively registered on the Open Science Framework on 23 June 2026 (OSF; https://osf.io/ba5hd; doi:10.17605/OSF.IO/BA5HD; Center for Open Science, Washington, DC, USA). Registration was completed before the systematic database searches, study screening, or systematic inspection of study results for this review began. The registration specified the review questions, eligibility criteria, information sources and search strategy, study selection and data extraction procedures, methodological quality assessment, and planned approach to evidence synthesis. Deviations from the registered protocol are identified and justified in the relevant Methods subsections.

### 2.2. Information Sources and Search Strategy

A systematic literature search was conducted on 23 June 2026 in PsycINFO and PsycArticles via APA PsycNet (American Psychological Association, Washington, DC, USA) and in PubMed (National Library of Medicine, Bethesda, MD, USA), covering all records indexed from database inception to the search date. These databases were selected to provide complementary coverage of psychological, clinical, and biomedical research relevant to expressive flexibility and mental health. On 24 June 2026, citation chaining was conducted as a supplementary search method. On the same date, George A. Bonanno, who pioneered the empirical study of expressive flexibility by leading its first operationalization, was contacted to identify potentially overlooked studies. In accordance with the preregistered protocol, no formal search of the grey literature was conducted.

The review question and eligibility criteria were structured according to the Population–Exposure–Outcome (PEO) framework. Because the eligible populations and outcome domains were intentionally broad, including these components in the electronic queries risked excluding relevant studies. The database searches therefore operationalized the Exposure component using the two established labels “expressive flexibility” and “flexible regulation of emotional expression.” Both terms were searched in the title, abstract, and keyword fields in PsycINFO and PsycArticles and in the title and abstract fields in PubMed. Searches in PsycINFO and PsycArticles were limited to peer-reviewed publications; no other filters or limits were applied, and the PubMed search was conducted without filters. The complete database-specific search strategies are presented in Table 1.

The search strategy was checked against several previously known publications. Known-item testing confirmed retrieval of studies spanning experimental and self-report assessments and different mental-health outcomes, including Westphal et al. [29], Gupta and Bonanno [36], Burton and Bonanno [26], Rodin et al. [37], and Chen et al. [32]. The foundational study by Bonanno et al. [25] was not retrieved by the database queries. It introduced the experimental operationalization of expressive flexibility but described this capacity in its title and abstract without either exact search phrase; the term “expressive flexibility” appeared in the full text, which was not searched by our queries. The study was identified through the supplementary citation search and included in the review.

### 2.3. Eligibility Criteria

Study eligibility was determined using the preregistered Population–Exposure–Outcome framework summarized in Table 2. Studies were eligible if they examined adolescents aged 12 years or older or adults from clinical or non-clinical populations, assessed emotional expressive flexibility as a distinct construct, and investigated its association with at least one relevant mental-health or adaptive-functioning outcome.

The minimum age of 12 years was a pragmatic, preregistered boundary defining the population scope of this review, rather than a general definition of the onset of adolescence or a specific developmental threshold. Studies including participants younger than 12 years were eligible only when findings for participants aged 12 years or older were reported separately; otherwise, they were excluded.

The review considered empirical articles published in English in peer-reviewed scientific journals. Eligibility was not restricted by publication year, country, or study design. Reviews and meta-analyses, theoretical articles, editorials, commentaries, conference abstracts, dissertations and theses, and book chapters were excluded. Studies were also excluded if they did not assess expressive flexibility as a distinct construct or did not examine its association with an eligible outcome.

### 2.4. Study Selection and Data Extraction

All identified records were imported into Covidence systematic review software (Veritas Health Innovation, Melbourne, Australia; used June–September 2026) for duplicate removal and study selection. Although the preregistration specified DistillerSR (DistillerSR Inc., Ottawa, ON, Canada) for these stages, Covidence was used instead for organizational reasons; this software substitution did not alter the predefined eligibility criteria or screening procedure. After duplicates had been removed, two reviewers (D.W.E. and P.M.) independently screened all titles and abstracts and subsequently assessed the full texts of potentially eligible records. Records were retained for full-text assessment whenever eligibility could not be determined with sufficient certainty from the title and abstract. Disagreements at either screening stage were discussed until consensus was reached. No automation tools were used to make study-eligibility decisions. Inter-reviewer agreement was quantified separately for title and abstract screening and full-text assessment using observed percentage agreement and Cohen’s κ. During peer review, the application of the eligibility criteria was re-examined so that eligibility depended on the associations reported rather than on a publication’s primary aim or on the predictor–outcome designation of cross-sectional correlations. As a result, two studies initially excluded at full-text assessment were included [38,39].

Data were extracted directly into a standardized workbook using Microsoft Excel for Mac, version 16.112 (Microsoft Corporation, Redmond, WA, USA). This represents a deviation from the preregistered procedure, which specified the use of a DistillerSR extraction form; the change was made for organizational reasons and did not affect the predefined data items. One reviewer (D.W.E.) extracted the data, and a second reviewer (P.M.) independently verified all entries against the original publications. Extracted study characteristics comprised authorship and publication year, country, study design and aim, population characteristics, sample size, age and gender distribution, and the measure used to assess expressive flexibility. Outcome-level extraction included the mental-health outcome and corresponding measure, the expressive-flexibility variable examined, statistical analysis, covariates or adjustments, reported effect estimates, and the direction and interpretation of the association. During revision, we additionally extracted study funding and author-reported conflicts of interest from the included publications. Explicit statements of no funding or no conflicts were distinguished from absent reporting, which was recorded as “Not reported.” These disclosures are documented in the accompanying study-characteristics dataset.

Population classification was based on recruitment setting and clinical eligibility criteria. Samples recruited as patients receiving clinical care or selected on the basis of a diagnosis or a specified clinically relevant symptom threshold were classified as clinical. Community, educational, or occupational samples without such selection criteria were classified as non-clinical; the presence of symptoms alone did not determine classification. Studies combining clinically defined groups with comparison participants who did not meet the corresponding clinical criterion were classified as mixed. These categories describe sample composition and do not imply that all participants classified as clinical had a confirmed psychiatric diagnosis.

For studies reporting multiple analyses, eligibility for outcome-level extraction was determined by whether an analysis examined an association between overall expressive flexibility or its constituent enhancement and suppression abilities and an outcome meeting the criteria specified in Section 2.3. Depending on the analyses reported, extracted findings included bivariate and partial correlations, regression coefficients, group-comparison and longitudinal models, and moderation or mediation effects. Study eligibility was determined by the population, constructs, and associations examined, irrespective of the publication’s primary aim. Within eligible studies, analyses addressing the association between expressive flexibility and an eligible outcome were considered for extraction, including those reported as part of measure validation. Cross-sectional correlations were treated as nondirectional associations, irrespective of which variable the original authors described as the predictor or outcome. For experimental studies, outcome-level extraction focused on effects of expressive regulation on eligible mental-health or social-functioning outcomes. Experiments manipulating social experiences and assessing subsequent expressive behavior were considered contextual evidence concerning potential influences on expressive functioning. Analyses addressing only psychometric properties or antecedents outside the eligible outcome domains were not included in the outcome-level synthesis; selected contextual findings were recorded separately as interpretive notes. The outcome-level dataset supported the narrative synthesis, whereas eligibility for the meta-analyses was determined separately using the criteria described in Section 2.7.

Ambiguities or discrepancies were checked against the original publication and resolved through discussion. No missing values were imputed; information that could not be established from the original publication was recorded as not reported. Study authors were not contacted to obtain or confirm missing outcome data.

The complete study- and outcome-level extraction dataset is publicly available through the accompanying OSF project.

### 2.5. Methodological Quality Assessment

The methodological quality of the included evidence was assessed independently by two reviewers (D.W.E. and P.M.) using the JBI critical appraisal tools for analytical cross-sectional studies [40], cohort studies [41], and randomized controlled trials [42]. During the revision process, these design-specific instruments replaced the preregistered adapted Newcastle–Ottawa Scale to better address the methodological requirements of the different contributions included in the review. The appraisal tool was selected according to the design of the analysis contributing to the synthesis rather than solely according to the overall design of the original publication.

Studies contributing both cross-sectional and longitudinal findings were appraised separately for each design. Observational associations between expressive flexibility and mental-health outcomes embedded within laboratory experiments were assessed using the analytical cross-sectional tool when the reviewed association did not estimate an effect of randomized assignment. The randomized peer-acceptance experiment [43] was assessed using the randomized controlled trial tool. Review-specific applicability rules, including the treatment of continuous exposures and outcomes in cohort studies, are documented in the Supplementary Material.

Each item was rated as Yes, No, Unclear, or Not applicable, with supporting rationales and source locations. Judgments were differentiated by outcome or analysis where necessary; outcomes were grouped only when their judgments were identical. Confounding-control judgments for adjusted analyses were not extended to separately reported unadjusted correlations. No summed scores, numerical thresholds, or overall quality categories were calculated. Findings were summarized by methodological domain and design, distinguishing identified limitations from uncertainty due to insufficient reporting.

Before completing the JBI appraisal, both reviewers independently assessed a pilot set of five included studies and discussed discrepancies to establish a shared understanding of the application rules. The remaining studies were then assessed independently, and disagreements were resolved through discussion until consensus was reached. The Supplementary Material provides the final consensus judgments, the original checklist questions, review-specific application rules, and supporting rationales with source locations for each item. Separate appraisal sheets document the cross-sectional and longitudinal contributions of studies that informed both types of synthesis.

### 2.6. Data Synthesis and Outcome-Level Evidence Appraisal

Given the heterogeneity of the included populations, study designs, measures of expressive flexibility, and outcomes, the evidence was first synthesized narratively. Findings were organized into the preregistered domains of psychopathology, psychological well-being, and resilience and adaptive functioning. Within each domain, results were grouped by outcome construct and, where applicable, by overall expressive flexibility, enhancement ability, and suppression ability.

Social functioning was added as an additional narrative synthesis domain after data extraction because several eligible studies examined distinct interpersonal outcomes, including social support, peer acceptance, and friendship quality. This organizational change did not modify the preregistered eligibility criteria or result in the inclusion of additional studies.

During revision, we conducted a structured narrative appraisal of the evidence for each outcome, integrating the outcome- and analysis-specific JBI judgments with the narrative and quantitative syntheses. The appraisal considered risk of bias, consistency, precision and robustness, applicability, and potential publication and reporting bias. Concurrent associations, prospective findings, and experimental contrasts were distinguished, as were findings concerning overall expressive flexibility and its component abilities.

For pooled outcomes, the appraisal considered confidence intervals, heterogeneity, prediction intervals, and sensitivity analyses. For outcomes synthesized narratively, it considered differences in measures, populations, and designs, together with the extent of independent replication, without assigning an aggregate precision judgment where no comparable quantitative synthesis was available. Both reviewers independently conducted the appraisal and resolved disagreements through discussion. Conclusions were expressed as outcome-specific statements about the supported associations and their limitations, without assigning categorical certainty ratings. This structured narrative appraisal does not constitute a formal GRADE assessment [44]. The assessment rules, supporting evidence, and conclusions are provided in the supplementary document “Outcome-level appraisal of the evidence.”

### 2.7. Meta-Analytic Procedures

Three separate meta-analyses were conducted to examine the associations of overall expressive flexibility with depressive symptoms, anxiety, and life satisfaction. Depressive symptoms and life satisfaction constituted the primary quantitative syntheses, whereas the analysis of anxiety was considered exploratory. The preregistration specified that a meta-analysis would be conducted where sufficient methodological and statistical homogeneity was identified across studies. The two primary outcomes were selected to represent the complementary dimensions proposed in dual-factor models of mental health [30,31]. Depressive symptoms were selected as the most frequently examined psychopathological outcome, whereas life satisfaction was selected as the most frequently examined and consistently operationalized indicator of subjective well-being. Anxiety was analyzed exploratorily because it was the only additional psychopathological outcome for which a sufficient number of comparable studies was available, thereby enabling an exploratory assessment of the broader transdiagnostic relevance of expressive flexibility.

These outcome-specific decisions were not specified in the preregistration and were made after data extraction. No adjustment for multiplicity was applied across the three syntheses, which partly draw on the same primary studies. Results were accordingly interpreted in an estimation-oriented manner, with emphasis on confidence and prediction intervals rather than on binary significance decisions, and the exploratory analysis was not treated as confirmatory. Other outcomes were not quantitatively synthesized because too few sufficiently comparable studies were available.

Studies were eligible for the meta-analyses if they assessed overall expressive flexibility and reported a direct association with the respective outcome as a single, uniquely identifiable correlation coefficient. For the primary models, concurrent associations were prioritized over prospective associations when both were available from the same sample, to improve comparability in the temporal relationship between expressive flexibility and the outcome. This selection rule was specified during the revision process. Each sample contributed only one effect estimate to each model.

The depression analysis included outcomes referring to depression or depressive symptoms assessed using established self-report symptom measures; for one included study [26], the depressive-symptom instrument was not specified in the article, and this study was retained in the primary model, with its influence quantified in the leave-one-out analyses. All coefficients included in the primary depression model reflected concurrent associations; for Southward and Cheavens [28], the baseline correlation was selected, and the alternative one-month prospective correlation was examined in a sensitivity analysis.

The anxiety analysis included concurrent associations with anxiety symptoms, state anxiety, and trait anxiety, each assessed using established self-report measures. Social anxiety was not pooled because it represented a more specific social–interpersonal construct and the available result did not provide a single uniquely identifiable correlation coefficient.

The life-satisfaction analysis included concurrent associations assessed using the Satisfaction With Life Scale, which was used consistently across all included studies.

Associations involving the separate enhancement or suppression ability components, mixed outcome constructs, regression coefficients, moderation effects, or other adjusted model parameters were excluded from the main model of each synthesis. The expressive-flexibility measures used in the individual studies are summarized with other study characteristics in Section 3.2. Outcome measures are described within their respective domains in Section 3.4.1, Section 3.4.2, Section 3.4.3 and Section 3.4.4, whereas the complete study-level extraction, including detailed outcome measures and statistical results, is available in the OSF repository associated with this review.

The main model of each synthesis was restricted to unadjusted Pearson correlations to ensure that the pooled effect sizes were statistically comparable. When a study reported eligible effects for multiple independent subsamples, the correlations were transformed to Fisher’s *z*, combined using inverse-variance weighting, and entered into the meta-analysis as a single study-level effect. This procedure was applied to the independent subsamples reported by Ang et al. [45], and to the India and United States subsamples reported by Strickland and Skolnick [46]. Fisher’s *z*-transformed correlations and their corresponding sampling variances were calculated using the *metafor* package (version 5.0-1) in R (version 4.5.3; R Core Team, Vienna, Austria) [47,48].

Separate random-effects models were estimated for each outcome using restricted maximum likelihood estimation (REML) and the Hartung–Knapp adjustment for statistical inference. Pooled estimates and confidence intervals were subsequently back-transformed to Pearson’s *r* for interpretation. Statistical heterogeneity was assessed using Cochran’s *Q*, *I*^2^, and *τ*^2^, and 95% prediction intervals were calculated to estimate the range of effects expected across comparable populations and settings. Prediction intervals based on fewer than five studies are reported for completeness but are highly imprecise and were interpreted with corresponding caution. Given the small number of studies available for each synthesis, no subgroup analyses or meta-regressions were conducted to explore potential sources of heterogeneity.

Forest plots were generated for all three syntheses to display the individual study estimates with their 95% confidence intervals and the corresponding pooled effects.

Robustness was examined using leave-one-out analyses and influence diagnostics for each meta-analysis. For depressive symptoms, three additional sensitivity models were estimated: one incorporating both Pearson and Spearman correlations, one additionally incorporating partial and partial-rank correlations, and one replacing the baseline correlation from Southward and Cheavens [28] with the one-month prospective correlation from the same sample. In the first two of these models, Spearman and partial coefficients were treated as approximate Pearson correlations and their sampling variances were approximated by the ordinary formula for product-moment correlations; these models therefore serve as robustness checks rather than as exact equivalents of the primary model. For anxiety, one sensitivity analysis replaced the baseline correlation from Southward and Cheavens [28] with the one-month prospective correlation from the same sample; a second restricted the primary model to state- or symptom-based anxiety outcomes, excluding trait-anxiety measures. Separate effect-type sensitivity models were not conducted for life satisfaction because all eligible effects were concurrent, unadjusted Pearson correlations and only four independent study units were available.

Potential reporting bias and small-study effects were assessed for each of the three syntheses. Funnel plots were inspected descriptively for the depression and anxiety analyses (*k* = 9 and *k* = 7, respectively). Egger-type regression tests were not conducted because no analysis reached the minimum of ten independent study effects that we had specified before conducting the analyses. A funnel plot was not produced for the life-satisfaction analysis because only four independent study units were included. Given the small numbers of studies and the substantial between-study heterogeneity, these assessments were considered exploratory, and the absence of a formal test was not interpreted as evidence for or against reporting bias.

### 2.8. Use of Generative Artificial Intelligence

Generative artificial intelligence (GPT-5.6 Sol, OpenAI, San Francisco, CA, USA) was used in a limited, supportive capacity during manuscript preparation, including language refinement, improvements to readability and stylistic consistency, and assessment of conceptual coherence. All theoretical concepts, hypotheses, methodological decisions, evidence syntheses, interpretations, and conclusions were critically evaluated and approved by the human authors, who retain full responsibility for the content of this review.

## 3. Results

### 3.1. Study Selection

**Figure 1:**
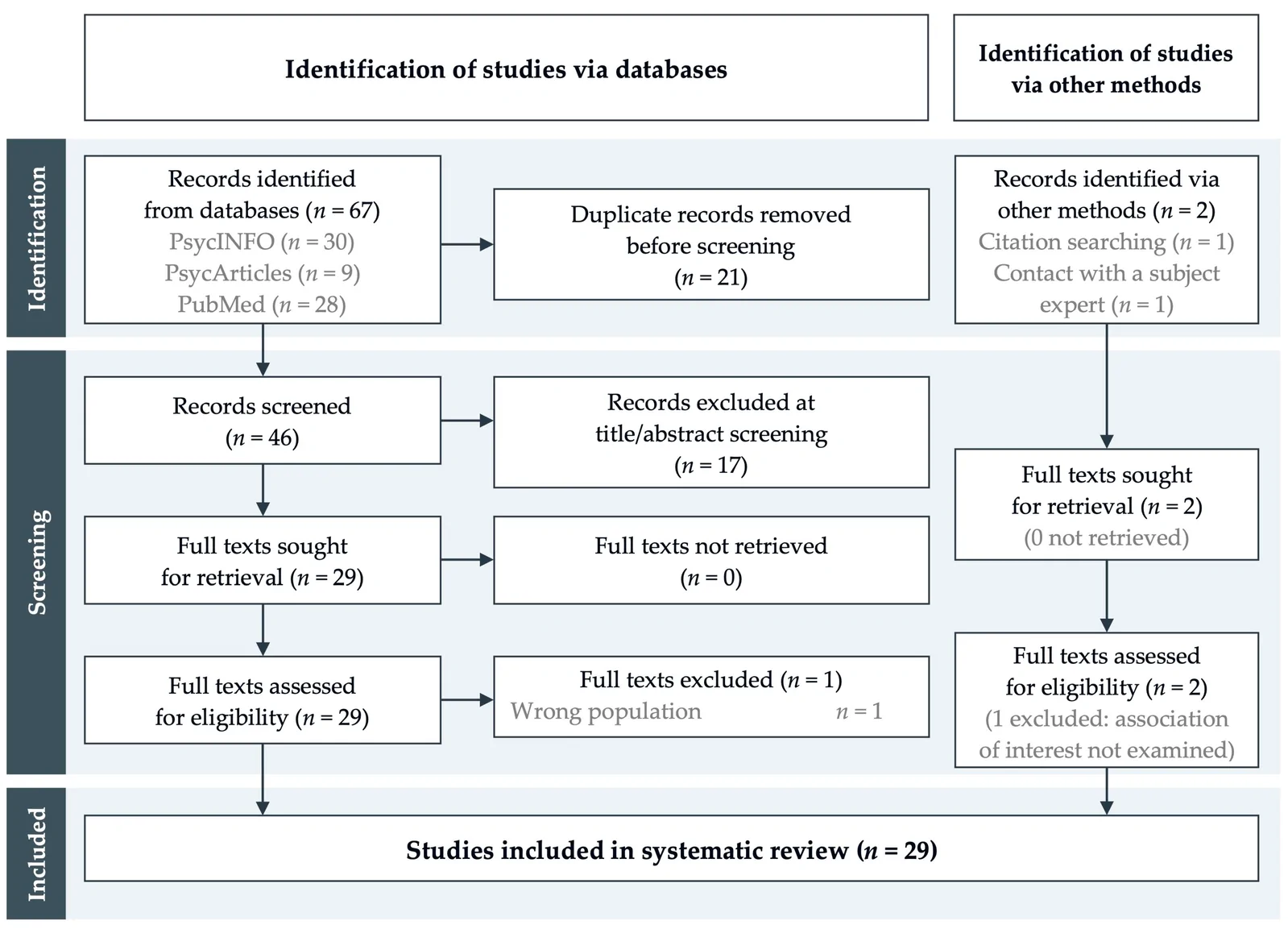
PRISMA flow diagram of the study selection process.

The database searches identified 67 records across PsycINFO (*n* = 30), PsycArticles (*n* = 9), and PubMed (*n* = 28). After 21 duplicates had been removed, 46 records underwent title and abstract screening, of which 17 were excluded. Full texts were retrieved and assessed for the remaining 29 records. One study was excluded because it examined an ineligible population, resulting in 28 studies identified through database searching. The supplementary searches yielded two further reports. One study identified through citation searching met the eligibility criteria. Expert consultation identified one additional study on expressive flexibility; because this study did not examine an association between expressive flexibility and a mental health outcome, it fell outside the scope of the present review. Overall, 29 studies were included in the systematic review (Figure 1).

Observed inter-reviewer agreement was high at both stages of study selection. During title and abstract screening, the reviewers independently agreed on 45 of 47 decisions (95.7%; Cohen’s *κ* = 0.91). This calculation comprised the 46 records identified through database searching and the additional record identified through citation searching; the study identified through expert consultation proceeded directly to full-text assessment and was therefore not included at this stage. The title and abstract decisions comprised 30 joint retention decisions, 15 joint exclusion decisions, and two disagreements. During full-text assessment, the reviewers agreed on 28 of 31 decisions (90.3%; Cohen’s *κ* = 0.52). The 31 full texts comprised the 29 reports identified through database searching and the two additional studies identified through citation searching and expert consultation. The independent assessments resulted in 26 joint inclusion decisions, two joint exclusion decisions, and three disagreements. The comparatively lower *κ* for full-text assessment reflects the predominance of inclusion decisions, which resulted in an expected chance agreement of 79.9%. All disagreements at both stages were resolved through discussion.

Table 3 lists the studies excluded after full-text assessment and their respective exclusion reasons.

### 3.2. Characteristics of the Included Studies

The characteristics of the included studies are summarized in Table 4. The 29 studies were published between 2004 and 2026 and encompassed samples from nine countries. Twelve studies were conducted exclusively in the United States, seven in China, and four in Italy. The remaining six studies were conducted in Australia, France, Germany, Israel, or Romania, or included participants from both India and the United States. With respect to the analyses included in this review, 22 studies contributed exclusively cross-sectional analyses, five contributed both cross-sectional and longitudinal analyses, one contributed exclusively longitudinal analyses, and one contributed experimental analyses. Most studies examined non-clinical populations (*n* = 24); three focused on clinical populations, and two included both clinical and non-clinical participants.

Study-level analytic sample sizes ranged from *n* = 50 to *n* = 1122 and yielded a cumulative sample size of 8357 across the 29 study entries. Because two studies drew partly on the same cohort, this cumulative figure does not represent the number of unique individuals. Mean age was reported for 28 studies and ranged at the study level from *M* = 13.27 years (*SD* = 0.38) to *M* = 80.26 years (*SD* = 6.39), reflecting samples from adolescence through older adulthood. Study-specific means, standard deviations, and reported age ranges are presented in Table 4. Sex or gender information corresponding to the respective analytic samples was available for 27 studies, covering 8132 participants. Of these, 4456 participants (54.8%) were reported as female, 3663 (45.0%) as male, and 12 (0.1%) under another gender category. Two studies did not report the sex or gender distribution for the relevant analytic sample.

Expressive flexibility was assessed using two methodological approaches. Nine studies employed the performance-based Expressive Regulation Paradigm (ERP) [25], whereas 20 used self-report measures. Among the latter, eight administered the original Flexible Regulation of Emotional Expression (FREE) Scale [26], ten used language-specific versions in Chinese, German, Italian, or French, and two employed age-adapted instruments developed for adolescents. Thus, self-report assessments of perceived expressive ability predominated over behavioral assessments of demonstrated expressive ability.

### 3.3. Methodological Quality

Design-specific JBI critical appraisal yielded 34 assessments across the 29 included studies: 27 for analytical cross-sectional contributions, six for cohort contributions, and one for a randomized experiment. Five studies contributed both cross-sectional and longitudinal analyses and therefore received separate appraisals. The final item-level judgments are presented in Tables S1–S3 of the supplementary document “JBI critical appraisal of expressive flexibility studies,” with supporting rationales and source locations in Section S5 of that document. No summed scores or overall quality categories were assigned.

For cross-sectional contributions, most appraisals supported the adequacy of outcome measurement and the statistical methods used. Limitations concerned incomplete reporting of eligibility criteria or study settings, inconsistent control of confounding, and uncertainty about particular instrument versions or scoring procedures. Confounding judgments differed between outcomes or analyses within some studies; affirmative judgments for adjusted analyses did not extend to separately reported unadjusted correlations. In three cross-sectional appraisals, unaddressed dependence among participants recruited within schools or work teams raised uncertainty about statistical inference [52,62,64].

For cohort contributions, recurring limitations concerned follow-up completeness and the handling of missing follow-up data. For follow-up completeness and the description and exploration of losses, one appraisal received a Yes judgment, one received No, and four received Unclear. None of the six contributions received an affirmative judgment for strategies addressing incomplete follow-up. These findings reflect differing levels of attrition and reporting completeness and do not establish that all six contributions were equally affected by attrition bias. Although all six appraisals identified confounding-control strategies, adjustment for the baseline value of the same outcome was not universal. Temporal ordering also required qualification: in one study, expressive flexibility and distress were both measured at follow-up, so the analysis did not establish prospective prediction of distress by earlier expressive flexibility [61].

For the randomized peer-acceptance experiment [43], outcome measurement and statistical analysis received affirmative judgments. However, insufficient reporting of the randomization procedure, allocation concealment, baseline comparability, blinding, and the handling of excluded participants resulted in several Unclear judgments. These judgments reflect unresolved information gaps rather than demonstrated failures of the procedures.

### 3.4. Expressive Flexibility Across Mental Health Outcome Domains

The included studies examined outcomes across four domains:

1. psychopathology,
2. psychological well-being,
3. resilience and adaptive functioning, and
4. social functioning.

Several studies assessed outcomes from more than one domain and are therefore represented in multiple subsections.

Table 5 provides a condensed overview of the outcome clusters and principal findings across domains, whereas the complete study-level extraction, including the outcome measures and statistical estimates, is available in the OSF repository associated with this review. The quantitative syntheses of depressive symptoms, life satisfaction, and anxiety are reported separately in Section 3.5.

#### 3.4.1. Expressive Flexibility and Psychopathology

The 22 studies assessing psychopathological outcomes covered five clusters: depressive symptoms and major depressive disorder; anxiety-related symptoms; stress-related symptoms; trauma-related outcomes; and general psychological distress and other psychopathology-related outcomes.

*Depressive symptoms* were examined in 15 studies. Seven studies used the depression subscale of the 21-item Depression Anxiety Stress Scales (DASS-21) [33,38,45,53,55,56,65], three used the Beck Depression Inventory-II (BDI-II) [32,37,57], one used a Chinese version of the Beck Depression Inventory (BDI) [34], and three used the Center for Epidemiologic Studies Depression Scale (CES-D) [28,39,64]; the instrument was not specified in one study [26]. Across seven cross-sectional studies, higher overall expressive flexibility was associated with fewer depressive symptoms [26,32,33,34,53,57,64].

Component-level findings showed that enhancement and suppression contributed differently across studies. In four studies, greater suppression ability was associated with fewer depressive symptoms, whereas enhancement ability showed no clear or independently robust association [26,32,33,57]. In Zhang et al. [64] and Rodin et al. [37], greater enhancement ability, but not suppression ability, was associated with fewer depressive symptoms; veterans with major depressive disorder also demonstrated lower enhancement ability than those without the disorder [37]. Lenzo et al. [53] found inverse bivariate associations for both abilities, but only enhancement ability remained independently associated after adjustment. Zhu and Bonanno [39] examined changes in expressive abilities between two task phases. Greater improvement in enhancement ability was associated with fewer depressive symptoms after adjustment for changes in affective enhancement and suppression abilities and feedback group, whereas the association was not significant in the initial model containing only expressive ability changes. Changes in suppression ability were not significantly associated with depressive symptoms, and feedback group did not significantly moderate either association. Depression was assessed before the tasks; these findings therefore concern associations with symptom levels rather than reductions in symptoms over time. Consistent with expressive flexibility as a combined capacity, Gonzalez-Escamilla et al. [33] additionally found an interaction between enhancement and suppression, indicating that the relationship of each ability with depressive symptoms depended on the level of the other.

Additional findings qualified the pattern of associations with depressive symptoms. In Shangguan et al. [34], the inverse association between overall expressive flexibility and depressive symptoms was indirect, mediated by perceived social support. Zhao and Wang [65] reported inverse associations between depressive symptoms and both overall expressive flexibility and suppression ability, neither of which met the conventional threshold for statistical significance. Clear associations were not observed in Ang and Tsai [45], Maccallum et al. [55], or Nardelli et al. [56]. Southward and Cheavens [28] found no significant association between overall expressive flexibility and depressive symptoms either at baseline or at one-month follow-up. Dwyer et al. [38] likewise reported no significant association between overall expressive flexibility and depressive symptoms in adults selected for harmful alcohol use. Correlations with depression, anxiety, and stress were reported collectively as nonsignificant after Bonferroni correction (all adjusted *p*s > 0.760), without individual coefficients or exact *p*-values.

Together, these findings generally suggest an inverse cross-sectional association between overall expressive flexibility and depressive symptoms, although this association was not consistently observed across studies. Component-specific, indirect, and prospective findings further varied across populations and analytic models.

*Anxiety-related outcomes* were examined in 12 studies. Six studies used the anxiety subscale of the DASS-21 [33,38,53,55,56,65], four used the trait scale of the State–Trait Anxiety Inventory (STAI-T) [46,55,57,60], one used the Beck Anxiety Inventory (BAI) [32], and one used the state scale of the STAI (STAI-S) [28]; Maccallum et al. [55] administered both the DASS-21 and the STAI-T. Social anxiety was examined in one study [62]. Four studies reported cross-sectional inverse associations between overall expressive flexibility and anxiety symptoms [32,46,53,60], although the association in Strickland and Skolnick [46] was no longer evident after accounting for collectivist values. Gonzalez-Escamilla et al. [33], Pitur and Miu [57], and Zhao and Wang [65] reported negative coefficients for the associations between overall expressive flexibility and anxiety symptoms, but these did not meet the conventional threshold for statistical significance. Likewise, Dwyer et al. [38] reported no significant association between overall expressive flexibility and anxiety symptoms after correction for multiple testing.

Component-specific findings varied: greater suppression ability was associated with fewer anxiety symptoms in three studies [32,53,65], whereas the inverse association between suppression ability and trait anxiety reported by Pitur and Miu [57] did not meet the conventional threshold for statistical significance. Greater enhancement ability was associated with lower trait anxiety in Maccallum et al. [55], and Shangguan et al. [60] found inverse associations for both abilities. Nardelli et al. [56] found no clear associations after correction for multiple testing.

In Wang et al. [62], overall expressive flexibility was neither concurrently associated with nor prospectively predictive of social anxiety. Southward and Cheavens [28] found no association between overall expressive flexibility and anxiety symptoms either at baseline or at one-month follow-up. For the combined anxiety and depression outcome, however, expressive flexibility predicted subsequent symptom reductions conditionally among participants with lower context sensitivity and was indirectly associated with symptom reductions through greater feedback sensitivity.

Taken together, the anxiety-related findings point predominantly toward inverse cross-sectional associations, whereas prospective relationships were less consistent and appeared to depend on related flexibility processes and the specific anxiety outcome examined.

*Stress symptoms* were examined in six studies, all of which used the stress subscale of the DASS-21 [33,38,53,55,56,65]. Higher overall expressive flexibility was associated with fewer stress symptoms in three studies [33,53,65], whereas Nardelli et al. [56] and Dwyer et al. [38] found no association after correction for multiple testing.

Component-level findings indicated a comparatively consistent role for suppression ability. Greater suppression ability was associated with fewer stress symptoms in three studies [33,56,65]. Lenzo et al. [53] found inverse bivariate associations for both enhancement and suppression abilities, but only suppression ability remained independently associated after adjustment. Maccallum et al. [55] found no clear association for either ability. Gonzalez-Escamilla et al. [33] additionally found an interaction between enhancement and suppression, indicating that their associations with stress symptoms depended on their combination.

Across the three symptom domains, Zhao and Wang [65] regrouped the four FREE subscales by emotional valence, distinguishing expressions of pleasant emotions (positive valence) from expressions of unpleasant emotions (negative valence). In analyses adjusted for age, gender, and emotion regulation self-efficacy, the two valence-specific expressive ability factors were examined simultaneously. Greater ability to enhance and suppress expressions of pleasant emotions was associated with fewer depressive, anxiety, and stress symptoms, whereas the corresponding ability for expressions of unpleasant emotions was associated with more symptoms across all three domains. The valence-specific expressive flexibility scores showed the same directional pattern, but less consistently. Greater flexibility for expressions of pleasant emotions showed negative associations with depressive and stress symptoms, neither of which met the conventional threshold for statistical significance (both reported as *p* < 0.10). Greater flexibility for expressions of unpleasant emotions was associated with more depressive symptoms (*p* < 0.05), whereas its positive association with anxiety symptoms did not meet this threshold (reported as *p* < 0.10). The remaining associations were not statistically significant.

*Trauma-related outcomes* were examined in two studies. Levy-Gigi et al. [54] assessed posttraumatic stress disorder (PTSD) symptoms using the PTSD Checklist for DSM-5 (PCL-5) among firefighters, whereas Rodin et al. [37] assessed PTSD diagnosis and symptom severity using the Posttraumatic Diagnostic Scale (PDS) among combat-exposed veterans. Rodin et al. [37] examined enhancement and suppression abilities separately but did not assess overall expressive flexibility. Greater enhancement ability was associated with fewer PTSD symptoms in both studies, and veterans with PTSD demonstrated lower enhancement ability than those without PTSD [37,54]. Suppression ability showed no clear association with PTSD-related outcomes in either study [37,54]. In Levy-Gigi et al. [54], higher overall expressive flexibility was associated with fewer PTSD symptoms and attenuated the association between repeated traumatic exposure and PTSD symptoms: greater exposure was associated with more severe symptoms at lower, but not higher, levels of expressive flexibility.

The remaining cluster encompassed *general psychological distress*, *complicated grief*, *borderline symptoms*, and *drug-related problems*. General psychological distress, operationalized as a broad composite of psychopathological symptoms, was examined in two studies. Wang et al. [61] assessed distress using the Brief Symptom Inventory-18 (BSI-18) among sexual minority men, whereas Nardelli et al. [56] used the DASS-21 total score. In Wang et al. [61], expressive flexibility was assessed only at the one-year follow-up (T2). Higher overall expressive flexibility was associated with lower concurrent distress, including after adjustment for baseline distress. Expressive flexibility also moderated the associations of both concurrent and baseline perceived sexual orientation-related discrimination with distress at T2. The latter model examined a prospective discrimination–distress association with expressive flexibility measured concurrently with the outcome.

At the component level, greater suppression ability was associated with lower concurrent distress and attenuated the concurrent discrimination–distress association, whereas the inverse association between enhancement ability and distress did not meet the conventional threshold for statistical significance (*p* = 0.08). In contrast, Nardelli et al. [56] found no clear associations between psychological distress and overall expressive flexibility, enhancement ability, or suppression ability after correction for multiple testing.

Three additional psychopathology-related outcomes were each examined in a single study. Gupta and Bonanno [36] found that participants with complicated grief demonstrated lower overall expressive flexibility than both asymptomatic bereaved participants and married controls. Nardelli et al. [56] found no clear associations between borderline symptoms and overall expressive flexibility, enhancement ability, or suppression ability after correction for multiple testing. Wang et al. [63], who examined only overall expressive flexibility, found that higher flexibility was associated with fewer drug-related problems at the bivariate level. Substance-use stigma was examined both as an aggregated score and across three dimensions: enacted stigma, referring to stigmatization experienced from others; anticipated stigma, referring to expected future stigmatization; and internalized stigma, referring to stigmatizing beliefs directed toward oneself. Expressive flexibility attenuated the associations of aggregated and internalized stigma with drug-related problems but did not moderate the corresponding associations for enacted or anticipated stigma.

Taken together, higher overall expressive flexibility was generally associated with a lower psychopathological symptom burden across depressive, anxiety-related, stress-related, trauma-related, and broader distress outcomes. The evidence was most consistent for cross-sectional associations, whereas prospective and adjusted findings, as well as findings for less frequently investigated outcomes, were more variable. Enhancement and suppression abilities did not show a uniform pattern across outcome domains, and several studies identified indirect or conditional associations involving social support, context and feedback sensitivity, trauma exposure, discrimination, or stigma. Overall, the findings indicate that associations with expressive flexibility extend across multiple psychopathological domains while varying according to the outcome, analytic approach, and contextual conditions examined.

#### 3.4.2. Psychological Well-Being

Psychological well-being outcomes were examined in seven studies and comprised life satisfaction, positive affect, general well-being, and psychological health-related quality of life.

*Life satisfaction* was the most frequently investigated outcome and was assessed using the Satisfaction With Life Scale (SWLS) in four studies [32,34,45,51]. Higher overall expressive flexibility was cross-sectionally associated with greater life satisfaction in three studies [32,34,51]. In Ang and Tsai [45], a concurrent positive association was observed among European American students, but not among Asian American or Latinx American students, and overall expressive flexibility did not predict subsequent life satisfaction in any of the three groups after adjustment for baseline life satisfaction and covariates.

Component-level and conditional findings further differentiated the associations with life satisfaction. Both enhancement and suppression abilities were cross-sectionally associated with greater life satisfaction in Chen et al. [32] and Ang and Tsai [51]. In Chen et al. [32], however, only enhancement ability remained independently associated after adjustment, and its association with life satisfaction was evident at higher, but not lower, levels of suppression ability. Prospectively, enhancement ability predicted greater subsequent life satisfaction only among Asian American students, whereas suppression ability showed no prospective association in any of the three cultural groups [45]. Ang and Tsai [51] further found that the association between overall expressive flexibility and life satisfaction was evident among young and middle-aged adults but not among older adults; the association with suppression ability was absent only among older men. Finally, Shangguan et al. [34] found that perceived social support partially mediated the positive association between overall expressive flexibility and life satisfaction.

*Positive affect* and *general well-being* were each examined in one study. Using the Positive and Negative Affect Schedule (PANAS), Ang and Tsai [51] found that higher overall expressive flexibility, enhancement ability, and suppression ability were each associated with greater positive affect. The associations involving overall expressive flexibility and suppression ability varied by age and gender: they were evident among young and middle-aged men and women but not among older adults, whereas the association involving enhancement ability did not differ across these groups. General well-being was assessed using the World Health Organization-Five Well-Being Index (WHO-5) by Gonzalez-Escamilla et al. [33]. After adjustment for age and gender, overall expressive flexibility and suppression ability showed positive associations with general well-being, but neither met the conventional threshold for statistical significance (*p* = 0.071 and *p* = 0.089, respectively). The estimated association between enhancement ability and general well-being was close to zero (*r* = 0.034, *p* = 0.615).

*Psychological health-related quality of life* was examined in two studies using the Mental Component Summary of the 12-item Short Form Health Survey (SF-12 MCS) [58,59]. Quattropani et al. [58] examined enhancement and suppression abilities separately but did not analyze overall expressive flexibility. In adjusted analyses, greater suppression ability was associated with better psychological health-related quality of life, whereas greater enhancement ability was associated with lower psychological health-related quality of life; the interaction between the two abilities was not significant. In Sardella et al. [59], higher overall expressive flexibility and suppression ability were associated with better psychological health-related quality of life at baseline, whereas the positive association with enhancement ability did not meet the conventional threshold for statistical significance (*p* = 0.090). Baseline overall expressive flexibility also predicted better psychological health-related quality of life at follow-up after adjustment for cognitive status, frailty, and dispositional optimism.

Taken together, higher overall expressive flexibility was generally associated with greater psychological well-being. Findings for positive affect, general well-being, and psychological health-related quality of life were based on fewer studies but predominantly indicated positive associations. The respective contributions of enhancement and suppression abilities varied across outcomes, and several associations differed according to age, gender, cultural group, or the level of the complementary ability. Prospective findings were mixed, with evidence of longitudinal associations for life satisfaction and psychological health-related quality of life in specific studies or population groups.

#### 3.4.3. Resilience and Adaptive Functioning

Resilience and adaptive functioning outcomes were examined in nine studies and comprised hope, resilience, ego-resilience, long-term psychological adjustment, burnout, and physical health-related quality of life.

*Hope*, *resilience*, and *ego-resilience* were investigated in four studies using the Adult Trait Hope Scale, the 11-item Resilience Scale (RS-11), the Connor–Davidson Resilience Scale (CD-RISC), and the Ego Resiliency Scale, respectively [26,32,33,51]. Across all four studies, higher overall expressive flexibility, enhancement ability, and suppression ability were associated with greater hope, resilience, or ego-resilience. In Ang and Tsai [51], the association between suppression ability and hope was evident across the examined age and gender groups except among older men, whereas the associations involving overall expressive flexibility and enhancement ability did not differ across these groups.

*Long-term adjustment* under conditions of stress was examined prospectively by Bonanno et al. [25] and concurrently within a three-year follow-up of a partially overlapping cohort by Westphal et al. [29]. Bonanno et al. [25] assessed psychological distress using a 32-item version of the Symptom Checklist-90-Revised (SCL-90-R) shortly after participants began college and in the immediate aftermath of the September 11 terrorist attacks, and again at the end of their second academic year. Higher overall expressive flexibility, enhancement ability, and suppression ability each predicted lower distress at follow-up after statistical adjustment for initial distress and cognitive task performance. In a partially overlapping sample assessed three years after the initial expressive-flexibility assessment, Westphal et al. [29] measured adjustment using close-friend ratings of participants’ psychological and physical health, social functioning, goal attainment, and coping ability. Higher overall expressive flexibility was associated with better adjustment at higher levels of cumulative life stress, particularly when expressive flexibility was assessed under an immediate threat condition. Enhancement ability was associated with better adjustment under high cumulative stress in the neutral condition only, whereas suppression ability was associated with better adjustment under high cumulative stress in both conditions, with a stronger association under immediate threat.

*Burnout* was examined in one study of palliative home care practitioners using the Maslach Burnout Inventory (MBI), which assesses emotional exhaustion, depersonalization, and personal accomplishment [52]. Higher overall expressive flexibility was associated with lower emotional exhaustion after statistical adjustment for profession, age, work experience, and context sensitivity. No associations were observed with depersonalization or personal accomplishment.

*Physical health-related quality of life* was examined in two studies using the Physical Component Summary of the 12-item Short Form Health Survey (SF-12 PCS) [58,59]. Quattropani et al. [58] examined enhancement and suppression abilities separately but did not analyze overall expressive flexibility. Neither ability nor their interaction was associated with physical health-related quality of life after statistical adjustment for age, gender, and trait emotional intelligence. In Sardella et al. [59], greater suppression ability was associated with better physical health-related quality of life at baseline and remained independently associated after statistical adjustment for gender, education, frailty, and dispositional optimism. Baseline suppression ability also predicted better physical health-related quality of life at follow-up, whereas enhancement ability and overall expressive flexibility showed no clear associations at baseline.

Taken together, higher overall expressive flexibility and its component abilities were associated with greater hope, resilience, and ego-resilience, as well as better long-term adjustment. The longitudinal findings indicated that expressive flexibility predicted subsequent adjustment, while the context-sensitive findings suggested that its adaptive relevance was particularly evident under higher levels of cumulative stress. Findings for burnout and physical health-related quality of life were more specific: overall expressive flexibility was associated only with emotional exhaustion among the examined burnout dimensions, whereas suppression ability, rather than enhancement ability or overall expressive flexibility, predicted better physical health-related quality of life. Thus, the associations with resilience and adaptive functioning varied according to the specific outcome and contextual conditions examined.

#### 3.4.4. Social Functioning

Social functioning outcomes were examined in six studies and comprised perceived social support, social functioning deficits, peer acceptance, friendship quality, and relationship stress.

*Perceived social support* was assessed in two studies using the Brief 2-Way Social Support Scale and the Perceived Social Support Scale, respectively [34,51]. Higher overall expressive flexibility was associated with greater perceived social support in both studies. Ang and Tsai [51] additionally found positive associations for enhancement and suppression abilities. The association involving enhancement ability did not differ across age and gender groups, whereas the associations involving overall expressive flexibility and suppression ability were evident among women of all examined age groups and among young and middle-aged men. Among older men, overall expressive flexibility was not associated with perceived social support, and suppression ability was associated with lower perceived social support.

*Social functioning deficits* and *peer acceptance* were each examined in one study. Using the Life Functioning Questionnaire (LFQ), Burton and Bonanno [26] found that higher overall expressive flexibility, enhancement ability, and suppression ability were each associated with fewer social functioning deficits. Wang et al. [43] assessed peer acceptance using a liking scale in an experimental study with adolescents. Participants assigned higher liking ratings to peers who demonstrated both enhancement and suppression abilities than to peers who demonstrated only one of the two abilities. This pattern did not differ according to participant sex.

Complementary contextual evidence came from Experiment 2 of the same publication, which examined social influences on expressive behavior in an independent sample of 100 adolescents [43]. Participants randomly assigned to peer exclusion displayed less emotional expression under enhancement and natural-expression instructions than those assigned to inclusion, whereas expression under suppression instructions did not differ between groups. These findings concern the effects of social experiences on subsequent expressive behavior, rather than effects of expressive flexibility on social functioning.

*Friendship quality* was examined longitudinally across three waves using the Friendship Quality Questionnaire in Wang et al. [62]. Higher overall expressive flexibility, enhancement ability, and suppression ability were associated with greater friendship quality both concurrently and across measurement waves, with the strongest associations observed for enhancement ability. However, none of the three expressive-flexibility variables independently predicted subsequent friendship quality in the unconditional cross-lagged models. Their prospective associations depended on social anxiety: overall expressive flexibility and enhancement ability predicted greater subsequent friendship quality only at comparatively low levels of social anxiety. Suppression ability showed a similar pattern at particularly low levels of social anxiety but predicted lower subsequent friendship quality at extremely high levels of social anxiety.

*Relationship stress* was assessed using the relationship-stress subscale of the College Student Stress Scale in Zhao and Wang [65]. Higher overall expressive flexibility, enhancement ability, and suppression ability were each associated with lower relationship stress. When the pleasant- and unpleasant-emotion scores were considered simultaneously in statistically adjusted models, greater ability to enhance and suppress pleasant emotional expressions was associated with lower relationship stress, whereas the corresponding ability for unpleasant emotional expressions was associated with higher relationship stress. Greater balance-adjusted flexibility in enhancing and suppressing pleasant emotional expressions was also associated with lower relationship stress, whereas balance-adjusted flexibility for unpleasant emotional expressions showed no clear association.

Taken together, higher expressive flexibility was generally associated with more favorable social functioning, including greater perceived social support, fewer social functioning deficits, greater peer acceptance and friendship quality, and lower relationship stress. Experimental evidence further indicated that peers demonstrating both enhancement and suppression abilities received greater liking than peers demonstrating only one of these abilities. However, the prospective associations with friendship quality depended on social anxiety, and suppression ability showed less favorable associations among older men and adolescents with particularly high social anxiety.

### 3.5. Meta-Analytic Associations Between Expressive Flexibility and Mental Health Outcomes

Three outcome domains provided sufficient comparable data for quantitative synthesis. Reflecting the dual-factor perspective on mental health, the primary meta-analyses examined depressive symptoms and life satisfaction as indicators of the psychopathology and subjective well-being dimensions, respectively. Anxiety was examined exploratorily as the only additional psychopathological outcome for which a sufficient number of comparable studies were available, thereby allowing an exploratory assessment of the broader transdiagnostic relevance of expressive flexibility.

Some studies contributed to the narrative synthesis but did not provide effect estimates meeting the criteria for quantitative synthesis. Dwyer et al. [38] reported nonsignificant correlations without individual coefficients, precluding their inclusion in the meta-analyses. Zhu and Bonanno [39] reported adjusted associations involving changes in enhancement and suppression abilities rather than unadjusted correlations between overall expressive flexibility and mental health outcomes.

Studies contributing to the meta-analyses were drawn exclusively from non-clinical samples. The primary models pooled unadjusted concurrent correlations and therefore did not account for potential confounding, even where the original studies also reported adjusted analyses. The design- and outcome-specific JBI judgments summarized in Section 3.3 and detailed in the supplementary document “JBI critical appraisal of expressive flexibility studies” provide the methodological context for interpreting these pooled associations.

#### 3.5.1. Depressive Symptoms: Primary Meta-Analysis

**Figure 2:**
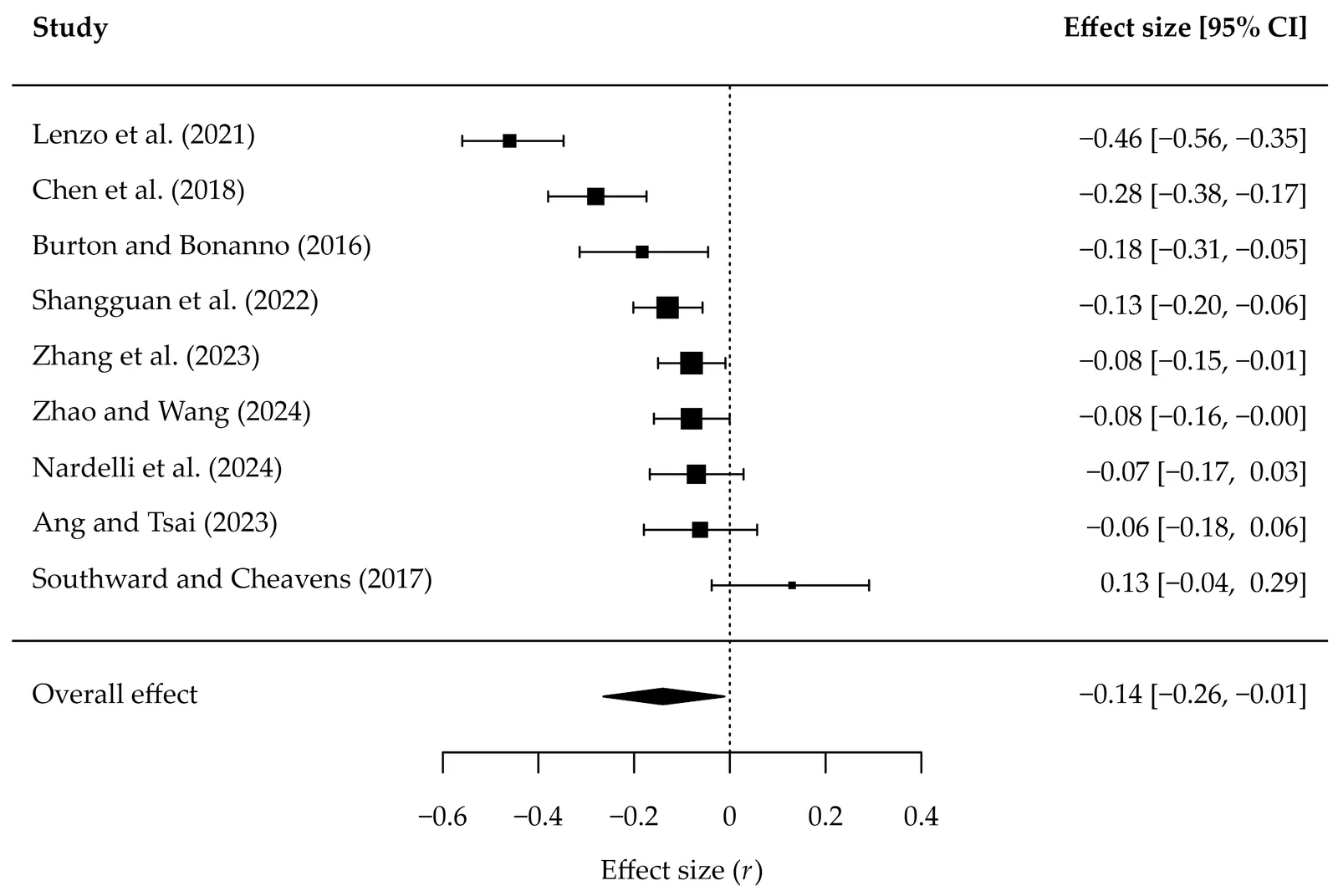
Forest plot for the meta-analysis of overall expressive flexibility and depressive symptoms. Study-specific unadjusted Pearson correlations and their 95% confidence intervals are represented by squares and horizontal lines, respectively; square size reflects study weight. The diamond represents the pooled random-effects estimate, and the dotted vertical line marks the null association (*r* = 0). Estimates are displayed as Pearson’s *r* after back-transformation from Fisher’s *z*. Included studies (from top to bottom in the forest plot) were Lenzo et al. [53], Chen et al. [32], Burton and Bonanno [26], Shangguan et al. [34], Zhang et al. [64], Zhao and Wang [65], Nardelli et al. [56], Ang and Tsai [45], and Southward and Cheavens [28].

Nine independent studies (*N* = 3622) provided eligible Pearson correlations between overall expressive flexibility and depressive symptoms. The pooled association was negative, *r* = −0.14, 95% CI [−0.26, −0.01], *p* = 0.037 (Figure 2). Heterogeneity was substantial, *Q*(8) = 51.91, *p* < 0.001, *I*^2^ = 90%, τ^2^ = 0.023, and the 95% prediction interval ranged from −0.47 to 0.23. Although the pooled mean association was negative, this interval encompassed null and positive associations, indicating that an inverse association cannot be assumed to generalize consistently across comparable populations and settings.

Leave-one-out estimates remained negative, ranging from *r* = −0.17 to *r* = −0.10, but the 95% confidence interval included zero in five of the nine iterations (*p* = 0.014–0.082 across all iterations). Influence diagnostics identified Lenzo et al. [53] as the only influential study; omitting it reduced the magnitude of the pooled association to *r* = −0.10, 95% CI [−0.19, −0.01], *p* = 0.031, and heterogeneity from *I*^2^ = 90% to *I*^2^ = 73%. Thus, the direction of the pooled association was consistent across omissions, whereas statistical significance was sensitive to the exclusion of individual studies.

The additional sensitivity models yielded negative pooled associations of similar magnitude. Including Spearman correlations yielded *r* = −0.15, 95% CI [−0.26, −0.03], *p* = 0.018 (*k* = 10); additionally including partial and partial-rank correlations yielded *r* = −0.16, 95% CI [−0.26, −0.05], *p* = 0.008 (*k* = 11). Replacing the baseline correlation from Southward and Cheavens [28] with the one-month prospective correlation yielded *r* = −0.15, 95% CI [−0.26, −0.04], *p* = 0.012 (*k* = 9). Heterogeneity remained substantial across these sensitivity models (*I*^2^ = 87–89%), and all corresponding 95% prediction intervals included zero.

#### 3.5.2. Life Satisfaction: Primary Meta-Analysis

**Figure 3:**
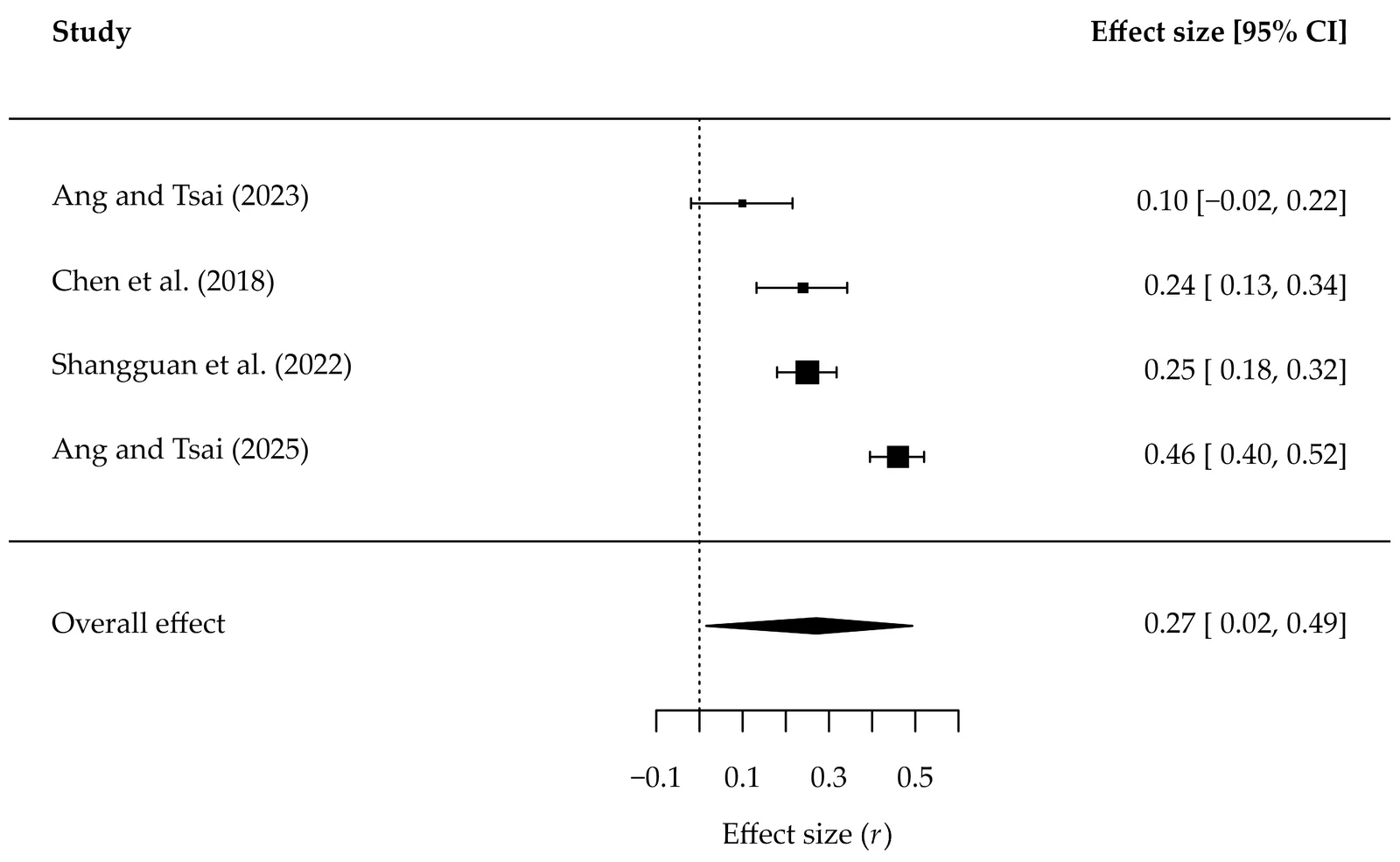
Forest plot for the meta-analysis of overall expressive flexibility and life satisfaction. Study-specific unadjusted Pearson correlations and their 95% confidence intervals are represented by squares and horizontal lines, respectively; square size reflects study weight. The diamond represents the pooled random-effects estimate, and the dotted vertical line marks the null association (*r* = 0). Estimates are displayed as Pearson’s *r* after back-transformation from Fisher’s *z*. Included studies (from top to bottom in the forest plot) were Ang and Tsai [45], Chen et al. [32], Shangguan et al. [34], and Ang and Tsai [51].

Four independent study units (*N* = 1910) provided correlations between overall expressive flexibility and life satisfaction. The pooled association was positive, *r* = 0.27, 95% CI [0.02, 0.49], *p* = 0.043 (Figure 3), but heterogeneity was very high, *Q*(3) = 36.64, *p* < 0.001, *I*^2^ = 92%, *τ*^2^ = 0.025, and the 95% prediction interval ranged from −0.28 to 0.69, indicating considerable variation across comparable populations and settings, with both inverse and substantially stronger positive associations remaining compatible with the evidence.

The estimate was fragile: leave-one-out estimates ranged from *r* = 0.20 to *r* = 0.32, and the Hartung–Knapp 95% confidence interval included zero in every iteration (all *p* > 0.05; range: 0.05045–0.13519). Influence diagnostics flagged two of the four study units (Ang and Tsai [45,51]) as influential. This primary meta-analysis should therefore be interpreted as preliminary evidence of a positive association of uncertain magnitude rather than as a robust pooled effect.

#### 3.5.3. Anxiety: Exploratory Meta-Analysis

**Figure 4:**
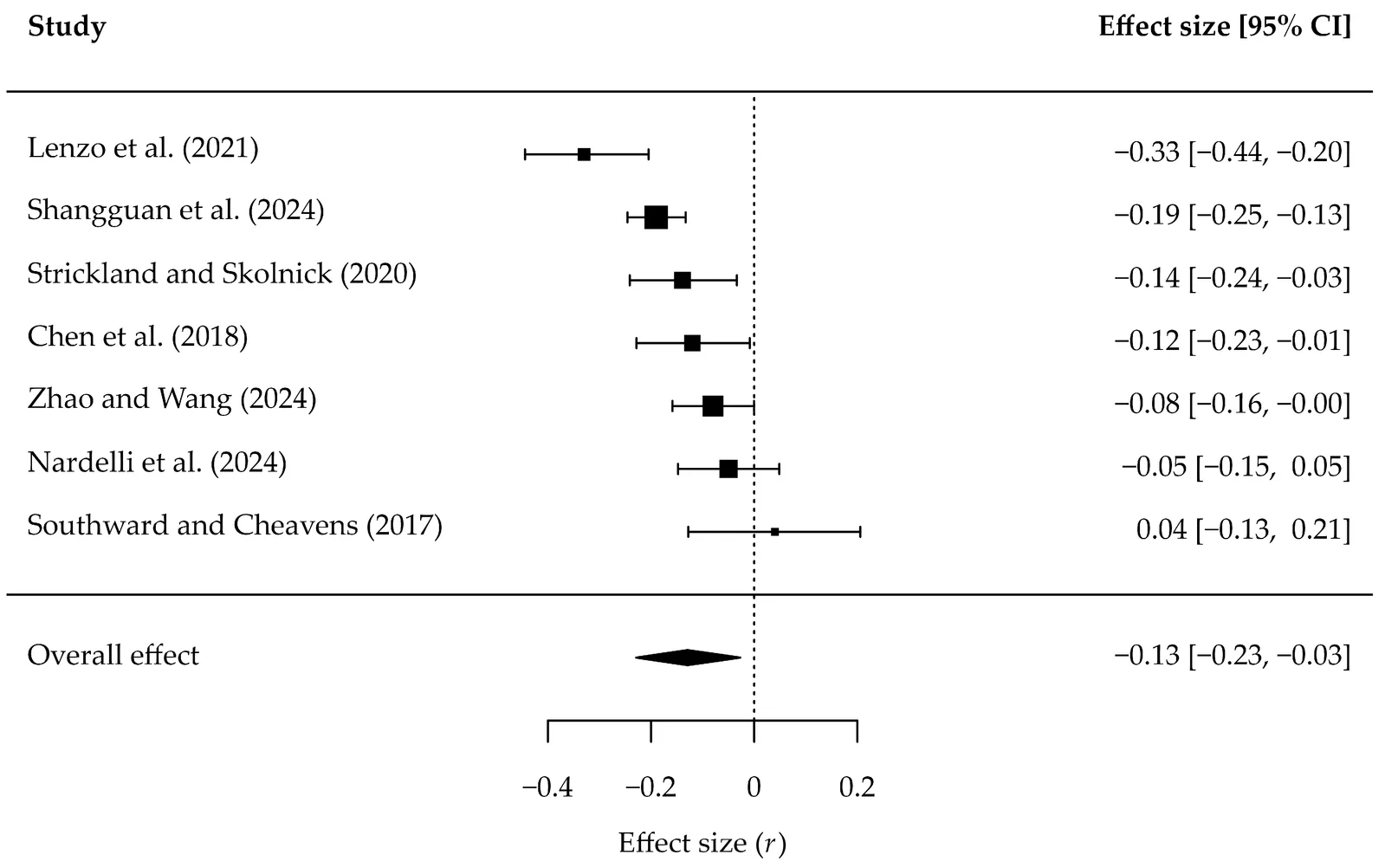
Forest plot for the exploratory meta-analysis of overall expressive flexibility and anxiety. Study-specific unadjusted Pearson correlations and their 95% confidence intervals are represented by squares and horizontal lines, respectively; square size reflects study weight. The diamond represents the pooled random-effects estimate, and the dotted vertical line marks the null association (*r* = 0). Estimates are displayed as Pearson’s *r* after back-transformation from Fisher’s *z*. Included studies (from top to bottom in the forest plot) were Lenzo et al. [53], Shangguan et al. [60], Strickland and Skolnick [46], Chen et al. [32], Zhao and Wang [65], Nardelli et al. [56], and Southward and Cheavens [28].

In the exploratory anxiety meta-analysis, seven independent study-level effects (*N* = 3134) yielded a pooled association of *r* = −0.13, 95% CI [−0.23, −0.03], *p* = 0.021 (Figure 4). Heterogeneity was substantial, *Q*(6) = 21.59, *p* = 0.001, *I*^2^ = 76%, *τ*^2^ = 0.008, and the 95% prediction interval ranged from −0.35 to 0.11. Although the pooled mean association was negative, this interval encompassed null and positive associations, indicating that an inverse association cannot be assumed to generalize consistently across comparable populations and settings.

Leave-one-out estimates remained negative, ranging from *r* = −0.15 to *r* = −0.11, but the 95% confidence interval included zero in two of the seven iterations (*p* = 0.014–0.064 across all iterations). No studies were flagged as influential by the influence diagnostics. Thus, the direction of the pooled association was consistent across omissions, whereas statistical significance was sensitive to the exclusion of individual studies.

Replacing the baseline correlation from Southward and Cheavens [28] with the one-month prospective correlation yielded *r* = −0.14, 95% CI [−0.22, −0.05], *p* = 0.008 (*k* = 7). When the model was restricted to state- or symptom-based anxiety outcomes, excluding the two trait-anxiety studies, the pooled association was *r* = −0.11, 95% CI [−0.27, 0.06], *p* = 0.141 (*k* = 5). Heterogeneity remained substantial in both sensitivity models (*I*^2^ = 68% and 81%, respectively), and both 95% prediction intervals included zero. The association with state- or symptom-based anxiety was therefore estimated imprecisely and should not be interpreted as robust evidence for anxiety symptoms specifically.

#### 3.5.4. Assessment of Reporting Bias

Descriptive inspection of the funnel plots for the depression and anxiety meta-analyses (Figures S1 and S2) did not indicate a clear pattern of asymmetry. The depression funnel plot nevertheless contained the influential and comparatively imprecise estimate reported by Lenzo et al. [53]. Given the small numbers of included studies, no formal conclusions regarding reporting bias or small-study effects were drawn.

### 3.6. Outcome-Level Appraisal of the Evidence

The outcome-level appraisal supported average concurrent associations of overall expressive flexibility with fewer depressive symptoms, greater life satisfaction, and, exploratorily, lower anxiety. Confidence in their magnitude and generalizability was limited by study-specific risks of bias, substantial heterogeneity, and imprecision. All three syntheses pooled unadjusted concurrent correlations from non-clinical samples. Leave-one-out point estimates retained their respective directions, although several confidence intervals included zero, including all four for life satisfaction. The anxiety findings additionally depended on the constructs included: the confidence interval for the analysis restricted to state- or symptom-based anxiety included zero. Potential publication and reporting bias remained unresolved.

For outcomes synthesized narratively, the appraisal identified convergent concurrent findings alongside more limited prospective and experimental evidence. Positive associations with resilience and perceived social support were each reported in two studies, whereas several other outcomes were represented by single studies. Prospective findings were outcome- and context-specific; for example, prospective friendship-quality associations depended on social anxiety. Experimental evidence for peer acceptance concerned immediate liking of observed targets displaying different expressive abilities. The complete outcome-specific appraisals, including supporting evidence and limitations, are provided in the supplementary document “Outcome-level appraisal of the evidence.”

## 4. Discussion

### 4.1. Broad but Outcome-Dependent Associations with Mental Health

To our knowledge, this is the first systematic review and meta-analysis to synthesize evidence on the associations between expressive flexibility and mental health. Across the 29 included studies, expressive flexibility was examined in relation to psychopathology, psychological well-being, resilience and adaptive functioning, and social functioning. Across these domains, higher overall expressive flexibility was generally associated with a lower psychopathological symptom burden, greater psychological well-being, better adaptive functioning, and better social functioning. However, the strength and consistency of these associations varied across outcome domains, study designs, and populations, and the respective contributions of enhancement and suppression ability were not uniform. This breadth of associations provides a rationale for investigating expressive flexibility as a potentially transdiagnostic dimension of emotion expression. However, associations across mental-health dimensions in predominantly non-clinical samples do not establish its relevance across psychiatric diagnoses.

The meta-analytic findings require cautious interpretation across all three outcomes. Reflecting the dual-factor perspective that guided the primary analyses [30,31], the pooled estimates indicated associations of higher overall expressive flexibility with fewer depressive symptoms and greater life satisfaction. The inverse association with depressive symptoms was small. Although its direction remained negative across all leave-one-out analyses and the additional sensitivity models yielded similar estimates, confidence intervals included zero in five of the nine leave-one-out iterations. The positive association with life satisfaction was particularly uncertain: only four studies contributed, confidence intervals included zero in every leave-one-out analysis, and two studies were identified as influential. The exploratory anxiety synthesis also yielded a small inverse association, but confidence intervals included zero in two leave-one-out iterations and in the analysis restricted to state- or symptom-based anxiety. Substantial heterogeneity and prediction intervals encompassing zero across all three outcomes further limit generalizability. These findings suggest associations with both psychopathological symptoms and positive mental health, but their magnitude and consistency across populations and settings remain uncertain.

The narrative synthesis extended these findings beyond the outcomes included in the quantitative analyses. Higher expressive flexibility was generally associated with fewer stress- and trauma-related symptoms and lower general psychological distress, as well as greater resilience, adaptive functioning, and social functioning. Particularly informative were findings obtained under conditions of adversity. Expressive flexibility predicted subsequent adjustment following the September 11 terrorist attacks [25] and was associated with better adjustment under higher levels of cumulative life stress [29], and attenuated the associations of repeated traumatic exposure with posttraumatic stress disorder symptoms [54], sexual orientation-related discrimination with psychological distress [61], and substance use stigma with drug-related problems [63]. These conditional findings are consistent with the possibility that expressive flexibility becomes especially relevant when individuals must adapt their emotional signaling to changing or socially demanding conditions. However, because these relationships were derived from a small number of heterogeneous and predominantly observational studies, they should be interpreted as evidence of context-dependent associations rather than causal protective effects.

A further recurring pattern concerned the distinction between overall expressive flexibility and its individual components. Overall expressive flexibility showed a more coherent direction of associations across outcome domains, whereas enhancement and suppression abilities showed distinct and, at times, opposing relationships with mental health. Their relevance depended in some studies on the level of the complementary ability [32], on whether expressions of pleasant or unpleasant emotions were considered [65], and on cultural or demographic characteristics [45,51]. The adaptive significance of expressive flexibility therefore cannot be reduced to a generally greater ability to enhance or suppress emotional expression. Rather, the evidence points to the importance of possessing capacities in both directions and applying them in relation to emotional, interpersonal, and situational demands. At the same time, the measures used in the included studies assessed whether participants could modify emotional expression after a regulatory direction had been specified experimentally or described in a hypothetical scenario; they did not directly establish whether individuals identified and selected the most appropriate response in everyday situations [26,27].

These measurement boundaries become particularly important when the findings are considered in relation to the broader emotion-regulation literature. This literature has increasingly shifted away from classifying individual regulatory strategies as inherently adaptive or maladaptive [27,66,67]. Although meta-analytic work has identified average associations of particular strategies with psychopathology [68], subsequent accounts have emphasized that the effects of a strategy vary with contextual demands, individual characteristics, and the available regulatory alternatives [27,66,67]. Together, these lines of work suggest that adaptive regulation is better understood in terms of flexible responding than the use of any single strategy. Bonanno and Burton articulated this broader perspective in a regulatory flexibility framework comprising three sequential components: sensitivity to contextual demands, access to a diverse repertoire of regulatory strategies, and responsiveness to feedback [27]. Bonanno later referred to these components collectively as the flexibility sequence [69]. Applied to the present findings, this framework offers a useful interpretation of the comparatively consistent direction of associations for overall expressive flexibility and the more variable associations for enhancement and suppression ability separately. Neither expressive direction appears uniformly adaptive; rather, expressive capacities need to be considered in conjunction with the recognition of contextual demands and the monitoring of their consequences. Consistent with this interpretation, Southward and Cheavens [28] found that expressive flexibility was prospectively related to symptom change conditionally as a function of context sensitivity and indirectly through feedback sensitivity. These findings suggest that understanding the mental-health relevance of expressive flexibility requires considering not only the capacity to enhance and suppress expression, but also how this capacity is deployed in response to contextual demands and feedback.

### 4.2. Theoretical Implications: Integrating Emotion Processing and Flexibility Research

#### 4.2.1. Implications for the Emotion Expression Function Within C-FEP

The findings have broader implications for the conceptualization of psychopathology within the C-FEP model. C-FEP differentiates impathy, empathy, emotion regulation, and emotion expression as distinct core functions while conceptualizing their coordination as central to adaptive emotion processing [6,7]. The present review adds evidence that individual differences within the core function of emotion expression—specifically, the capacity to modify the visibility of emotional responses in both directions—are associated with psychopathological symptoms, psychological well-being, adaptive functioning, and social functioning. These findings support examining expressive flexibility as a specific capacity within the emotion-expression function proposed by C-FEP. Initial evidence also indicates predictive value beyond habitual strategy use: Ang and Tsai [45] found that enhancement ability predicted subsequent life satisfaction among Asian Americans after adjustment for baseline life satisfaction, demographic characteristics, and the frequency of cognitive reappraisal and expressive suppression. This finding provides a context-specific indication of incremental validity, while broader evidence is needed to determine whether expressive flexibility contributes to mental health beyond other emotion-regulatory capacities. At the same time, associations varied across outcomes, populations, and contextual conditions, suggesting that the mental-health relevance of expressive flexibility is not uniform. The C-FEP model proposes that the core functions have transdiagnostic relevance while showing differential patterns of association across mental health outcomes and psychological conditions [6]. Within the domain of emotion expression, the observed associations with multiple aspects of mental health and the variation across outcomes and expressive components motivate further investigation of this proposition. In particular, they provide a rationale for testing whether distinct profiles of expressive capacities are associated with different symptom dimensions and diagnostic groups. These findings do not yet establish corresponding profiles across all four core functions or determine whether the observed associations reflect vulnerabilities, consequences, or reciprocal relationships with psychological difficulties.

Beyond these patterns of association, C-FEP provides a theoretical account of how expressive flexibility may interact with other core functions of emotion processing. Adjusting emotional expression to situational demands may draw on impathy to identify and understand one’s own emotional state and on empathy to perceive and understand the emotional states of others. Context sensitivity should not be equated with either perceptual function but may depend partly on the information they provide. Emotion regulation can shape the emotional response from which expression arises, whereas expressive behavior can feed back into intrapersonal experience and alter the emotional and behavioral responses of others [3,6,7,8,10,11,12,13,15,16,17]. Expressive flexibility may therefore be embedded in reciprocal processes through which emotion perception informs expression and the consequences of expression provide new information for subsequent perception, regulation, and expressive adjustment. The included literature offers preliminary points of connection with this account: several studies assessed context sensitivity, feedback sensitivity, or emotion-regulation processes alongside expressive flexibility [28,52,53,60,65], but none directly operationalized all four C-FEP functions or their temporal interplay. The proposed coordination among the core functions therefore represents a theoretically supported but as yet largely untested direction for future research.

#### 4.2.2. Linking C-FEP to the Flexibility Sequence

Integrating C-FEP with the flexibility sequence provides complementary perspectives on flexible emotion processing. The flexibility sequence describes how adaptation unfolds over time through the evaluation of contextual demands, the selection and implementation of a response, and the monitoring of its effectiveness [27,69]. C-FEP, in turn, specifies the emotion-processing functions that may contribute information and response capacities during these stages [6,7]. During context evaluation, impathy and empathy may provide information about one’s own emotional state and the emotional states of others. During response selection and implementation, emotion regulation may influence emotional experience and expression, while expressive flexibility provides the capacity to enhance or suppress observable expression. During feedback monitoring, changes in one’s own emotional state and in the responses of others can be perceived and used to maintain, adjust, or replace the selected response. The two frameworks thus offer complementary perspectives: the flexibility sequence organizes flexible responding over time, whereas C-FEP distinguishes the functions whose coordination may support this process.

This integration also clarifies the scope of the measures predominantly used in expressive-flexibility research. In the experimental paradigm, participants are explicitly instructed whether to enhance or suppress their expression, whereas the FREE Scale assesses perceived ability across hypothetical situations organized according to a specified expressive direction [25,26]. Although the FREE Scale incorporates situational content, neither approach directly assesses whether individuals independently recognize which expressive response is appropriate, select that response, or revise it after receiving feedback [27,69]. These measures therefore primarily capture the capacity to implement opposing forms of expressive modulation rather than all components of the flexibility sequence. Importantly, integrated approaches have already examined the sequence itself. Chen and Bonanno identified profiles based on context sensitivity, repertoire, and feedback responsiveness [70]; Robinson et al. tested the proposed sequential pathway using mediation models [71]; and Chen et al. used repeated daily assessments over 21 days to derive proxy measures of the three components based on how participants differentiated everyday situations, used personally effective emotion-regulation strategies, and maintained effective or changed ineffective strategies [72]. These studies provide a methodological foundation for examining the flexibility sequence as a coordinated process.

At the same time, several questions remain unresolved, including which emotional information informs context sensitivity, why access to a broad repertoire does not necessarily result in an appropriate response, and which internal and interpersonal consequences are used as feedback [27,70,71,72]. The integrative perspective on emotion processing provided by the C-FEP model offers a framework for addressing these questions by systematically examining impathy, empathy, emotion regulation, and emotion expression within the flexibility sequence [6,7]. This approach could clarify how the perception and understanding of one’s own and others’ emotional states guide the evaluation of situational demands, how emotion-regulatory and expressive capacities jointly shape response selection and implementation, and how the consequences of a response inform subsequent adjustment. Chen et al. found that switching away from ineffective strategies was associated with lower momentary depressed mood but not lower anxious mood [72]. From a C-FEP perspective, this pattern raises the possibility that changing responses alone may be insufficient when relevant emotional cues are misinterpreted or when the alternative response does not fit the intrapersonal or interpersonal demands of the situation.

### 4.3. Practical Implications for Psychotherapy and Coaching

The findings suggest that expressive flexibility merits attention in research on psychotherapy and coaching, but they do not establish that increasing this capacity improves mental health. A clinically relevant question is whether clients can modify emotional expression in both directions and how this capacity relates to their difficulties, situational demands, and interpersonal goals. The following considerations translate this question into proposals for observation and assessment whose validity and clinical utility remain to be established. Bonanno’s flexibility sequence provides a process framework for this perspective by distinguishing context sensitivity, access to an expressive or regulatory repertoire, and responsiveness to feedback [27,69]. The C-FEP model offers a complementary functional perspective: impathy and empathy provide information about one’s own and others’ emotional states, emotion regulation modulates the emotional response, and emotion expression communicates aspects of that response [6,7]. Thus, the flexibility sequence describes the stages through which adjustment unfolds, whereas C-FEP identifies the core functions of emotion processing that may support or constrain these stages.

Recent research provides an empirical starting point for examining expressive dynamics during emotionally salient conversations. Hilberdink et al. [73] used computer vision to analyze 168 video-recorded interviews with 133 emergency healthcare workers discussing stressful work experiences. Temporal features of facial expressivity distinguished profiles characterized by differing levels of psychological symptoms and resilient coping. The authors interpreted these features as potential behavioral proxies of expressive flexibility. This approach demonstrates that dynamic expressive behavior during interviews can provide information relevant to mental health and motivates investigating comparable observations in psychotherapy and coaching. Whether these markers capture the capacity to deliberately enhance and suppress expression, correspond to established expressive-flexibility measures, or provide clinically useful information in therapeutic encounters remains to be tested.

Emotionally activating sequences in psychotherapy and coaching offer opportunities to extend this line of research to therapeutic conversations. As clients describe, revisit, and reflect on personally significant experiences, changes in expressive behavior can be examined alongside changes in subjective experience and interpersonal interaction. Process-oriented approaches provide examples of how such observation can be embedded in ongoing therapeutic work. In Eye Movement Desensitization and Reprocessing (EMDR), changes in images, cognitions, emotions, and bodily sensations are repeatedly monitored during memory processing [74]. In integrative emotion coaching with emTrace, this ongoing observation also encompasses nonverbal expression [75,76]. Likewise, process monitoring has been described in clinical hypnosis, including the use of physiological measures to track changes in autonomic arousal [77]. This attention to unfolding experience provides a point of connection for considering expressive flexibility: how clients vary the visibility and intensity of emotional expression as emotional and interpersonal demands change. A C-FEP-informed perspective places these observations alongside the perception of one’s own and others’ emotional states and the regulation of emotional responses, providing a framework for considering how these functions may interact during psychotherapy and coaching [6,7].

Because emotional expression unfolds dynamically across facial, vocal, postural, gestural, and other bodily channels, the assessment of expressive behavior requires trained multimodal observation [16,17,18]. Accordingly, a structured observational approach should account for multiple channels, including facial movement, head movement, gestures, leg and foot behavior, posture, psychophysiological cues, voice, and interpersonal movement [78]. Putting such an approach into practice depends on practitioners’ ability to recognize relevant emotional cues across these modalities. Evidence indicates that this ability can be improved through brief training combining instruction, practice, and feedback [79]. With this observational foundation, expressive behavior relevant to flexibility could be examined during emotionally salient sequences that already occur in psychotherapy and coaching—for example, when clients initially describe a situation, process the associated experience, respond to the practitioner, and later return to the initial focus.

Beyond naturalistic observation, the experimental paradigm underlying expressive-flexibility research suggests a more direct, process-integrated behavioral probe [25]. When an emotionally salient experience is already activated, and such prompting is clinically appropriate, clients could be invited, in separate prompts, to make their expression more visible and to reduce its outward intensity. The extent to which they can alter their expression in each direction relative to its spontaneous presentation could provide within-person information about the enhancement and suppression abilities central to the operationalization of expressive flexibility. Repeating this procedure at selected points during psychotherapy or coaching could permit a preliminary examination of change over time. At present, however, this proposal should be regarded as a hypothesis-generating adaptation of the experimental paradigm rather than a validated clinical assessment procedure. Moreover, it would assess instructed expressive modulation, not whether clients identify the contextually appropriate response or revise it in response to feedback.

Taken together, naturalistic observation and prompted modulation may generate complementary hypotheses about whether clients allow emotion to become more or less visible, can deliberately alter its outward intensity in both directions, or adjust their expression in response to internal or interpersonal feedback. These observations may also guide subsequent inquiry into whether the form and intensity of the expression corresponded to the client’s emotional experience, the situational demands, and the intended interpersonal goal. From a C-FEP perspective, this could help clarify whether apparent expressive rigidity primarily reflects limitations in emotion expression itself or difficulties in perceiving one’s own emotional state, understanding another person’s emotional state, or regulating emotional arousal [6,7]. An important next step is to establish whether naturalistic observation and the proposed behavioral probe provide reliable and valid information about expressive flexibility and add clinically useful information beyond the FREE Scale and the experimental expressive-flexibility paradigm [25,26]. Interpretations should remain provisional and account for habitual expressiveness, current emotional activation, the therapeutic relationship, and cultural context. Although research indicates substantial cross-cultural similarities in emotional expression, cultural display rules shape when, where, and to what extent particular emotions are expressed [16,17,18,80,81,82]. Cultural background must therefore be considered when evaluating expressive flexibility, because the same degree of enhancement or suppression may reflect context-sensitive adjustment in one cultural setting but not in another.

### 4.4. Methodological Quality and Limitations

The JBI appraisals identified distinct methodological limitations across the included evidence. Inconsistent control of confounding means that some observed associations may partly reflect demographic, clinical, or contextual characteristics rather than a specific relationship between expressive flexibility and the outcome. This concern also applies to the primary meta-analyses, which pooled unadjusted correlations. In clinical and mixed samples, psychotherapy and psychotropic medication represent additional potential confounders. Eligibility restrictions concerning medication do not by themselves establish that treatment-related influences on emotional expression or symptom severity have been accounted for. For longitudinal contributions, incomplete follow-up and the absence of reported strategies addressing missing follow-up data leave uncertainty about selective retention. Uncertainty concerning particular instrument versions, scoring procedures, and unmodeled dependence among participants further limits interpretation of some findings. In the randomized experiment [43], reporting gaps restricted the assessment of several safeguards against bias. These limitations differ across studies and analyses; neither affirmative item judgments nor uncertainty due to incomplete reporting should be interpreted as an overall verdict on a study’s validity.

The studies included in this review showed meaningful geographic and developmental breadth, comprising samples from nine countries and spanning adolescence through older adulthood. Representation was nevertheless uneven: 23 of the 29 studies were conducted in the United States, China, or Italy, and 24 examined exclusively non-clinical populations. Because eligibility was restricted to participants aged 12 years or older, the findings cannot be generalized to younger age groups. Generalizability may also be more limited for clinical populations and cultural contexts that remain underrepresented. This qualification is particularly relevant because individual studies indicated that associations involving expressive flexibility may vary across cultural groups or according to collectivist values [45,46].

Further limitations concern study design. Of the 29 studies, 22 contributed exclusively cross-sectional analyses, while 6 contributed longitudinal analyses, including five that also contributed cross-sectional analyses; one contributed experimental analyses. The predominance of cross-sectional evidence limits conclusions about temporal direction and precludes causal interpretations of the observed associations.

A central measurement issue concerns the distinction between perceived and demonstrated expressive capacity. Twenty of the twenty-nine studies assessed expressive flexibility through self-report, whereas nine used the performance-based experimental paradigm. This imbalance was also evident in the quantitative syntheses: all four studies included in the life-satisfaction meta-analysis used self-report measures, as did eight of the nine studies in the depression meta-analysis and six of the seven studies in the anxiety meta-analysis. The available data therefore did not permit a meaningful comparison of pooled associations across measurement approaches. Associations between self-reported expressive flexibility and self-reported mental health outcomes may also be affected by shared method variance and primarily reflect perceived rather than demonstrated expressive capacity. Evidence from Burton and Bonanno [26] is particularly relevant in this context, as it indicates that perceived and demonstrated abilities are meaningfully related. Specifically, higher overall FREE scores were associated with greater expressive flexibility in the experimental paradigm. At the same time, Burton and Bonanno [26] emphasized that the correspondence between the two measurement approaches was only moderate (*r* = 0.284 for overall expressive flexibility) and that self-report did not capture a considerable proportion of observed regulatory performance. They suggested that this divergence may partly reflect differences between the socially embedded situations represented in the FREE Scale and the laboratory task, which did not involve direct interaction with the person toward whom expression was being regulated. Self-report and performance-based measures should therefore be regarded as complementary rather than interchangeable approaches to expressive flexibility.

The quantitative evidence was also limited in scope and precision. Although 29 studies were included in the systematic review, the two primary quantitative syntheses comprised nine independent study units for depressive symptoms and four for life satisfaction, while the exploratory anxiety meta-analysis comprised seven. The overall number of included studies therefore should not be taken as the evidence base for any individual pooled estimate. The life-satisfaction synthesis was particularly limited by its small number of contributing studies, leaving both the pooled association and the estimated between-study heterogeneity uncertain. The use of REML estimation and Hartung–Knapp inference does not overcome these limitations of the evidence base. Although these methods account for between-study heterogeneity and uncertainty in estimating the pooled mean, they cannot compensate for the small number of contributing studies or establish that the pooled association generalizes across populations and settings. The small numbers of studies in each synthesis also precluded subgroup analyses or meta-regressions to investigate potential sources of the substantial between-study heterogeneity. Moreover, the prediction intervals for all three outcomes included zero, indicating that the pooled estimates should not be interpreted as uniformly applicable across populations and settings. Potential reporting bias and small-study effects could be examined only descriptively for depression and anxiety and could not be meaningfully assessed for life satisfaction. The absence of clear funnel-plot asymmetry should therefore not be interpreted as evidence that such biases were absent. Because the specific outcomes for meta-analysis were selected after data extraction and no adjustment for multiplicity was applied, the three syntheses should not be interpreted as prespecified confirmatory tests of the review hypotheses.

Limitations also arise from the review process. The electronic search covered three databases and used two construct-specific expressions, which may have missed relevant studies indexed elsewhere or described using alternative terminology. The foundational study by Bonanno et al. [25] introduced the experimental operationalization of expressive flexibility but did not use either exact search phrase in its title or abstract, although “expressive flexibility” appeared in the full text. Its identification through citation searching illustrates the complementary value of this approach when relevant constructs are described using different terminology in searchable fields. Citation searching and expert consultation supplemented the database searches, but the possibility that other eligible studies using alternative terminology were missed cannot be excluded. Restriction to English-language, peer-reviewed journal articles also excluded potentially relevant evidence published in other languages or outside peer-reviewed journals. These restrictions may introduce language and publication bias, whose extent could not be determined from the available evidence.

### 4.5. Future Research Directions

Future research should prioritize longitudinal, experimental, and intervention-based designs that permit stronger conclusions about temporal and causal relationships. Repeated assessments of expressive flexibility and mental health could clarify whether expressive flexibility predicts subsequent changes in mental health, is itself affected by psychological symptoms, or participates in reciprocal processes over time. Studies in well-characterized clinical and high-risk populations are particularly needed to determine whether the observed associations extend beyond predominantly non-clinical samples. Direct comparisons across diagnostic groups, alongside dimensional assessments of symptom severity, should test whether associations involving overall expressive flexibility, enhancement ability, and suppression ability are shared across disorders or show differential patterns. Such studies should account for comorbidity and treatment exposure to clarify whether any differences are associated with diagnostic status, symptom burden, or treatment-related influences. Greater cultural diversity and multisite designs would further help distinguish generalizable associations from patterns that depend on culturally embedded expressive demands.

Measurement research should combine self-report and performance-based assessments within the same samples rather than treating them as interchangeable indicators. Such multimethod designs could clarify the degree to which perceived expressive capacity corresponds to demonstrated enhancement and suppression ability and whether each approach explains distinct variation in mental health outcomes [25,26]. Ecological momentary assessment and repeated observation of multimodal expressive behavior could additionally examine how these capacities are implemented in everyday situations. Importantly, future measures should distinguish the ability to follow a specified expressive direction from the ability to recognize contextual demands, select an appropriate response, and adjust it in response to subsequent feedback [27,69]. Expanding the evidence base for the reliability and convergent, discriminant, incremental, and predictive validity of these measures, including tests of measurement invariance across age groups and cultural contexts, will be essential for clarifying what different measures of expressive flexibility capture.

Future studies should also examine expressive flexibility as part of a coordinated emotion-processing system rather than as an isolated capacity. Simultaneously assessing the four C-FEP core functions and the components of the flexibility sequence could clarify how impathy and empathy contribute to the evaluation of contextual demands, how emotion regulation and emotion expression jointly shape response implementation, and how internal and interpersonal consequences inform subsequent adjustment [6,7,27,69]. Experimental and longitudinal designs would be particularly useful for examining these processes within individuals and determining whether their coordination differs across psychological conditions. At the same time, such research could test the C-FEP proposition that the core functions are transdiagnostically relevant while exhibiting differential patterns across mental health outcomes and disorders.

The developmental foundations of expressive flexibility remain largely unexplored. Initial evidence comes from Pitur and Miu [57], who found that childhood abuse, but not neglect, showed an independent inverse association with expressive flexibility. Although the retrospective and cross-sectional design does not establish a developmental pathway, this finding identifies early adverse interpersonal experiences as a potentially relevant antecedent. Attachment research provides a particularly relevant framework for investigating this relationship because systematic reviews of studies using the Adult Attachment Interview (AAI) and the Adult Attachment Projective Picture System (AAP) have documented attachment-related patterns across impathy, empathy, emotion regulation, and emotion expression [7,83]. However, none of the included studies directly examined attachment representations in relation to expressive flexibility. Longitudinal research beginning in early childhood should therefore investigate whether attachment patterns help explain how early interpersonal experiences relate to the development of expressive flexibility and to later mental health outcomes. Complementary studies in adults could examine whether attachment representations assessed using the AAI or AAP are associated with perceived and experimentally demonstrated expressive flexibility.

Intervention research represents another important priority. Randomized studies should examine whether enhancement and suppression abilities can be strengthened, whether such changes transfer to everyday situations, and whether they precede improvements in mental health. Because expressive flexibility captures the capacity to implement a specified expressive direction, interventions intended to promote its adaptive use should additionally address context sensitivity and responsiveness to feedback [27,69]. Expressive flexibility and the broader components of the flexibility sequence should therefore be assessed separately to determine whether changes in contextual evaluation and feedback monitoring facilitate the effective use of expressive capacities. The process-integrated behavioral probe proposed above would also require validation against the FREE Scale and the established experimental paradigm before it could be used to evaluate change in psychotherapy or coaching.

A further research opportunity arises from interventions that temporarily constrain specific components of the expressive repertoire. Reviews of upper- and lower-face botulinum toxin type A (BoNT-A) treatments have identified expressive flexibility as a relevant but insufficiently investigated outcome [84,85]. Recent longitudinal evidence showed that, at the peak effect of upper-face BoNT-A treatment, anger and surprise were less clearly conveyed to external observers when treated individuals attempted to express these emotions, whereas observer attributions for joy and neutrality remained stable [86]. Such treatments provide a distinctive model for examining whether anatomically specific restrictions limit enhancement or suppression ability and whether individuals compensate through unaffected facial regions or other expressive channels. Combining assessments before, during, and after the treatment effect with the FREE Scale, the experimental expressive-flexibility paradigm, and multimodal observation could clarify how alterations in expressive capacity affect intrapersonal experience, interpersonal signaling, and mental health.

## 5. Conclusions

This systematic review and meta-analysis provide the first comprehensive synthesis of the relationship between expressive flexibility and mental health. Across 29 studies, greater overall expressive flexibility was generally associated with lower psychopathological symptom burden and higher psychological well-being, resilience, adaptive functioning, and social functioning. The primary meta-analyses indicated that greater overall expressive flexibility was associated with fewer depressive symptoms and, provisionally, greater life satisfaction; the exploratory anxiety synthesis also indicated a small inverse association. The direction of these associations was consistent across leave-one-out analyses, although their statistical significance was sensitive to individual study omissions. Substantial heterogeneity and prediction intervals encompassing zero indicate that the strength and generalizability of these associations remain uncertain. Variation across studies may reflect contextual differences alongside differences in populations, measures, and methodological limitations. The differing associations of enhancement and suppression ability underscore the importance of considering both expressive directions when examining the mental-health relevance of expressive flexibility. C-FEP provides a theoretical framework for situating these findings within emotion expression as a core function of emotion processing and for developing hypotheses about differential functional profiles. The breadth of observed associations motivates investigating the potential transdiagnostic relevance of expressive flexibility, while its generalizability across psychiatric diagnoses and its coordination with other core functions remain to be established.

Taken together, this review integrates evidence on expressive flexibility as a mental-health-relevant capacity and provides an initial quantitative synthesis of its associations with depressive symptoms, life satisfaction, and anxiety. It identifies priorities for longitudinal, multimethod, and experimental research to clarify temporal relationships, contextual variation, and applicability across clinical and cultural settings.

## Figures and Tables

**Table 1:** Database-specific search strategies.

Database	Search Fields	Search String
PsycINFO	Title OR Abstract OR Keywords	“expressive flexibility” OR “flexible regulation of emotional expression”
PsycArticles	Title OR Abstract OR Keywords	“expressive flexibility” OR “flexible regulation of emotional expression”
PubMed	Title/Abstract	“expressive flexibility”[Title/Abstract] OR “flexible regulation of emotional expression”[Title/Abstract]

**Table 2:** Eligibility criteria according to the Population–Exposure–Outcome framework.

PEO Component	Eligibility Criterion
Population	Adolescents aged ≥12 years and adults from clinical and non-clinical populations
Exposure	Emotional expressive flexibility, including overall expressive flexibility and the constituent abilities to enhance and suppress emotional expression, assessed using the Expressive Regulation Paradigm, the Flexible Regulation of Emotional Expression (FREE) Scale, or a comparable operationalization
Outcome	Psychopathology, psychological distress, psychological well-being, resilience, adaptive functioning, or social and interpersonal functioning

**Table 3:** Studies excluded after full-text assessment and reasons for exclusion.

Study	Identification Route	Reason for Exclusion
Lass-Hennemann et al. [49]	Database searching	Wrong population
Emery and Hess [50]	Expert consultation	Association of interest not examined

**Table 4:** Study and sample characteristics of the included studies (alphabetical by author).

Study	Country	Design of the Included Analyses	Population and Setting	Sample Size (*n*)	Age, *M* (*SD*); Range	Female, *n* (%)	Expressive-Flexibility Measure
Ang and Tsai [45]	USA	Cross-sectional/longitudinal	Non-clinical: first-year college students	279	17.92 (NR); NR	196 (70.3%)	FREE
Ang and Tsai [51]	USA	Cross-sectional	Non-clinical: community adults recruited via MTurk	610	45.79 (17.02); 18–65+	270 (44.3%)	FREE
Bonanno et al. [25]	USA	Longitudinal	Non-clinical: New York City undergraduates assessed before and after the September 11 attacks	87	18.05 (0.59); NR	NR	ERP
Burton and Bonanno, Study 1 [26]	USA	Cross-sectional	Non-clinical: community adults recruited online	200	26.52 (5.09); 18–40	122 (61.0%)	FREE
Chen et al. [32]	China	Cross-sectional	Non-clinical: Chinese undergraduate students	310	20.03 (1.35); 18–24	146 (47.1%)	FREE, Chinese version
Dwyer et al. [38]	USA	Cross-sectional	Clinical: community adults recruited via MTurk with harmful alcohol use (AUDIT ≥ 16)	200	37.30 (11.41); 22–69	94 (47.0%)	FREE
Gonzalez-Escamilla et al. [33]	Germany	Cross-sectional	Non-clinical: young adults, predominantly university students	222	23.36 (5.05); NR	173 (77.9%)	FREE, German version
Gupta and Bonanno [36]	USA	Cross-sectional	Mixed: bereaved adults with or without complicated grief and married controls	118	49.50 (9.80); NR	79 (66.9%)	ERP
Lenzo et al. [52]	Italy	Cross-sectional	Non-clinical: palliative home-care practitioners	65	36.49 (9.94); 22–63	34 (52.3%)	FREE, Italian version
Lenzo et al. [53]	Italy	Cross-sectional	Non-clinical: healthcare workers	214	39.80 (11.39); 23–72	130 (60.7%)	FREE, Italian version
Levy-Gigi et al. [54]	Israel	Cross-sectional	Non-clinical: active-duty firefighters	82	33.59 (9.56); 25–66	0 (0.0%)	FREE
Maccallum et al. [55]	Australia	Cross-sectional	Non-clinical: undergraduate students	84	19.42 (2.54); NR	60 (71.4%)	ERP
Nardelli et al. [56]	France	Cross-sectional	Non-clinical: French-speaking adults recruited online	397	34.40 (14.70); 18–73	321 (80.9%)	FREE, French version
Pitur and Miu [57]	Romania	Cross-sectional	Non-clinical: Romanian undergraduate students	62	20.27 (2.76); 18–36	55 (88.7%)	ERP
Quattropani et al. [58]	Italy	Cross-sectional	Non-clinical: Italian community adults recruited online	503	28.65 (9.26); 21–72	428 (85.1%)	FREE, Italian version
Rodin et al. [37]	USA	Cross-sectional	Mixed: combat veterans with and without PTSD	59	33.60 (6.60); 23–52	1 (1.7%)	ERP
Sardella et al. [59]	Italy	Cross-sectional/longitudinal	Clinical: older adults attending a geriatric outpatient service	141 at baseline; 104 at follow-up	80.26 (6.39); NR	71 (68.3%)	FREE, Italian version
Shangguan et al. [34]	China	Cross-sectional	Non-clinical: Chinese college students	711	20.98 (2.28); 17–33	396 (55.7%)	FREE, Chinese version
Shangguan et al. [60]	China	Cross-sectional	Non-clinical: Chinese college students	1122	19.97 (2.25); 18–33	542 (48.3%)	FREE, Chinese version
Southward and Cheavens [28]	USA	Cross-sectional/longitudinal	Non-clinical: undergraduate students	138	20.01 (2.27); NR	NR	ERP
Strickland and Skolnick [46]	India, USA	Cross-sectional	Non-clinical: community adults recruited via MTurk	360	35.07 (10.41); 19–70	155 (43.1%)	FREE
Wang et al., Experiment 1 [43]	China	Experimental	Non-clinical: Chinese secondary-school students	147	13.27 (0.38); 12.42–14.33	74 (50,3%)	ERP
Wang et al. [61]	USA	Cross-sectional/longitudinal	Non-clinical: cisgender sexual minority men	377	29.64 (13.46); 18–68	0 (0.0%)	FREE
Wang et al. [62]	China	Cross-sectional/longitudinal	Non-clinical: Chinese adolescents	274	13.56 (0.63); 12–15	138 (50.4%)	CAFE
Wang et al. [63]	USA	Cross-sectional	Clinical: adults with major depressive disorder and recent heavy alcohol or drug use	112	33.16 (12.07); 18–68	66 (58.9%)	FREE
Westphal et al. [29]	USA	Cross-sectional	Non-clinical: undergraduates from an earlier expressive-flexibility cohort	50	NR	32 (64.0%)	ERP
Zhang et al. [64]	China	Cross-sectional	Non-clinical: Chinese high school students	766	14.96 (2.04); 12–18	400 (52.2%)	AFREE
Zhao and Wang [65]	China	Cross-sectional	Non-clinical: Chinese college students	607	19.02 (1.02); 16–23	425 (70.0%)	FREE, Chinese version
Zhu and Bonanno [39]	USA	Cross-sectional	Non-clinical: community adults recruited through advertisements and public notices	97	34.39 (11.52); 19–62	48 (49.5%)	ERP

*Note*: Design classifications refer to the analyses included in this review rather than necessarily to the overall design of the original publication. Multiple classifications are listed where a study contributes more than one type of analysis. Critical appraisal tools were selected according to the design of each included contribution. Sample sizes and demographic characteristics refer to the analytic sample relevant to the extracted association whenever available. For Sardella et al. [59], baseline and follow-up sample sizes are reported separately; age and sex distribution refer to the follow-up sample. Female percentages were calculated using the total analytic sample of the respective study. Sex and gender information reflects the categories and terminology available in the original reports. Where exact sex or gender counts were unavailable, approximate counts were derived from reported percentages and may be affected by rounding. Bonanno et al. [25] and Westphal et al. [29] used partially overlapping samples. AFREE = Adolescent Flexible Regulation of Emotional Expression Scale; AUDIT = Alcohol Use Disorders Identification Test; CAFE = Child and Adolescent Flexible Expressiveness Scale; ERP = Expressive Regulation Paradigm; FREE = Flexible Regulation of Emotional Expression Scale; M = mean; MTurk = Amazon Mechanical Turk; NR = not reported; SD = standard deviation.

**Table 5:** Overview of Mental Health Outcome Domains and Principal Findings.

Outcome Domain	Publications (*n*)	Outcomes Examined	Principal Findings
Psychopathology	22	Depressive, anxiety-related, stress-related, and trauma-related symptoms; psychological distress; complicated grief; borderline symptoms; drug-related problems	Higher overall expressive flexibility was generally associated with lower psychopathological symptom burden. Component-level, prospective, and conditional findings were more variable.
Psychological well-being	7	Life satisfaction; positive affect; general well-being; psychological health-related quality of life	Higher overall expressive flexibility was generally associated with greater psychological well-being. Component-specific and prospective associations varied across outcomes and population groups.
Resilience and adaptive functioning	9	Hope; resilience; ego-resilience; long-term adjustment; burnout; physical health-related quality of life	Higher expressive flexibility was associated with greater psychological resources and better adjustment under stress. Associations varied by context, with burnout and physical health-related quality of life showing outcome- and component-specific findings.
Social functioning	6	Perceived social support; social functioning deficits; peer acceptance; friendship quality; relationship stress	Higher expressive flexibility was generally associated with more favorable social functioning, although some associations were absent or reversed under specific individual and interpersonal conditions.

*Note*: Publications could contribute to more than one outcome domain; consequently, the numbers presented across domains do not sum to the 29 publications included in the systematic review.

## Data Availability

Materials related to this systematic review are publicly available via the Open Science Framework (OSF): https://osf.io/cwt7g/ (accessed on 26 September 2026). These materials comprise a README file, an Excel workbook containing study characteristics and outcome-level extraction data, and three R scripts reproducing the meta-analyses of depressive symptoms, life satisfaction, and anxiety. No individual-level or personally identifiable data are included.

## Supplementary Materials

The following supporting information can be downloaded at: https://www.mdpi.com/article/10.3390/brainsci16101041/s1. The materials are provided in four separate files: (S1) “JBI critical appraisal of expressive flexibility studies,” containing the appraisal tables (Table S1: Analytical cross sectional contributions; Table S2: Cohort contributions; Table S3: Randomized experimental contribution), checklist questions, application rules, and item-level judgments with supporting rationales and source locations; (S2) the completed PRISMA 2020 checklist; and (S3) supplementary figures containing Figure S1: Funnel Plot for the Association Between Overall Expressive Flexibility and Depressive Symptoms, and Figure S2: Funnel Plot for the Association Between Overall Expressive Flexibility and Anxiety; and (S4) “Outcome-level appraisal of the evidence,” containing the assessment rules, quantitative overview, and outcome-specific appraisals with supporting evidence and limitations.

## References

[1] Pessoa L. (2008). On the relationship between emotion and cognition. Nat. Rev. Neurosci..

[2] George J.M., Dane E. (2016). Affect, emotion, and decision making. Organ. Behav. Hum. Decis. Process.

[3] van Kleef G.A., Côté S. (2022). The Social Effects of Emotions. Annu. Rev. Psychol..

[4] Kret M.E., Ploeger A. (2015). Emotion processing deficits: A liability spectrum providing insight into comorbidity of mental disorders. Neurosci. Biobehav. Rev..

[5] McTeague L.M., Rosenberg B.M., Lopez J.W., Carreon D.M., Huemer J., Jiang Y., Chick C.F., Eickhoff S.B., Etkin A. (2020). Identification of Common Neural Circuit Disruptions in Emotional Processing Across Psychiatric Disorders. Am. J. Psychiatry.

[6] Eilert D.W. (2025). Differenzielle Muster der Emotionsverarbeitung: Die Rolle von Bindungsrepräsentationen und Psychopathologie. Doctoral Thesis.

[7] Eilert D.W., Mensah P., Buchheim A. (2026). The Dual Facets of Emotion Perception in Adult Attachment Representations: A Systematic Review on Impathy and Empathy. Brain Sci..

[8] Neubrand S., Gaab J. (2022). The missing construct: Impathy. Front. Psychol..

[9] Gross J.J., Lewis M., Haviland-Jones J.M., Feldman Barrett L. (2008). Emotion Regulation. Handbook of Emotions.

[10] Price T.F., Harmon-Jones E. (2015). Embodied emotion: The influence of manipulated facial and bodily states on emotive responses. WIREs Cogn. Sci..

[11] Coles N.A., Larsen J.T., Lench H.C. (2019). A meta-analysis of the facial feedback literature: Effects of facial feedback on emotional experience are small and variable. Psychol. Bull..

[12] Elkjaer E., Mikkelsen M.B., Michalak J., Mennin D.S., O’Toole M.S. (2022). Expansive and Contractive Postures and Movement: A Systematic Review and Meta-Analysis of the Effect of Motor Displays on Affective and Behavioral Responses. Perspect. Psychol. Sci..

[13] Aucouturier J.-J., Johansson P., Hall L., Segnini R., Mercadié L., Watanabe K. (2016). Covert digital manipulation of vocal emotion alter speakers’ emotional states in a congruent direction. Proc. Natl. Acad. Sci. USA.

[14] Scherer K.R. (2009). The dynamic architecture of emotion: Evidence for the component process model. Cogn. Emot..

[15] Wainio-Theberge S., Armony J. (2026). Manipulating posture implicitly through environmental constraints influences mood and risk-taking behaviour. Br. J. Psychol..

[16] Keltner D., Sauter D., Tracy J., Cowen A. (2019). Emotional Expression: Advances in Basic Emotion Theory. J. Nonverbal Behav..

[17] Cowen A., Sauter D., Tracy J.L., Keltner D. (2019). Mapping the Passions: Toward a High-Dimensional Taxonomy of Emotional Experience and Expression. Psychol. Sci. Public. Interest..

[18] Cordaro D.T., Sun R., Kamble S., Hodder N., Monroy M., Cowen A., Bai Y., Keltner D. (2019). The recognition of 18 facial-bodily expressions across nine cultures. Emotion.

[19] Bakker A.B., Heuven E. (2006). Emotional dissonance, burnout, and in-role performance among nurses and police officers. Int. J. Stress. Manag..

[20] Heuven E., Bakker A. (2003). Emotional dissonance and burnout among cabin attendants. Eur. J. Work. Organ. Psychol..

[21] Winkler A.D., Zapf D., Kern M. (2023). Effects of emotion-rule dissonance on emotional exhaustion and physiological health: A two-wave study. Appl. Psychol..

[22] Zapf D., Vogt C., Seifert C., Mertini H., Isic A. (1999). Emotion Work as a Source of Stress: The Concept and Development of an Instrument. Eur. J. Work. Organ. Psychol..

[23] Nielsen M.B., Johannessen H.A., Christensen J.O., Finne L.B. (2023). Emotional dissonance and burnout among child welfare workers: The moderating role of social support from colleagues, supervisors, and organization. J. Soc. Work..

[24] Jeon M.-K., Yoon H., Yang Y. (2022). Emotional Dissonance, Job Stress, and Intrinsic Motivation of Married Women Working in Call Centers: The Roles of Work Overload and Work-Family Conflict. Adm. Sci..

[25] Bonanno G.A., Papa A., Lalande K., Westphal M., Coifman K. (2004). The Importance of Being Flexible: The Ability to Both Enhance and Suppress Emotional Expression Predicts Long-Term Adjustment. Psychol. Sci..

[26] Burton C.L., Bonanno G.A. (2016). Measuring ability to enhance and suppress emotional expression: The Flexible Regulation of Emotional Expression (FREE) Scale. Psychol. Assess..

[27] Bonanno G.A., Burton C.L. (2013). Regulatory Flexibility: An Individual Differences Perspective on Coping and Emotion Regulation. Perspect. Psychol. Sci..

[28] Southward M.W., Cheavens J.S. (2017). Assessing the Relation Between Flexibility in Emotional Expression and Symptoms of Anxiety and Depression: The Roles of Context Sensitivity and Feedback Sensitivity. J. Soc. Clin. Psychol..

[29] Westphal M., Seivert N.H., Bonanno G.A. (2010). Expressive flexibility. Emotion.

[30] Greenspoon P.J., Saklofske D.H. (2001). Toward an Integration of Subjective Well-Being and Psychopathology. Soc. Indic. Res..

[31] Keyes C.L. (2005). Mental illness and/or mental health? Investigating axioms of the complete state model of health. J. Consult. Clin. Psychol..

[32] Chen S., Chen T., Bonanno G.A. (2018). Expressive flexibility: Enhancement and suppression abilities differentially predict life satisfaction and psychopathology symptoms. Pers. Individ. Dif..

[33] Gonzalez-Escamilla G., Dörfel D., Becke M., Trefz J., Bonanno G.A., Groppa S. (2022). Associating Flexible Regulation of Emotional Expression with Psychopathological Symptoms. Front. Behav. Neurosci..

[34] Shangguan C., Zhang L., Wang Y., Wang W., Shan M., Liu F. (2022). Expressive Flexibility and Mental Health: The Mediating Role of Social Support and Gender Differences. Int. J. Environ. Res. Public. Health.

[35] Page M.J., McKenzie J.E., Bossuyt P.M., Boutron I., Hoffmann T.C., Mulrow C.D., Shamseer L., Tetzlaff J.M., Akl E.A., Brennan S.E. (2021). The PRISMA 2020 statement: An updated guideline for reporting systematic reviews. BMJ.

[36] Gupta S., Bonanno G.A. (2011). Complicated grief and deficits in emotional expressive flexibility. J. Abnorm. Psychol..

[37] Rodin R., Bonanno G.A., Rahman N., Kouri N.A., Bryant R.A., Marmar C.R., Brown A.D. (2017). Expressive flexibility in combat veterans with posttraumatic stress disorder and depression. J. Affect. Disord..

[38] Dwyer C.L., Craft W.H., Tomlinson D.C., Tegge A.N., Kim-Spoon J., Bickel W.K. (2024). Latent profiles of regulatory flexibility in alcohol use disorder: Associations with delay discounting and symptoms of depression, anxiety, and stress. Alcohol. Clin. Exp. Res..

[39] Zhu Z., Bonanno G.A. (2017). Affective Flexibility: Relations to Expressive Flexibility, Feedback, and Depression. Clin. Psychol. Sci..

[40] Barker T.H., Hasanoff S., Aromataris E., Stone J.C., Leonardi-Bee J., Sears K., Klugar M., Tufanaru C., Moola S., Liu X.-L. (2026). The revised JBI critical appraisal tool for the assessment of risk of bias for analytical cross-sectional studies. JBI Evid. Synth..

[41] Barker T.H., Hasanoff S., Aromataris E., Stone J.C., Leonardi-Bee J., Sears K., Habibi N., Klugar M., Tufanaru C., Moola S. (2025). The revised JBI critical appraisal tool for the assessment of risk of bias for cohort studies. JBI Evid. Synth..

[42] Barker T.H., Stone J.C., Sears K., Klugar M., Tufanaru C., Leonardi-Bee J., Aromataris E., Munn Z. (2023). The revised JBI critical appraisal tool for the assessment of risk of bias for randomized controlled trials. JBI Evid. Synth..

[43] Wang Y., Hawk S.T., Zong W. (2020). Bidirectional effects between expressive regulatory abilities and peer acceptance among Chinese adolescents. J. Exp. Child. Psychol..

[44] Guyatt G.H., Oxman A.D., Vist G.E., Kunz R., Falck-Ytter Y., Alonso-Coello P., Schünemann H.J. (2008). GRADE: An emerging consensus on rating quality of evidence and strength of recommendations. BMJ.

[45] Ang J.Y.Z., Tsai W. (2023). Cultural differences in the relations between expressive flexibility and life satisfaction over time. Front. Psychol..

[46] Strickland M.G., Skolnick A.J. (2020). Expressive flexibility and trait anxiety in India and the United States. Pers. Individ. Dif..

[47] R Core Team (2026). R: A Language and Environment for Statistical Computing, 4.5.3.

[48] Viechtbauer W. (2010). Conducting Meta-Analyses in R with the metafor Package. J. Stat. Softw..

[49] Lass-Hennemann J., Sopp M.R., Ruf N., Equit M., Schafer S.K., Wirth B.E., Michael T. (2024). Generation climate crisis, COVID-19, and Russia-Ukraine-War: Global crises and mental health in adolescents. Eur. Child. Adolesc. Psychiatry.

[50] Emery L., Hess T.M. (2011). Cognitive consequences of expressive regulation in older adults. Psychol. Aging.

[51] Ang J.Y.Z., Tsai W. (2025). Age and Gender Effects on the Relationship Between Expressive Flexibility and Mental Health. J. Adult Dev..

[52] Lenzo V., Bordino V., Bonanno G.A., Quattropani M.C. (2020). Understanding the role of regulatory flexibility and context sensitivity in preventing burnout in a palliative home care team. PLoS ONE.

[53] Lenzo V., Quattropani M.C., Sardella A., Martino G., Bonanno G.A. (2021). Depression, Anxiety, and Stress Among Healthcare Workers During the COVID-19 Outbreak and Relationships with Expressive Flexibility and Context Sensitivity. Front. Psychol..

[54] Levy-Gigi E., Donner R., Bonanno G.A. (2020). Free Your Mind: Emotional Expressive Flexibility Moderates the Effect of Stress on Post-Traumatic Stress Disorder Symptoms. Int. J. Mol. Sci..

[55] Maccallum F., Tran S., Bonanno G.A. (2021). Expressive flexibility and anxiety. J. Affect. Disord..

[56] Nardelli C., Paucsik M., Weiner L., Bonanno G.A., Bortolon C. (2024). Validation of the French Versions of the Context-Sensitivity Index (CSI) and the Flexible Regulation of Emotional Expression Scale (FREE). J. Psychopathol. Behav. Assess..

[57] Pitur S., Miu A.C. (2020). Childhood maltreatment and expressive flexibility: Specific effects of threat and deprivation?. Cogn. Emot..

[58] Quattropani M.C., Lenzo V., Sardella A., Bonanno G.A. (2022). Expressive flexibility and health-related quality of life: The predictive role of enhancement and suppression abilities and relationships with trait emotional intelligence. Scand. J. Psychol..

[59] Sardella A., Lenzo V., Bonanno G.A., Basile G., Quattropani M.C. (2021). Expressive Flexibility and Dispositional Optimism Contribute to the Elderly’s Resilience and Health-Related Quality of Life during the COVID-19 Pandemic. Int. J. Environ. Res. Public. Health.

[60] Shangguan C., Wang Y., Wang X., Zhang L., Zhou B. (2024). The more anxious, the less flexible? Association of trait anxiety with expressive flexibility and the mediating role of emotion regulation strategies. Curr. Psychol..

[61] Wang K., Maiolatesi A.J., Burton C.L., Scheer J.R., Pachankis J.E. (2022). Emotion Regulation in Context: Expressive Flexibility as a Stigma Coping Resource for Sexual Minority Men. Psychol. Sex. Orientat. Gend. Divers..

[62] Wang Y., Hawk S.T., Branje S., Van Lissa C.J. (2023). Longitudinal links between expressive flexibility and friendship quality in adolescence: The moderating effect of social anxiety. J. Adolesc..

[63] Wang K., Girard R., Manning R.B., Weiss N.H. (2026). Expressive Flexibility as a Buffer in the Association Between Substance Use Stigma and Drug Use Problems Among Substance-Using Individuals with Depression. Stigma Health.

[64] Zhang S., Liu J., Sang B., Zhao Y. (2023). Age and gender differences in expressive flexibility and the association with depressive symptoms in adolescents. Front. Psychol..

[65] Zhao Y., Wang P. (2024). Flexible Regulation of Positive and Negative Emotion Expression: Reexamining the Factor Structure of the Flexible Regulation of Emotional Expression Scale (FREE) Based on Emotion Valence. J. Intell..

[66] Aldao A. (2013). The Future of Emotion Regulation Research: Capturing Context. Perspect. Psychol. Sci..

[67] Aldao A., Sheppes G., Gross J.J. (2015). Emotion Regulation Flexibility. Cogn. Ther. Res..

[68] Aldao A., Nolen-Hoeksema S., Schweizer S. (2010). Emotion-regulation strategies across psychopathology: A meta-analytic review. Clin. Psychol. Rev..

[69] Bonanno G.A. (2021). The resilience paradox. Eur. J. Psychotraumatol.

[70] Chen S., Bonanno G.A. (2021). Components of Emotion Regulation Flexibility: Linking Latent Profiles to Depressive and Anxious Symptoms. Clin. Psychol. Sci..

[71] Robinson M., McGlinchey E., Bonanno G.A., Spikol E., Armour C. (2022). A path to post-trauma resilience: A mediation model of the flexibility sequence. Eur. J. Psychotraumatol..

[72] Chen M.S., Bi K., Han X., Sun P., Bonanno G.A. (2024). Emotion regulation flexibility and momentary affect in two cultures. Nat. Ment. Health.

[73] Hilberdink C.E., Zhao Y., McKernan S., Gershov S., Mueller V., Wall S.P., Schultebraucks K. (2026). Bouncing back from stress: Objective markers of expressive flexibility and resilience in emergency healthcare workers using computer vision. NPP Digit. Psychiatry Neurosci..

[74] Shapiro F. (2018). Eye Movement Desensitization and Reprocessing (EMDR) Therapy: Basic Principles, Protocols, and Procedures.

[75] Eilert D.W. (2021). Integratives Emotionscoaching mit emTrace: Wie emotionale Veränderung Wirklich Gelingt.

[76] Eilert D.W., Mensah P., Schmäh M. (2025). Emotion-focused ego-state coaching reduces communication apprehension: Evidence from a randomized controlled study. Front. Organ. Psychol..

[77] Moss D. (2020). Physiological Monitoring to Enhance Clinical Hypnosis and Psychotherapy. Int. J. Clin. Exp. Hypn..

[78] Eilert D.W. (2020). Körpersprache Entschlüsseln & Verstehen: Die Mimikresonanz-Profibox.

[79] Blanch-Hartigan D. (2012). An effective training to increase accurate recognition of patient emotion cues. Patient Educ. Couns..

[80] Friesen W.V. (1972). Cultural Differences in Facial Expressions in A Social Situation: An Experimental Test of the Concept of Display Rules. Doctoral Dissertation.

[81] Matsumoto D., Yoo S.H., Hirayama S., Petrova G. (2005). Development and validation of a measure of display rule knowledge: The display rule assessment inventory. Emotion.

[82] Matsumoto D., Seung Hee Y., Fontaine J. (2008). Mapping Expressive Differences Around the World. J. Cross-Cult. Psychol..

[83] Eilert D.W., Buchheim A. (2023). Attachment-Related Differences in Emotion Regulation in Adults: A Systematic Review on Attachment Representations. Brain Sci..

[84] Hopf A.G.M., Buchheim A., Hopf M., Cajkovsky M., Eilert D.W. (2025). A Psychological Guide to Upper Face Botulinum Toxin Injections: Baseline Emotional Functions of Facial Expressions. J. Cosmet. Dermatol..

[85] Hopf A.G.M., Buchheim A., Hopf M., Eilert D.W. (2026). A Psychological Guide to Lower Face Botulinum Toxin Injections: Baseline Emotional Functions of Facial Expressions. J. Cosmet. Dermatol..

[86] Eilert D.W., Hopf A.G.M., Alfertshofer M., Prinz V., Friedrich M., Cajkovsky M. (2026). Botulinum Toxin and Social Perception: Effects of Upper-Face Treatment on Facial Emotion Attribution. Aesthet. Surg. J..

