# Supplementary material for: Emotional Expressive Flexibility and Mental Health: A Systematic Review and Meta-Analysis
Source: Brain Sci. 2026 Sep 29;16(10):1041. doi: 10.3390/brainsci16101041 (PMC13702306; doi:10.3390/brainsci16101041)
Supplement: Supplementary file 1 [file brainsci-16-01041-s001.zip › Supplementary_File_S1_JBI_critical_appraisal_Review_ExpressiveFlexibility.pdf]

JBI critical appraisal of expressive flexibility studies

Supplementary material for the systematic review of expressive flexibility

This supplement reports item-level appraisals for all 29 included studies. It distinguishes concurrent observational associations, longitudinal contributions and the randomized peer-acceptance experiment. The three overview tables provide the judgments; the supporting appraisal sheets explain the evidence and any unresolved limitations.

How to read the supplement

Section	Contents
S1	Analytical cross-sectional contributions: 27 study appraisals, including baseline/concurrent results from mixed designs.
S2	Cohort contributions: 6 study appraisals. Wang et al. (2022) has a supplementary longitudinal model but no baseline EF measurement.
S3	Randomized experimental contribution: Wang et al. (2020), Experiment 1, assessing peer acceptance of a target displaying expressive ability.
S4	Original item wording, levels of appraisal and tool references.
S5	Supporting judgments for each study, in extraction-table order; mixed studies have a separate sheet for each relevant design.

There are 34 design-specific appraisals across 29 unique studies. These are not 34 independent samples. Studies are identified by author and year, consistently across the overview tables and supporting sheets. These labels are independent of reference numbering in the main manuscript. Study or experiment numbers distinguish specific contributions where applicable.

Judgment labels

Yes: the reported information supports the criterion. No: a relevant safeguard is absent, or required reporting detail is not supplied. Unclear: a specific unresolved information gap prevents a defensible judgment. N/A: the criterion does not apply to the assessed estimand or design; the reason is documented. No summed scores, numerical cutoffs or overall high/moderate/low categories are calculated.

Outcome and result specificity

Outcomes are grouped only when their item judgments are identical. Separate rows show differing judgments. For analytical cross-sectional contributions, Q6 refers to the confounding-control strategy used in the reviewed analyses for the stated outcome. Where both adjusted models and unadjusted correlations are included, a “Yes” judgment applies to the adjusted analysis only; it does not imply that the separately reported correlations are adjusted. Where the reviewed association is unadjusted and no confounding-control strategy applies to it, Q6 is rated “No”. Adjustment in analyses outside the reviewed outcome or contribution does not justify a “Yes” judgment. The supporting sheets identify this distinction explicitly. Selection, measurement, confounding, retention and statistical limitations should be interpreted by domain rather than counted.

Application rules

Design assignment. The appraisal follows the contribution extracted for this review. An expressive laboratory task is an exposure measure unless the reviewed outcome is a consequence of randomized assignment. Thus, observational symptom associations embedded in laboratory experiments receive a cross-sectional appraisal. Mixed concurrent/longitudinal contributions receive both tools.

Eligibility and setting. Achieved sample characteristics do not substitute for explicit eligibility criteria. A stated broad/no-exclusion recruitment policy may satisfy Q1. The cross-sectional Q8 is a reporting item; a No is not proof of biased recruitment. A missing calendar date alone does not determine Q8 when the recruitment frame and context are otherwise adequately described.

Condition criteria. Cross-sectional Q2 addresses condition-based selection or classification. It is N/A for unselected continuous symptom/well-being outcomes without a case definition. Explicit health-related eligibility with unclear ascertainment is flagged. Screening thresholds are not described as confirmed diagnoses.

Measurement. Self-report can be appropriate for perceived ability and subjective symptoms. Judgments consider the particular version, language, scoring and available validity/reliability evidence. Missing alpha alone is not an automatic No/Unclear; undocumented adaptation or conflicting scoring can be. Minor, justified modifications may remain adequate for continuous scores, including the 20-item BDI-II score used by Chen et al.

Confounding. Relevant covariates and their actual use are distinguished from focal predictors/mediators. A Yes does not exclude residual confounding. Adjustment in a profile analysis or another outcome model is not attributed to an extracted unadjusted association. An interaction term alone is not a confounding-control strategy. Q5 is judged at study level: it records whether the study identifies potential confounders for any analysis. Whether they are applied to the association extracted for this review is judged under Q6.

Statistical analysis. The method must match the question and data. Missing diagnostic reporting alone is not an automatic negative judgment, and unreported checks are never assumed to have occurred. A specific unresolved concern, such as unmodeled school clustering, is retained. Atemporal regression or mediation does not establish temporal or causal direction.

Continuous cohort exposure. For cohorts analyzing EF continuously within one recruited sample, the two-group selection comparison in cohort Q1 is N/A. Q2 assesses whether the same exposure protocol was applied across participants. These are review-specific applicability decisions, not alterations to the original question wording.

Continuous cohort outcome. Cohort Q6 is N/A for continuous symptom/well-being level or change rather than incident disease. Temporal order and baseline-outcome adjustment are nevertheless stated for each study. N/A never means that temporal precedence has been demonstrated; Wang et al. (2022) is specifically limited in this respect.

Retention. Reasons, extent and exploration of attrition are assessed separately from strategies to address it. Complete-case analysis and a nonsignificant dropout comparison are not by themselves corrections for missing outcomes. Routine scheduled assessment alone is not treated as an additional retention safeguard. No fixed attrition percentage substitutes for examining the reported circumstances.

Table S1 Analytical cross sectional contributions

JBI analytical cross-sectional tool (2026), Q1–Q8. Entries are outcome-level summaries; details and qualifications appear in S5.

Study	Outcomes assessed	Q1	Q2	Q3	Q4	Q5	Q6	Q7	Q8
Ang and Tsai (2023)	Baseline depression and life satisfaction	Yes	N/A	Yes	Yes	Yes	No	Yes	Yes
Ang and Tsai (2025)	Life satisfaction, positive affect, hope and received social support	Yes	N/A	Yes	Yes	Yes	Yes	Yes	Yes
Burton and Bonanno (2016) Study 1	Ego resilience and social functioning deficits	Yes	N/A	Yes	Yes	No	No	Yes	Yes
Burton and Bonanno (2016) Study 1	Depressive symptoms	Yes	N/A	Yes	Unclear	No	No	Yes	Yes
Chen et al. (2018)	Depression, anxiety and life satisfaction	No	N/A	Yes	Yes	Yes	Yes	Yes	No
Chen et al. (2018)	Resilience	No	N/A	Yes	Yes	Yes	No	Yes	No
Dwyer et al. (2024)	Depression, anxiety and stress	Yes	Yes	Yes	Yes	Yes	No	Yes	Yes
Gonzalez-Escamilla et al. (2022)	Depression, anxiety, stress, well-being and resilience	Yes	N/A	Yes	Yes	Yes	Yes	Yes	Yes
Gupta and Bonanno (2011)	Complicated grief group comparisons	Yes	Yes	Yes	Yes	Yes	Yes	Yes	Yes
Lenzo et al. (2020)	Emotional exhaustion, depersonalization and personal accomplishment	Yes	Unclear	Unclear	Yes	Yes	Yes	Unclear	Yes
Lenzo et al. (2021)	Depression, anxiety and stress	Yes	N/A	Unclear	Yes	Yes	Yes	Yes	Yes

Note. N/A = not applicable. The questions are reproduced in S4. A Yes at Q6 applies to the stated outcome-level adjustment strategy; any separate zero-order correlation remains unadjusted. Q8 concerns reporting of subjects and setting. No overall score is calculated.

Table S1 Analytical cross sectional contributions continued

JBI analytical cross-sectional tool (2026), Q1–Q8. Entries are outcome-level summaries; details and qualifications appear in S5.

Study	Outcomes assessed	Q1	Q2	Q3	Q4	Q5	Q6	Q7	Q8
Levy-Gigi et al. (2020)	PTSD symptoms	Yes	Unclear	Unclear	Unclear	No	No	Yes	Yes
Maccallum et al. (2021)	Anxiety, depression, stress and trait-anxiety group comparisons	Unclear	Unclear	Yes	Yes	Yes	No	Yes	Yes
Nardelli et al. (2024)	Depression, anxiety, stress, total distress and borderline symptoms	Yes	N/A	Yes	Yes	No	No	Yes	Yes
Pițur and Miu (2020)	Depression and trait anxiety	No	N/A	Yes	Yes	Yes	No	Yes	No
Quattropani et al. (2022)	Physical and psychological health-related quality of life	No	N/A	Yes	Yes	Yes	Yes	Yes	No
Rodin et al. (2017)	PTSD severity and PTSD/depression screening groups	Yes	Yes	Yes	Yes	Yes	Yes	Yes	Yes
Rodin et al. (2017)	Depressive symptom severity	Yes	Yes	Yes	Yes	Yes	No	Yes	Yes
Sardella et al. (2021)	Baseline physical health-related quality of life	Yes	Yes	Unclear	Yes	Yes	Yes	Yes	Yes
Sardella et al. (2021)	Baseline psychological health-related quality of life	Yes	Yes	Unclear	Yes	Yes	No	Yes	Yes
Shangguan et al. (2022)	Depression and life satisfaction	No	N/A	Yes	Yes	Yes	Yes	Yes	No
Shangguan et al. (2022)	Perceived social support	No	N/A	Yes	Yes	Yes	No	Yes	No
Shangguan et al. (2024)	Trait anxiety	Yes	Unclear	Yes	Yes	No	No	Yes	Yes

Note. N/A = not applicable. The questions are reproduced in S4. A Yes at Q6 applies to the stated outcome-level adjustment strategy; any separate zero-order correlation remains unadjusted. Q8 concerns reporting of subjects and setting. No overall score is calculated.

Table S1 Analytical cross sectional contributions continued

JBI analytical cross-sectional tool (2026), Q1–Q8. Entries are outcome-level summaries; details and qualifications appear in S5.

Study	Outcomes assessed	Q1	Q2	Q3	Q4	Q5	Q6	Q7	Q8
Southward and Cheavens (2017)	Baseline depression and anxiety	Yes	N/A	Yes	Yes	Yes	No	Yes	Yes
Strickland and Skolnick (2020)	Trait anxiety	Yes	N/A	Yes	Yes	Yes	Yes	Yes	Yes
Wang et al. (2022)	Psychological distress at T2	Yes	N/A	Yes	Yes	Yes	Yes	Yes	Yes
Wang et al. (2023)	Concurrent friendship quality and social anxiety	Yes	N/A	Yes	Yes	Yes	No	Unclear	Yes
Wang et al. (2026)	Drug-related problems	Yes	Yes	Yes	Yes	Yes	Yes	Yes	Yes
Westphal et al. (2010)	Friend-rated psychological adjustment	No	N/A	Yes	Yes	Yes	Yes	Yes	No
Zhang et al. (2023)	Depressive symptoms	No	N/A	Yes	Yes	Yes	Yes	Unclear	Yes
Zhao and Wang (2024)	Depression, anxiety, stress and relationship stress	No	N/A	Unclear	Yes	Yes	Yes	Yes	Yes
Zhu and Bonanno (2017)	Baseline depression in relation to within-session EF change	Yes	N/A	Yes	Yes	Yes	Yes	Yes	Yes

Note. N/A = not applicable. The questions are reproduced in S4. A Yes at Q6 applies to the stated outcome-level adjustment strategy; any separate zero-order correlation remains unadjusted. Q8 concerns reporting of subjects and setting. No overall score is calculated.

Table S2 Cohort contributions

JBI cohort tool (2025), Q1–Q11. These six contributions arise from six of the 29 included studies; five also have concurrent/baseline results in S1.

Study	Outcomes assessed	Q1	Q2	Q3	Q4	Q5	Q6	Q7	Q8	Q9	Q10	Q11
Ang and Tsai (2023)	Later depression and life satisfaction	N/A	Yes	Yes	Yes	Yes	N/A	Yes	Yes	No	No	Yes
Bonanno et al. (2004)	Psychological distress at follow-up	N/A	Yes	Yes	Yes	Yes	N/A	Unclear	Yes	Unclear	No	Yes
Sardella et al. (2021)	Follow-up physical and mental health-related quality of life	N/A	Yes	Unclear	Yes	Yes	N/A	Yes	Yes	Unclear	No	Yes
Southward and Cheavens (2017)	Later anxiety/depression composite	N/A	Yes	Yes	Yes	Yes	N/A	Yes	Yes	Unclear	No	Yes
Wang et al. (2022)	Distress at T2 in the supplementary longitudinal model	N/A	Yes	Yes	Yes	Yes	N/A	Yes	Unclear	Unclear	No	Yes
Wang et al. (2023)	Later friendship quality and social anxiety	N/A	Yes	Yes	Yes	Yes	N/A	Yes	Yes	Yes	No	Unclear

Note. Q1 and Q6 are N/A under the stated continuous-exposure/continuous-outcome application rules. This does not endorse temporal precedence or absence of baseline symptoms. A Yes at Q5 does not mean that baseline levels of the same outcome were always controlled. See the specific models below.

Timing and adjustment distinctions

Study	Interpretation of the longitudinal contribution
Ang and Tsai (2023)	T1 EF precedes week-13 outcomes; the baseline value of the same outcome is controlled.
Bonanno et al. (2004)	EF precedes end-of-second-year distress; initial distress and cognitive-task performance are controlled.
Sardella et al. (2021)	Baseline EF precedes later HRQoL, but the baseline value of that outcome is not controlled in the reviewed follow-up model.
Southward and Cheavens (2017)	Baseline EF precedes one-month symptoms; baseline symptoms are controlled.
Wang et al. (2022)	EF and distress are both measured at T2. Controlling T1 distress does not make EF a prospectively measured exposure.
Wang et al. (2023)	Repeated EF/outcomes are modeled with prior levels, reciprocal paths and demographic controls; panel paths do not establish within-person causality.

Table S3 Randomized experimental contribution

JBI randomized controlled trial tool (2023), Q1–Q13. The reviewed experiment manipulates the expressive capacity displayed by an observed peer.

Study	Outcome	Q1	Q2	Q3	Q4	Q5	Q6	Q7	Q8	Q9	Q10	Q11	Q12	Q13
Wang et al. (2020) Experiment 1	Peer acceptance	Unclear	Unclear	Unclear	Unclear	Unclear	Yes	Unclear	Yes	Yes	Unclear	Unclear	Yes	Yes

Note. The retained sample is 147 participants. The report states random assignment but does not sufficiently document sequence generation, concealment, several aspects of blinding or the allocation of six excluded recruits. Unclear denotes these unresolved reporting gaps; it does not establish that randomization failed.

Scope of the experimental result

The outcome is immediate liking of a target shown in standardized videos. It is not a test of whether training a participant's own expressive flexibility improves clinical outcomes. Observational associations with symptom measures from other laboratory studies are appraised under their relevant observational design.

Using the supporting sheets

S5 provides the rationale and item-specific evidence locations for every judgment, including N/A and Unclear. For studies with several outcome rows, only differing items are repeated; unchanged items apply to all listed outcomes. Each location gives the published article page and section, with table or footnote identifiers where relevant. Pages spanning a section boundary are identified separately where possible. Article-specific pagination is used for journals with article numbers and for advance online publications. For absent information, the location identifies the section checked; it does not imply an explicit statement of absence. Applicability decisions based on the review's appraisal rules are labelled accordingly.

S4 Item definitions analytical cross-sectional studies

Item	Original question	Appraisal level
Q1	Were the criteria for inclusion in the sample clearly defined?	Study
Q2	Were objective, standard criteria used for measurement of the condition?	Outcome
Q3	Was the exposure measured in a valid and reliable way?	Study
Q4	Were the outcomes measured in a valid and reliable way?	Outcome
Q5	Were confounding factors identified?	Study
Q6	Were strategies to deal with confounding factors stated?	Outcome
Q7	Was appropriate statistical analysis used?	Result
Q8	Were the study subjects and the setting described in detail?	Study

Q1–Q6 concern internal validity; Q7 concerns statistical conclusion validity; Q8 concerns completeness of reporting. The revised eight-item order is used throughout.

Source: Barker TH, Hasanoff S, Aromataris E, et al. The revised JBI critical appraisal tool for the assessment of risk of bias for analytical cross-sectional studies. JBI Evidence Synthesis. 2026;24(3):401–408. https://doi.org/10.11124/JBIES-24-00523

Checklist source: JBI critical appraisal tools, https://jbi.global/critical-appraisal-tools (accessed 19 September 2026). Original questions are reproduced from the corresponding downloaded checklist; applicability rules are specified separately above.

S4 Item definitions cohort studies

Item	Original question	Appraisal level
Q1	Were the two groups similar and recruited from the same population?	Study
Q2	Were the exposures measured similarly to assign people to both the exposed and unexposed groups?	Study
Q3	Was the exposure measured in a valid and reliable way?	Study
Q4	Were confounding factors identified?	Study
Q5	Were strategies to deal with confounding factors stated?	Outcome
Q6	Were the groups/participants free of the outcome at the start of the study (or at the moment of the exposure)?	Outcome
Q7	Were the outcomes measured in a valid and reliable way?	Outcome
Q8	Was the follow-up time reported and sufficient to be long enough for outcomes to occur?	Outcome
Q9	Was follow-up complete, and if not, were the reasons for loss to follow-up described and explored?	Result
Q10	Were strategies to address incomplete follow-up utilized?	Result
Q11	Was appropriate statistical analysis used?	Result

Source: Barker TH, Hasanoff S, Aromataris E, et al. The revised JBI critical appraisal tool for the assessment of risk of bias for cohort studies. JBI Evidence Synthesis. 2025;23(3):441–453. https://doi.org/10.11124/JBIES-24-00103

Checklist source: JBI critical appraisal tools, https://jbi.global/critical-appraisal-tools (accessed 19 September 2026). Original questions are reproduced from the corresponding downloaded checklist; applicability rules are specified separately above.

S4 Item definitions randomized controlled experiments

Item	Original question	Appraisal level
Q1	Was true randomization used for assignment of participants to treatment groups?	Study
Q2	Was allocation to groups concealed?	Study
Q3	Were treatment groups similar at the baseline?	Study
Q4	Were participants blind to treatment assignment?	Study
Q5	Were those delivering the treatment blind to treatment assignment?	Study
Q6	Were treatment groups treated identically other than the intervention of interest?	Study
Q7	Were outcome assessors blind to treatment assignment?	Outcome
Q8	Were outcomes measured in the same way for treatment groups?	Outcome
Q9	Were outcomes measured in a reliable way?	Outcome
Q10	Was follow up complete and if not, were differences between groups in terms of their follow up adequately described and analysed?	Result
Q11	Were participants analysed in the groups to which they were randomized?	Result
Q12	Was appropriate statistical analysis used?	Result
Q13	Was the trial design appropriate and any deviations from the standard RCT design (individual randomization, parallel groups) accounted for in the conduct and analysis of the trial?	Study

Source: Barker TH, Stone JC, Sears K, et al. The revised JBI critical appraisal tool for the assessment of risk of bias for randomized controlled trials. JBI Evidence Synthesis. 2023;21(3):494–506.

Checklist source: JBI critical appraisal tools, https://jbi.global/critical-appraisal-tools (accessed 19 September 2026). Original questions are reproduced from the corresponding downloaded checklist; applicability rules are specified separately above.

S5 01 Ang and Tsai (2023)

Analytical cross-sectional studies | Baseline depression and life satisfaction

Item	Level / exception	Judgment	Rationale	Evidence location
Q1 Eligibility	Study	Yes	First-year students at a private New York university were recruited at the start of the semester; the study population and recruitment procedure are specified.	pp. 2–3; Methods, Participants and procedures.
Q2 Condition criteria	Outcome	N/A	No case definition was used for selection by this outcome; continuous symptom or well-being measurement is assessed under Q4.	p. 3; Measures, Satisfaction with life and Depressive symptoms (continuous outcomes; review rule).
Q3 EF measurement	Study	Yes	The standard FREE and its enhancement/suppression scoring are described, with internal consistency reported by ethnic group in Table 1.	p. 3; Measures, Expressive flexibility; p. 4, Table 1 (reliability).
Q4 Outcome measurement	Outcome	Yes	The SWLS and DASS depression subscale are established measures; sample-specific reliabilities are reported by ethnic group.	p. 3; Measures, Satisfaction with life and Depressive symptoms; p. 4, Table 1 (reliability).
Q5 Confounders identified	Study	Yes	Age, gender, baseline outcomes and habitual reappraisal/suppression are identified for the longitudinal analyses.	p. 3; Analytic plan.
Q6 Confounding strategy	Outcome	No	The T1 associations appraised here unadjusted correlations (Table 1). The authors adjust for covariates only in the models predicting T2 outcomes (Table 3); these are appraised in the cohort sheet.	p. 3; Analytic plan and Results; p. 4, Table 1 (zero-order correlations); p. 6, Table 3 (covariate-adjusted T2 models).
Q7 Statistical analysis	Result	Yes	The reported correlation/regression procedures fit the continuous-score association question. Unreported diagnostics are not assumed to have been checked; cross-sectional associations do not establish causality.	p. 3; Analytic plan; p. 4, Table 1.
Q8 Subjects and setting	Study	Yes	University type, city, recruitment methods, semester timing, age, sex and ethnic composition are reported.	pp. 2–3; Methods, Participants and procedures.

Note. The article reports 398 initially recruited participants and 279 complete cases.

S5 01 Ang and Tsai (2023)

Cohort studies | Later depression and life satisfaction

Item	Level / exception	Judgment	Rationale	Evidence location
Q1 Comparable groups	Study	N/A	No separately recruited exposed/unexposed groups: EF is a continuous predictor within one cohort. Q1 is not applicable to a two-group selection comparison; selection and attrition limitations are recorded elsewhere.	pp. 2–3; Participants and procedures (single cohort; review rule).
Q2 Consistent EF assessment	Study	Yes	Participants contributing to the comparison receive the same described EF measurement protocol; exposure ascertainment does not differ according to their score.	p. 3; Measures, Expressive flexibility.
Q3 EF measurement	Study	Yes	The FREE scoring and sample-specific reliability support measurement of perceived expressive ability.	p. 3; Measures, Expressive flexibility; p. 4, Table 1.
Q4 Confounders identified	Study	Yes	Age, gender, prior outcome levels and habitual regulation are identified.	p. 3; Analytic plan.
Q5 Confounding strategy	Outcome	Yes	Longitudinal regressions include baseline outcome, age, gender, reappraisal and habitual suppression.	p. 3; Analytic plan, hierarchical regression specification.
Q6 Outcome absent initially	Outcome	N/A	The outcome is a continuous symptom/well-being score, not incident disease. Being outcome-free at baseline is not an eligibility requirement for this estimand; temporal ordering and baseline adjustment are stated separately. EF is assessed at T1 before the week-13 outcomes.	pp. 2–3; Participants and procedures; p. 3, Analytic plan (timing and baseline adjustment; review rule).
Q7 Outcome measurement	Outcome	Yes	SWLS and DASS depression scores have stated measurement procedures and current reliability.	p. 3; Measures, Satisfaction with life and Depressive symptoms; p. 4, Table 1.
Q8 Follow-up interval	Outcome	Yes	The week-1 to week-13 interval is explicit and sufficient to observe changes during a college semester.	p. 2; Participants and procedures, first and thirteenth weeks.
Q9 Follow-up completeness	Result	No	Follow-up is incomplete: 119 of 398 recruits (30%) were lost by week 13, above the 20% level the JBI guidance treats as a substantial threat. Reasons for loss are not reported, and dropouts are compared only on age and gender, not on T1 EF, T1 outcomes or ethnicity.	pp. 2–3; Participants and procedures, loss to follow-up and retained sample.
Q10 Incomplete follow-up	Result	No	Analyses use complete cases; no weighting, imputation or sensitivity strategy addressing the missing follow-up outcomes is reported.	Not reported; checked pp. 2–3, Participants and procedures, and p. 3, Analytic plan.
Q11 Statistical analysis	Result	Yes	Baseline-adjusted regression and interaction models fit the longitudinal association question. Attrition remains a separate limitation.	p. 3; Analytic plan and Results, prospective relations.

Note. Large complete-case loss is the principal additional longitudinal concern.

S5 02 Ang and Tsai (2025)

Analytical cross-sectional studies | Life satisfaction, positive affect, hope and received social support

Item	Level / exception	Judgment	Rationale	Evidence location
Q1 Eligibility	Study	Yes	US adults aged over 18 with a specified MTurk approval rate were eligible; attention-check exclusions are stated.	p. 154; Methods, Participants and Procedures.
Q2 Condition criteria	Outcome	N/A	No case definition was used for selection by this outcome; continuous symptom or well-being measurement is assessed under Q4.	p. 155; Measures (continuous outcomes; review rule).
Q3 EF measurement	Study	Yes	The standard FREE is used; enhancement and suppression reliability and scoring are reported.	p. 155; Measures, expressive flexibility paragraph; Table 2 (reliability).
Q4 Outcome measurement	Outcome	Yes	SWLS, positive-affect items, the Hope Scale and the receiving-support subscale are described with published sources and current reliability estimates.	p. 155; Measures, life satisfaction, positive affect, hope and social support paragraphs; Table 2.
Q5 Confounders identified	Study	Yes	Age group and gender are explicitly included as explanatory/control variables.	p. 155; Analytic Plan, model input variables.
Q6 Confounding strategy	Outcome	Yes	Outcome models include age group, gender and the expressive-ability terms and interactions. Separate bivariate correlations remain unadjusted; the affirmative judgment applies to the reported outcome-level adjustment strategy.	p. 155; Analytic Plan; p. 156, Table 3 (unadjusted correlations); p. 157, Table 4 (adjusted models).
Q7 Statistical analysis	Result	Yes	The reported correlation/regression procedures fit the continuous-score association question. Unreported diagnostics are not assumed to have been checked; cross-sectional associations do not establish causality.	p. 155; Analytic Plan.
Q8 Subjects and setting	Study	Yes	Online platform, US eligibility, December 2021 collection period and detailed demographic characteristics are reported.	p. 154; Participants and Procedures; Table 1.

S5 03 Bonanno et al. (2004)

Cohort studies | Psychological distress at follow-up

Item	Level / exception	Judgment	Rationale	Evidence location
Q1 Comparable groups	Study	N/A	No separately recruited exposed/unexposed groups: EF is a continuous predictor within one cohort. Q1 is not applicable to a two-group selection comparison; selection and attrition limitations are recorded elsewhere.	p. 483; Method, Subjects and Procedure (single cohort; review rule).
Q2 Consistent EF assessment	Study	Yes	Participants contributing to the comparison receive the same described EF measurement protocol; exposure ascertainment does not differ according to their score.	pp. 483–484; Subjects and Procedure; p. 484, Observer Ratings of Emotional Expression.
Q3 EF measurement	Study	Yes	The standardized expressive task uses three observers blind to instructions/hypotheses and has ICC .91.	p. 484; Observer Ratings of Emotional Expression and Expressive-Regulation Task; p. 485, expressive-flexibility scoring.
Q4 Confounders identified	Study	Yes	Initial distress and cognitive-task performance are identified as possible alternative explanations.	p. 485; Expressive Flexibility as a Predictor of Change in Long-Term Adjustment.
Q5 Confounding strategy	Outcome	Yes	Hierarchical regressions control initial distress and performance on the filler cognitive task.	p. 485; Expressive Flexibility as a Predictor of Change in Long-Term Adjustment; p. 486, Table 2.
Q6 Outcome absent initially	Outcome	N/A	The outcome is a continuous symptom/well-being score, not incident disease. Being outcome-free at baseline is not an eligibility requirement for this estimand; temporal ordering and baseline adjustment are stated separately. Baseline distress is measured first, EF 1–3 months later and follow-up distress at the end of the second academic year.	p. 483; Subjects and Procedure (measurement sequence; review rule).
Q7 Outcome measurement	Outcome	Unclear	The report refers to a 32-item version of SCL-90-R, but does not sufficiently document the selected items, validation or reliability of this abbreviated outcome score.	p. 483; Subjects and Procedure, 32-item SCL-90-R. Abbreviated-score validation/reliability not reported there.
Q8 Follow-up interval	Outcome	Yes	Timing through the end of the second college year is reported and permits longitudinal symptom change.	p. 483; Subjects and Procedure, follow-up at end of second academic year.
Q9 Follow-up completeness	Result	Unclear	14 of 101 participants (14%) were lost before T2. Dropouts did not differ from completers on any measured variable, including baseline distress and task performance ($p > .15$), but reasons for loss are not given. The regressions use N = 81 (Table 2 df), so six further cases are missing without explanation.	p. 483, Subjects and Procedure; p. 486, Table 2.
Q10 Incomplete follow-up	Result	No	Only complete cases are analyzed: 87 participants completed follow-up, and the regression df in Table 2 imply N = 81. No imputation, weighting or sensitivity analysis is reported. Participants were paid $250 per study year; this may reduce dropout but does not address the outcomes that are still missing.	Not reported; checked p. 483, Subjects and Procedure, and p. 485, longitudinal regression description.
Q11 Statistical analysis	Result	Yes	Baseline-adjusted hierarchical regression fits the prospective question, subject to the outcome-measure and attrition qualifications.	p. 485; Expressive Flexibility as a Predictor of Change in Long-Term Adjustment; p. 486, Table 2.

Note. The particular 32-item SCL-90-R version is insufficiently specified to establish its validity and reliability.

S5 04 Burton and Bonanno (2016) Study 1

Analytical cross-sectional studies | Ego resilience and social functioning deficits

Additional outcome rows: Depressive symptoms.

Item	Level / exception	Judgment	Rationale	Evidence location
Q1 Eligibility	Study	Yes	Study 1 recruited an English-speaking US sample through SocialSci; the target population is specified. Only Study 1 contributes the reviewed outcomes.	p. 931; Study 1, Method.
Q2 Condition criteria	Outcome	N/A	No case definition was used for selection by this outcome; continuous symptom or well-being measurement is assessed under Q4.	p. 932; Measures, Social functioning and Ego resilience (continuous outcomes; review rule).
Q3 EF measurement	Study	Yes	The paper develops the FREE using scenario selection and confirmatory factor analyses; enhancement and suppression alpha values are .81 and .70.	pp. 931–932; Measures, Expressive regulation ability; p. 933, Results, hierarchical model and reliability.
Q4 Outcome measurement	Outcome	Yes	The LFQ and Ego Resiliency Scale are described with reliability estimates (.90 and .83). The depressive-symptom measure underlying the Table 1 correlation is not sufficiently specified.	p. 932; Measures, Social functioning and Ego resilience. Depression instrument not identified in Study 1 Measures, pp. 931–932; correlation appears in Table 1, p. 934.
Q4 Outcome measurement	Depressive symptoms	Unclear	The depressive-symptom measure underlying the correlation is not sufficiently identified to verify validity/reliability.	pp. 931–932; Study 1, Measures (depression measure not specified); p. 934, Table 1 (depression correlations).
Q5 Confounders identified	Study	No	For the Study 1 health associations, no explicit identification of potential confounders is reported; other constructs used for validation are not automatically confounders.	Not reported for health associations; checked p. 933, Comparing the FREE Scale with other measures of emotion regulation, personality, and adjustment, and Table 1, p. 934.
Q6 Confounding strategy	Outcome	No	The reviewed health associations are bivariate correlations without a stated confounding-control strategy.	p. 933; Comparing the FREE Scale with other measures of emotion regulation, personality, and adjustment; p. 934, Table 1 (bivariate correlations; no adjustment reported).
Q7 Statistical analysis	Result	Yes	Bivariate correlations are appropriate for the reported association question. The unidentified depression measure is addressed separately under Q4.	p. 933; Comparing the FREE Scale with other measures of emotion regulation, personality, and adjustment; p. 934, Table 1.
Q8 Subjects and setting	Study	Yes	The SocialSci US online sampling frame, language, sample size, age and sex composition are described.	p. 931; Study 1, Method.

Note. Depression requires Q4 Unclear because its measurement is not adequately identified.

S5 05 Chen et al. (2018)

Analytical cross-sectional studies | Depression, anxiety and life satisfaction

Additional outcome rows: Resilience.

Item	Level / exception	Judgment	Rationale	Evidence location
Q1 Eligibility	Study	No	The achieved undergraduate sample is described, but explicit eligibility criteria are not clearly specified; the word healthy does not supply operational criteria.	p. 79, Section 2.1, Participants and procedure; p. 83, Limitations ("healthy undergraduates"); explicit eligibility criteria not reported.
Q2 Condition criteria	Outcome	N/A	No case definition was used for selection by this outcome; continuous symptom or well-being measurement is assessed under Q4.	p. 79, Section 2.2.2; p. 80, Sections 2.2.4–2.2.5 (continuous outcomes; review rule).
Q3 EF measurement	Study	Yes	The Chinese FREE translation/back-translation and factor analysis are reported; enhancement and suppression alpha values are .82 and .78.	p. 79; Section 2.2.1, Expressive regulation ability; p. 80, Section 3.1, Confirmatory factor analysis.
Q4 Outcome measurement	Outcome	Yes	BAI, SWLS and CD-RISC have cited validation and alpha values .91, .84 and .92. The BDI-II suicide item was transparently omitted; its 20-item continuous severity score (alpha .87) is accepted for this purpose.	p. 79; Section 2.2.2, Resilience; p. 80, Sections 2.2.4, Psychopathology symptoms, and 2.2.5, Life Satisfaction.
Q5 Confounders identified	Study	Yes	Age, gender, reappraisal, habitual suppression and rumination are explicitly identified as covariates.	p. 80; Section 2.3, Statistical analyses.
Q6 Confounding strategy	Outcome	Yes	Hierarchical models for depression, anxiety and life satisfaction include the specified covariates. Resilience has only bivariate correlations. Separate bivariate correlations remain unadjusted; the affirmative judgment applies to the reported outcome-level adjustment strategy.	p. 80; Section 2.3 and Section 3.3, Regression analyses; p. 81, Table 1 (resilience: zero-order correlation only).
Q6 Confounding strategy	Resilience	No	Resilience is reported only in zero-order correlations, without a stated adjusted EF–resilience model.	p. 80; Section 2.3, Statistical analysis; p. 81, Table 1 (resilience correlations).
Q7 Statistical analysis	Result	Yes	The correlations, hierarchical regressions, interaction terms and simple-slope analyses fit the stated questions. Missing diagnostic reporting alone does not show an unsuitable analysis.	p. 80; Sections 2.3 and 3.3, including simple-slope analyses.
Q8 Subjects and setting	Study	No	Size, age, gender, online administration and compensation are described, but the recruitment setting/sampling frame and dates are not sufficiently detailed. Author affiliation and ethics approval do not establish recruitment location.	p. 79; Section 2.1, Participants and procedure; recruitment frame/location and dates not specified.

Note. The abbreviated BDI-II is accepted for continuous symptom severity. This does not validate diagnostic cutoffs for the modified score.

S5 06 Dwyer et al. (2024)

Analytical cross-sectional studies | Depression, anxiety and stress

Item	Level / exception	Judgment	Rationale	Evidence location
Q1 Eligibility	Study	Yes	MTurk eligibility, US residence/approval-rate requirements, AUDIT threshold and data-quality exclusions are described. The substantial exclusions limit representativeness.	p. 189; Methods, Participants; p. 191, Data quality (eligibility and exclusions).
Q2 Condition criteria	Outcome	Yes	AUDIT ≥16 defines the alcohol-use eligibility threshold. This is a standardized screening criterion, not proof of a formal alcohol-use-disorder diagnosis.	p. 190; Clinical measures, Alcohol use disorders identification test; p. 189, Participants (AUDIT threshold).
Q3 EF measurement	Study	Yes	The published FREE is administered with the stated total-score approach. The summed repertoire score is distinguished from polarity-corrected EF; absence of a new alpha alone does not invalidate the established measure.	p. 190; Regulatory flexibility measures, The flexible regulation of emotional expression scale.
Q4 Outcome measurement	Outcome	Yes	Depression, anxiety and stress are measured with the established DASS-21; no sample-specific reliability is reported. This is symptom measurement, not diagnostic ascertainment.	p. 190; Clinical measures, Depression anxiety stress scale.
Q5 Confounders identified	Study	Yes	Demographic covariates are considered in the profile analyses, but those analyses are distinct from the separate FREE–symptom associations.	p. 191; Statistical analyses, additional demographic covariates.
Q6 Confounding strategy	Outcome	No	The FREE–DASS results are unadjusted correlations. Covariate adjustment of latent-profile comparisons cannot be transferred to them.	p. 191; Statistical analyses, final paragraph (Pearson correlations; covariate selection concerns profile comparisons).
Q7 Statistical analysis	Result	Yes	Pearson correlations are a suitable method for the stated association. Reporting only a nonsignificance threshold leaves the individual coefficients unavailable and prevents effect-size extraction; this limitation is retained separately.	p. 191; Statistical analyses, Pearson correlations; p. 193, Delay discounting, depression, anxiety, and stress associations, final sentence.
Q8 Subjects and setting	Study	Yes	Platform, screening, recruitment restrictions, exclusion flow and final sample characteristics are reported.	p. 189; Participants; p. 191, Data quality and Results; p. 192, Table 1.

Note. Individual FREE–DASS coefficients are not reported in the main article. Nonsignificance does not establish zero effects; the appraisal concerns the reported methods and main-article results.

S5 07 Gonzalez-Escamilla et al. (2022)

Analytical cross-sectional studies | Depression, anxiety, stress, well-being and resilience

Item	Level / exception	Judgment	Rationale	Evidence location
Q1 Eligibility	Study	Yes	Recruitment waves and exclusions are specified; the second wave adds language and medication restrictions, which may change sample composition.	p. 4; Participants and Procedure.
Q2 Condition criteria	Outcome	N/A	No case definition was used for selection by this outcome; continuous symptom or well-being measurement is assessed under Q4.	p. 5; Assessment Of Psychopathological Symptoms and Mental Health (continuous outcomes; review rule).
Q3 EF measurement	Study	Yes	German translation procedures, confirmatory factor analysis and reliability are reported for the FREE.	pp. 4–5; Flexibility in Emotional Expressive Regulation; p. 7, Evaluation and Validation of the FREE Scale (German Version), Table 1.
Q4 Outcome measurement	Outcome	Yes	DASS-21, WHO-5 and RS-11 are identified with validation sources and current internal consistency estimates.	p. 5; Psychopathology Symptoms, Well-Being and Coping Strategies; p. 7, Table 1.
Q5 Confounders identified	Study	Yes	Age and gender are explicitly considered in the health-association analyses.	p. 6; Statistical Analyses, Association Between Flexibility in Emotional Expressive Regulation With Indicators of Psychopathology Symptoms and Mental Health.
Q6 Confounding strategy	Outcome	Yes	Partial rank correlations and association models include age and gender. These are adjusted estimates, unlike an ordinary Pearson correlation.	p. 6; Association Between Flexibility in Emotional Expressive Regulation With Indicators of Psychopathology Symptoms and Mental Health (age/gender covariates, rank partial correlations, GLM specification); p. 9, Results; p. 11, Table 4 (partial rank correlations).
Q7 Statistical analysis	Result	Yes	Rank-based association analysis, penalized predictor selection and reported multiplicity correction fit the stated exploratory aims. Selection-based findings should not be interpreted as confirmed causal effects.	pp. 6–7; Statistical Analyses (LASSO, partial correlations, FDR and GLM).
Q8 Subjects and setting	Study	Yes	Recruitment sources, German setting, two collection periods, exclusions and demographic information are described.	p. 4; Participants and Procedure; p. 7, Results, Participants.

S5 08 Gupta and Bonanno (2011)

Analytical cross-sectional studies | Complicated grief group comparisons

Item	Level / exception	Judgment	Rationale	Evidence location
Q1 Eligibility	Study	Yes	Age, bereavement interval, socioeconomic requirements and prior chronic-depression exclusions are specified; bereaved and married comparison recruitment is described.	p. 637; Method, Participants.
Q2 Condition criteria	Outcome	Yes	Complicated grief is defined by explicit interview-based research criteria. These are the study's criteria, not a claim of current DSM diagnostic equivalence.	p. 637; Structured Clinical Interview, CG symptom criteria; p. 640, footnote 2 (alternative definition).
Q3 EF measurement	Study	Yes	The standardized expressive task uses counterbalanced stimuli and independent observers blind to condition; interrater ICC is .89.	p. 638; Expressive Flexibility Task, randomized blocks and blind observer ratings (ICC .89).
Q4 Outcome measurement	Outcome	Yes	Interview-based grief assessment uses specified symptom criteria, trained interviewers and reported reliability.	p. 637; Structured Clinical Interview, interviewer training and reliability.
Q5 Confounders identified	Study	Yes	Depression is identified as a potential alternative explanation for the grief-group findings.	p. 640; footnote 3 (depression as covariate).
Q6 Confounding strategy	Outcome	Yes	The paper repeats the group analyses with depression as a covariate. This supports an outcome-level Yes while leaving residual confounding possible.	p. 640; footnote 3 (repeated analyses controlling depression).
Q7 Statistical analysis	Result	Yes	Repeated-measures group comparisons and the reported depression-adjusted checks match the design. Group differences do not establish the direction of causality.	p. 639; Enhancement and Suppression Ability; p. 640, group-comparison analyses and footnote 3.
Q8 Subjects and setting	Study	Yes	New York recruitment routes, bereavement and comparison samples, eligibility and demographic characteristics are reported.	p. 637; Participants and Procedure.

Note. The reviewed result is an observational group comparison; performing a standardized expressive task does not make grief status a randomized exposure.

S5 09 Lenzo et al. (2020)

Analytical cross-sectional studies | Emotional exhaustion, depersonalization and personal accomplishment

Item	Level / exception	Judgment	Rationale	Evidence location
Q1 Eligibility	Study	Yes	Full-time palliative-care work and health-related exclusions are stated.	p. 3; Materials and methods, Participants.
Q2 Condition criteria	Outcome	Unclear	A history of psychiatric disorders is excluded, but the report does not sufficiently identify standardized ascertainment of that exclusion.	p. 3; Participants, psychiatric-history exclusion; standardized ascertainment not specified.
Q3 EF measurement	Study	Unclear	The standard 16-item FREE and current reliability are reported. The specific Italian translation/validation used in this sample is insufficiently documented.	p. 4; Measures, item 2 (FREE); specific Italian adaptation/validation not specified.
Q4 Outcome measurement	Outcome	Yes	The Italian Maslach Burnout Inventory is identified with validation sources and subscale alpha values .85, .73 and .77.	p. 4; Measures, item 1 (MBI).
Q5 Confounders identified	Study	Yes	Age, work experience and context sensitivity are identified in the analysis strategy.	p. 5; Statistical analyses, ANCOVA paragraph.
Q6 Confounding strategy	Outcome	Yes	The ANCOVA strategy includes age, work experience and CSI. This does not imply that all occupational confounding was eliminated.	p. 5; Statistical analyses, age/work-experience and FREE/CSI model steps.
Q7 Statistical analysis	Result	Unclear	ANOVA/ANCOVA procedures fit the comparisons of continuous burnout scores, but participants worked in teams of two, and this dependence is not addressed in the analysis. Its impact on standard errors cannot be determined.	pp. 4–5, Statistical analyses; p. 3, Participants, and p. 5, Results (teams of two; dependence not addressed).
Q8 Subjects and setting	Study	Yes	Palliative-care organizations in Messina and Agrigento, work setting and demographic/occupational characteristics are reported.	p. 3; Participants and Procedure; p. 5, Results and Table 1.

Note. The administered Italian FREE version is not identified sufficiently to establish its measurement validity. The Unclear judgment at Q7 reflects unaddressed dependence within two-person teams; it does not establish that the estimates are biased.

S5 10 Lenzo et al. (2021)

Analytical cross-sectional studies | Depression, anxiety and stress

Item	Level / exception	Judgment	Rationale	Evidence location
Q1 Eligibility	Study	Yes	Adult full-time healthcare-worker eligibility and the recruitment procedure during lockdown are stated.	p. 3; Materials and Methods, Participants and Procedure.
Q2 Condition criteria	Outcome	N/A	No case definition was used for selection by this outcome; continuous symptom or well-being measurement is assessed under Q4.	pp. 3–4; Measures, Depression, Anxiety, and Stress (continuous outcomes; review rule).
Q3 EF measurement	Study	Unclear	The standard FREE is described, but local language adaptation/psychometric support for the specific version is not sufficiently documented. Alpha is deliberately not computed; that omission alone is not the reason for Unclear.	p. 4; Emotion Regulation Ability and Statistical Analyses; specific local adaptation not documented.
Q4 Outcome measurement	Outcome	Yes	The Italian DASS-21 is referenced and current subscale alpha values are .83, .78 and .87.	pp. 3–4; Measures, Depression, Anxiety, and Stress; p. 4 gives Italian validation and reliability.
Q5 Confounders identified	Study	Yes	Age, gender and work experience are identified as covariates.	p. 4; Statistical Analyses, hierarchical regressions.
Q6 Confounding strategy	Outcome	Yes	Multivariable health-outcome models include demographic/occupational covariates alongside flexibility/context-sensitivity terms. Separate bivariate correlations remain unadjusted; the affirmative judgment applies to the reported outcome-level adjustment strategy.	p. 4; Statistical Analyses, two-step models and separate Pearson correlations.
Q7 Statistical analysis	Result	Yes	The reported correlation/regression procedures fit the continuous-score association question. Unreported diagnostics are not assumed to have been checked; cross-sectional associations do not establish causality.	p. 4; Statistical Analyses.
Q8 Subjects and setting	Study	Yes	Sicilian healthcare setting, collection dates in April–May 2020, recruitment and sample characteristics are given.	p. 3; Participants and Procedure; Table 1.

Note. The identity of the administered Italian FREE version is insufficiently documented; a later validation does not establish the properties of the earlier version.

S5 11 Levy-Gigi et al. (2020)

Analytical cross-sectional studies | PTSD symptoms

Item	Level / exception	Judgment	Rationale	Evidence location
Q1 Eligibility	Study	Yes	Operational firefighter recruitment and psychiatric, neurological and substance-related exclusions are specified.	p. 3; Section 2.1, Participants.
Q2 Condition criteria	Outcome	Unclear	Psychiatric exclusions are stated without enough information about standardized diagnostic ascertainment.	p. 3; Section 2.1, Participants; diagnostic ascertainment for exclusions not specified.
Q3 EF measurement	Study	Unclear	The FREE task scenarios and scoring are described, but language adaptation and psychometric support for the version used with Israeli firefighters are insufficiently documented.	pp. 3–4; Section 2.2, Measures, FREE; language adaptation/validation not specified.
Q4 Outcome measurement	Outcome	Unclear	The article describes PCL-5 as 21 items scored 1–5. The instrument developer describes 20 items scored 0–4. The report does not resolve whether this is a reporting error or a modified administration.	p. 4; Section 2.2, Measures, PCL-5 paragraph (21 items, 1–5 reported); discrepancy unresolved in article.
Q5 Confounders identified	Study	No	The focal trauma-exposure predictor and EF moderator are not a separate strategy for identifying other confounders; no clear additional confounder set is stated.	Not reported; checked p. 4, Section 2.4, Data Analysis (trauma exposure and EF moderation model).
Q6 Confounding strategy	Outcome	No	No additional covariate-adjustment strategy for the EF–PTSD association is reported.	p. 4; Section 2.4, Data Analysis; additional covariates not specified.
Q7 Statistical analysis	Result	Yes	Regression/moderation with bootstrap confidence intervals is appropriate for the associational model. The measurement discrepancy is evaluated under Q4.	p. 4; Section 2.4, Data Analysis (PROCESS, 5000 bootstrap samples).
Q8 Subjects and setting	Study	Yes	Fire-station and academy settings, Israeli recruitment, occupational exposure and sample demographics are described.	p. 3; Section 2.1, Participants; p. 4, Section 2.3, Procedure.

Note. The reported PCL-5 item count and scoring differ from the standard instrument description; the FREE language version is also insufficiently documented.

Instrument comparison: US Department of Veterans Affairs, National Center for PTSD. PTSD Checklist for DSM-5 (PCL-5). https://www.ptsd.va.gov/professional/assessment/adult-sr/ptsd-checklist.asp (accessed 19 September 2026).

S5 12 Maccallum et al. (2021)

Analytical cross-sectional studies | Anxiety, depression, stress and trait-anxiety group comparisons

Item	Level / exception	Judgment	Rationale	Evidence location
Q1 Eligibility	Study	Unclear	Recruitment from UNSW and screening for no versus high anxiety are described, but numerical screening thresholds for initial eligibility are not supplied.	p. 936; Section 2.1, Participants; initial DASS screening thresholds not specified.
Q2 Condition criteria	Outcome	Unclear	The STAI median split (41.5) is explicit, but the DASS thresholds used to select the sample are not. This is symptom-based selection, not ascertainment of anxiety-disorder diagnoses.	p. 936; Section 2.1, Participants (STAI median 41.50); p. 937, Section 3.1, Self-report measures (DASS screening).
Q3 EF measurement	Study	Yes	The expressive task has controlled stimulus/instruction procedures, observers blind to hypotheses and high agreement on double-coded recordings.	p. 937; Section 3.1, Expressive flexibility task and Scoring.
Q4 Outcome measurement	Outcome	Yes	The standard DASS-21 and STAI trait measures and scoring are described with sources; the trait groups are sample-relative groups.	p. 937; Section 3.1, Self-report measures, DASS-21 and STAI-Y Trait.
Q5 Confounders identified	Study	Yes	Age, depressive/stress symptoms and working memory are examined as possible alternative explanations for trait-anxiety group differences.	p. 937; Section 4.1, Participant Characteristics; p. 938, alternative-variable correlations.
Q6 Confounding strategy	Outcome	No	The extracted associations are correlations and group comparisons. Checking separate correlations with potential confounders does not adjust the EF–anxiety association.	p. 938; Results, EF correlations and group comparisons; no joint adjustment reported.
Q7 Statistical analysis	Result	Yes	The reported group comparisons and correlations fit the descriptive association question. Median splitting loses information; the continuous analyses are retained separately.	p. 938; Results, expressive-flexibility analyses; p. 936, Section 2.1 (median split).
Q8 Subjects and setting	Study	Yes	UNSW recruitment, compensation, screening, exclusions and group demographic characteristics are reported.	p. 936; Section 2.1, Participants; Table 1.

Note. Initial DASS eligibility thresholds are unclear. Median-split anxiety groups do not establish clinical diagnoses.

S5 13 Nardelli et al. (2024)

Analytical cross-sectional studies | Depression, anxiety, stress, total distress and borderline symptoms

Item	Level / exception	Judgment	Rationale	Evidence location
Q1 Eligibility	Study	Yes	French-speaking adults aged at least 18 and attention-check exclusion rules are explicitly specified.	p. 717; Method, Participants; p. 718, Attention Checks.
Q2 Condition criteria	Outcome	N/A	No case definition was used for selection by this outcome; continuous symptom or well-being measurement is assessed under Q4.	p. 718; Measures, DASS-21 and BSL-23 (continuous outcomes; review rule).
Q3 EF measurement	Study	Yes	Translation/back-translation, pilot testing, cross-language checks, factor analyses and FREE reliability are reported; enhancement/suppression omega values are .85/.78.	p. 717; Translation and Back-Translation Procedure and Measures, FREE; p. 719, Internal Consistency of the FREE and Test-Retest Reliability for the FREE and CSI.
Q4 Outcome measurement	Outcome	Yes	DASS-21 and BSL-23 measures, scoring and strong current internal consistency estimates are reported.	p. 718; Measures, Depression, Anxiety and Stress Scale and Borderline Symptoms List.
Q5 Confounders identified	Study	No	The reviewed validity correlations are not accompanied by an explicit confounder-identification strategy; collecting demographics alone does not demonstrate one.	Not reported for validity correlations; checked p. 718, Statistical Analyses.
Q6 Confounding strategy	Outcome	No	The health associations are bivariate correlations. Benjamini–Hochberg correction controls multiplicity, not confounding.	p. 718; Statistical Analyses, final paragraph (Pearson correlations and Benjamini–Hochberg correction).
Q7 Statistical analysis	Result	Yes	Pearson validity correlations and multiplicity correction fit the association question. The separate one-month test–retest component is not the health-outcome contribution.	p. 718; Statistical Analyses and Procedure (health measures at first time point).
Q8 Subjects and setting	Study	Yes	French community sampling, social-media/snowball recruitment, eligibility and demographic characteristics are described.	p. 717; Participants; p. 719, Results, Descriptive data; Table 1.

S5 14 Pițur and Miu (2020)

Analytical cross-sectional studies | Depression and trait anxiety

Item	Level / exception	Judgment	Rationale	Evidence location
Q1 Eligibility	Study	No	The Romanian undergraduate sample and exclusions are described, but explicit initial eligibility criteria are not provided.	p. 1722; Materials and methods, Participants; initial eligibility criteria not specified.
Q2 Condition criteria	Outcome	N/A	No case definition was used for selection by this outcome; continuous symptom or well-being measurement is assessed under Q4.	p. 1723; Measures, Self-report questionnaires (continuous outcomes; review rule).
Q3 EF measurement	Study	Yes	The expressive task uses standardized instructions and observer ratings; ICC is .82.	p. 1723; Expressive flexibility paradigm; p. 1724, Expressive flexibility: manipulation check (ICC .82).
Q4 Outcome measurement	Outcome	Yes	BDI-II and STAI are established symptom measures. Reported omega-hierarchical estimates are modest (.60/.66); they are not treated as interchangeable with alpha or as automatic invalidation.	p. 1723; Self-report questionnaires, BDI-II and STAI paragraph.
Q5 Confounders identified	Study	Yes	Childhood maltreatment and related symptom variables are considered in the broader analyses as alternative or overlapping explanatory variables.	p. 1723; Statistical analysis, maltreatment and psychopathology models.
Q6 Confounding strategy	Outcome	No	The extracted EF–depression/anxiety associations are zero-order Spearman correlations. Adjustment in maltreatment-focused models does not control these estimates.	p. 1726; Table 1, zero-order associations; pp. 1723–1724, Statistical analysis.
Q7 Statistical analysis	Result	Yes	Spearman correlations suit the reported rank associations; no causal direction is inferred.	p. 1724; distribution tests under Expressive flexibility: manipulation check; p. 1726, Table 1 (Spearman correlations).
Q8 Subjects and setting	Study	No	Country, undergraduate status, demographics and exclusions are given; the recruitment setting/frame is insufficiently described independently of author affiliation.	p. 1722; Participants; specific recruitment frame not reported.

S5 15 Quattropani et al. (2022)

Analytical cross-sectional studies | Physical and psychological health-related quality of life

Item	Level / exception	Judgment	Rationale	Evidence location
Q1 Eligibility	Study	No	The online sample is characterized, but clearly operationalized inclusion/exclusion criteria are not reported.	p. 699; Method, Participants and procedure; operational eligibility criteria not reported.
Q2 Condition criteria	Outcome	N/A	No case definition was used for selection by this outcome; continuous symptom or well-being measurement is assessed under Q4.	p. 700; Measures, Perceived psychological and physical health (continuous outcomes; review rule).
Q3 EF measurement	Study	Yes	Italian translation/back-translation, focus groups, factor analyses and current FREE reliability support measurement of perceived expressive ability.	pp. 699–700; Measures, Expressive regulation ability; p. 700, Confirmatory factor analysis.
Q4 Outcome measurement	Outcome	Yes	The Italian SF-12 is identified and its physical/mental components are described. The physical component has modest internal consistency; no mechanical alpha cutoff is applied.	p. 700; Measures, Perceived psychological and physical health.
Q5 Confounders identified	Study	Yes	Age, gender and trait emotional intelligence are identified as covariates.	p. 700; Statistical analyses, regression covariates.
Q6 Confounding strategy	Outcome	Yes	Outcome regressions include age, gender and emotional-intelligence variables, allowing assessment of incremental expressive-ability associations.	p. 700; Statistical analyses, three-step hierarchical models.
Q7 Statistical analysis	Result	Yes	The reported correlation/regression procedures fit the continuous-score association question. Unreported diagnostics are not assumed to have been checked; cross-sectional associations do not establish causality.	p. 700; Statistical analyses.
Q8 Subjects and setting	Study	No	Demographic details are reported, but the online recruitment frame and setting are insufficiently specified. Affiliation alone is not treated as evidence of recruitment location.	p. 699; Participants and procedure; recruitment frame and setting not specified.

S5 16 Rodin et al. (2017)

Analytical cross-sectional studies | PTSD severity and PTSD/depression screening groups

Additional outcome rows: Depressive symptom severity.

Item	Level / exception	Judgment	Rationale	Evidence location
Q1 Eligibility	Study	Yes	Combat-veteran eligibility, recruitment, medication-stability requirements and exclusions are reported.	p. 237; Section 2.1, Participants.
Q2 Condition criteria	Outcome	Yes	Explicit PDS and BDI-II cutoffs define probable PTSD/depression groups. These are screening classifications, not independent structured diagnostic interviews.	p. 237; Sections 2.2.1, PDS, and 2.2.2, BDI-II (screening cutoffs).
Q3 EF measurement	Study	Yes	Independent observers blind to clinical group, instructions and stimuli rate the expressive task; interrater agreement is reported.	p. 238; Section 2.2.4, Expressive flexibility task, observer-ratings and scoring paragraphs.
Q4 Outcome measurement	Outcome	Yes	PDS and BDI-II are established symptom measures. Their cutoff-based group labels are interpreted as probable/screen-positive status.	p. 237; Sections 2.2.1–2.2.2, PDS and BDI-II.
Q5 Confounders identified	Study	Yes	Combat exposure is identified as a relevant covariate.	p. 238; Section 3.3, Expressive flexibility and PTSD (rationale for combat-exposure covariate).
Q6 Confounding strategy	Outcome	Yes	Group ANCOVAs and the extracted PTSD-severity regression (Table 2, Model 2) adjust for combat exposure.	p. 238, Sections 3.3–3.5.1; p. 239, Table 2.
Q6 Confounding strategy	Depressive symptom severity	No	The extracted depression-severity estimate (Table 3, Model 1) is not adjusted for combat exposure.	p. 238, Section 3.5.2; p. 239, Table 3, Model 1.
Q7 Statistical analysis	Result	Yes	Repeated-measures ANCOVA and regression fit the stated group/continuous associations. The diagnostic interpretation of screening groups remains limited.	p. 238; Sections 3.3–3.5.2; p. 239, Tables 2–3.
Q8 Subjects and setting	Study	Yes	Veteran recruitment sources, clinical context, eligibility and demographic characteristics are described.	p. 237; Participants; p. 238, Section 3.1 and Table 1.

Note. The clinical groups are defined by screening thresholds and are therefore described as probable or screen-positive groups.

S5 17 Sardella et al. (2021)

Analytical cross-sectional studies | Baseline physical health-related quality of life

Additional outcome rows: Baseline psychological health-related quality of life.

Item	Level / exception	Judgment	Rationale	Evidence location
Q1 Eligibility	Study	Yes	Age ≥65, neurocognitive, physical and sensory criteria are explicitly stated.	p. 3; Section 2.2, Participants and Procedure.
Q2 Condition criteria	Outcome	Yes	DSM-5-informed assessment and MMSE threshold, with physical/sensory exclusions, operationalize clinical eligibility.	p. 3; Section 2.2 (MMSE and functional/sensory exclusions); p. 4, Section 2.4, MMSE.
Q3 EF measurement	Study	Unclear	The FREE is described, but validation/translation and reliability of the specific Italian version in this very old sample (M = 80,26, SD = 6,39) are insufficiently documented.	p. 4; Section 2.4, Measures and Instruments, FREE paragraph; specific Italian validation not provided.
Q4 Outcome measurement	Outcome	Yes	The established SF-12 physical and mental summary scores are described. Interview procedures and the clinical assessment context are specified.	p. 4; Section 2.4, SF-12 paragraph.
Q5 Confounders identified	Study	Yes	Age/gender, education, cognitive status, frailty and optimism are considered as explanatory or confounding factors.	pp. 4–5; Section 2.5, Data Analysis, hierarchical covariate selection.
Q6 Confounding strategy	Outcome	Yes	Baseline PCS models include demographic, frailty and optimism variables. Baseline MCS associations extracted for this review are correlations without an EF-specific adjusted estimate.	pp. 4–5; Section 2.5; p. 6, Section 3.2 (adjusted baseline models) and Table 2 (unadjusted correlations).
Q6 Confounding strategy	Baseline psychological health-related quality of life	No	The extracted baseline mental-HRQoL association is unadjusted; the adjusted physical-HRQoL model cannot confer control on it.	p. 6; Section 3.2, baseline MCS regression description and Table 2 (unadjusted correlations).
Q7 Statistical analysis	Result	Yes	The reported correlation/regression procedures fit the continuous-score association question. Unreported diagnostics are not assumed to have been checked; cross-sectional associations do not establish causality.	pp. 4–5; Section 2.5, Data Analysis.
Q8 Subjects and setting	Study	Yes	The Messina geriatric clinic, recruitment dates, eligibility, assessment procedures and demographic/clinical characteristics are reported.	p. 3; Sections 2.1–2.2; p. 5, Section 3.1 and Table 1.

Note. The specific Italian FREE version is insufficiently documented.

S5 17 Sardella et al. (2021)

Cohort studies | Follow-up physical and mental health-related quality of life

Item	Level / exception	Judgment	Rationale	Evidence location
Q1 Comparable groups	Study	N/A	No separately recruited exposed/unexposed groups: EF is a continuous predictor within one cohort. Q1 is not applicable to a two-group selection comparison; selection and attrition limitations are recorded elsewhere.	p. 3; Sections 2.1–2.2 (single outpatient cohort; review rule).
Q2 Consistent EF assessment	Study	Yes	Participants contributing to the comparison receive the same described EF measurement protocol; exposure ascertainment does not differ according to their score.	p. 4; Section 2.4, FREE paragraph.
Q3 EF measurement	Study	Unclear	The specific Italian FREE version and its psychometric support in the geriatric sample are not adequately established in the report.	p. 4; Section 2.4, FREE paragraph; specific Italian validation/reliability not reported.
Q4 Confounders identified	Study	Yes	Demographic, cognitive, frailty and optimism variables are identified.	pp. 4–5; Section 2.5, Data Analysis.
Q5 Confounding strategy	Outcome	Yes	Multivariable follow-up models include relevant demographic/clinical/psychological factors, but do not adjust for the baseline value of the same HRQoL outcome; they cannot be interpreted as EF predicting change.	p. 5; Section 2.5, hierarchical covariates; p. 8, Table 5 (follow-up models; baseline HRQoL absent).
Q6 Outcome absent initially	Outcome	N/A	The outcome is a continuous symptom/well-being score, not incident disease. Being outcome-free at baseline is not an eligibility requirement for this estimand; temporal ordering and baseline adjustment are stated separately. EF precedes follow-up, but baseline HRQoL is not controlled in the reviewed models.	p. 3; Section 2.2 (timing); p. 8, Table 5 (baseline HRQoL not included; review rule).
Q7 Outcome measurement	Outcome	Yes	SF-12 scores are measured at baseline and by a trained psychologist by telephone at follow-up. The change of administration mode is a comparability limitation.	p. 4; Section 2.4, SF-12; p. 3, Section 2.2, telephone follow-up.
Q8 Follow-up interval	Outcome	Yes	Baseline October 2018–October 2019 and follow-up April 2020 give a reported interval of roughly 6–18 months, allowing change but differing across participants.	p. 3; Section 2.2, baseline and follow-up dates.
Q9 Follow-up completeness	Result	Unclear	104 of 141 are followed (26% loss): 26 are unreachable and 11 decline. Reasons are enumerated, but outcome-related differences between retained/lost participants are not explored sufficiently.	p. 6; Section 3.3, Characteristics of the Sample Evaluated during the COVID-19 Outbreak (completion and reasons for loss).
Q10 Incomplete follow-up	Result	No	Telephone contact is the stated follow-up mode; no additional strategy addressing the 37 missing outcomes or varying follow-up duration is reported.	Not reported; checked p. 3, Section 2.2; pp. 4–5, Section 2.5; p. 6, Section 3.3.
Q11 Statistical analysis	Result	Yes	Regression of later HRQoL is suitable for an association with later level. Data-driven selection and omission of baseline HRQoL limit claims about change or independent prospective effects.	pp. 4–5; Section 2.5; p. 8, Table 5 (later HRQoL models).

Note. The follow-up coefficients do not estimate change in HRQoL because the model does not adjust for the baseline outcome.

S5 18 Shangguan et al. (2022)

Analytical cross-sectional studies | Depression, life satisfaction and social support

Item	Level / exception	Judgment	Rationale	Evidence location
Q1 Eligibility	Study	No	College status and achieved ages are given, but explicit initial eligibility criteria are not clearly stated.	p. 5; Section 2.1, Participants and Procedure; initial eligibility criteria not separately specified.
Q2 Condition criteria	Outcome	N/A	No case definition was used for selection by this outcome; continuous symptom or well-being measurement is assessed under Q4.	p. 5; Sections 2.2.2–2.2.4 (continuous outcomes; review rule).
Q3 EF measurement	Study	Yes	The validated Chinese FREE and polarity-corrected EF score are described, with alpha .84 for the overall scale and .74/.79 for the abilities.	p. 5; Section 2.2.1, Expressive Flexibility.
Q4 Outcome measurement	Outcome	Yes	Chinese BDI, SWLS and PSSS are identified with strong current reliability (.93, .87 and .95).	p. 5; Sections 2.2.2, Social Support; 2.2.3, Depression; 2.2.4, Life Satisfaction.
Q5 Confounders identified	Study	Yes	Demographic control variables are identified; Methods and Results differ in whether grade or gender is named.	p. 6; Section 2.3, Data Analysis (age/grade); Section 3.3, Mediation Analyses (gender/age).
Q6 Confounding strategy	Outcome	Yes	The extracted mediation estimates for depression and life satisfaction are adjusted for age and gender (Results; Methods name age and grade). Separate bivariate correlations remain unadjusted.	p. 6; Sections 2.3 and 3.3; Table 1 (unadjusted correlations).
Q6 Confounding strategy	Perceived Social Support	No	The extracted EF–social support association is a zero-order correlation (Table 1); the adjusted path within the mediation model was not extracted.	p. 6, Table 1 (zero-order correlations).
Q7 Statistical analysis	Result	Yes	Correlations and bootstrapped path models fit an atemporal association question. They do not establish the temporal order or causal mediation suggested by the conceptual diagram.	p. 6; Section 2.3, Data Analysis.
Q8 Subjects and setting	Study	No	China, student status and online/offline administration are given, but the university sampling frame, recruitment period and setting are insufficiently detailed.	p. 5; Section 2.1, Participants and Procedure; university sampling frame and dates not specified.

Note. The covariate descriptions differ between Methods (age/grade) and Results (age/gender). The extracted social-support coefficient is unadjusted.

S5 19 Shangguan et al. (2024)

Analytical cross-sectional studies | Trait anxiety

Item	Level / exception	Judgment	Rationale	Evidence location
Q1 Eligibility	Study	Yes	Eligibility is restricted to students reporting normal mental health and no diagnosed mental illness; recruitment and age range are described.	p. 26125; Methods, Participants and procedure.
Q2 Condition criteria	Outcome	Unclear	Mental-health eligibility relies on reported absence of illness; standardized diagnostic ascertainment is not specified.	p. 26125; Participants and procedure, mental-health eligibility; standardized diagnostic ascertainment not reported.
Q3 EF measurement	Study	Yes	The validated Chinese FREE and current reliability (.73 enhancement; .80 suppression) are described.	p. 26125; Measures, Expressive flexibility; p. 26126, scoring/reliability continuation and Table 2.
Q4 Outcome measurement	Outcome	Yes	The Chinese STAI-T is identified as validated and has alpha .88 in this sample.	p. 26125; Measures, Trait anxiety.
Q5 Confounders identified	Study	No	For the extracted anxiety–EF correlation, no additional confounder set is identified. The focal ER mediators are not automatically confounders.	Not reported for the correlation; checked p. 26126, Statistical analysis and Table 2.
Q6 Confounding strategy	Outcome	No	The extracted coefficient is a zero-order correlation; no covariate-adjusted estimate of that correlation is supplied.	p. 26126; Descriptive analysis and Table 2 (zero-order correlations).
Q7 Statistical analysis	Result	Yes	Pearson correlations fit the continuous anxiety–ability association. The broader mediation models are explicitly atemporal.	p. 26126; Statistical analysis and Descriptive analysis.
Q8 Subjects and setting	Study	Yes	Chinese university recruitment through posters/online platforms, administration modes and demographic characteristics are described.	p. 26125; Participants and procedure.

S5 20 Southward and Cheavens (2017)

Analytical cross-sectional studies | Baseline depression and anxiety

Item	Level / exception	Judgment	Rationale	Evidence location
Q1 Eligibility	Study	Yes	Introductory-psychology students aged at least 18 were eligible.	p. 146; Methods and Materials, Participants.
Q2 Condition criteria	Outcome	N/A	No case definition was used for selection by this outcome; continuous symptom or well-being measurement is assessed under Q4.	pp. 148–149; Self-Report Measures, CES-D and STAI-S (continuous outcomes; review rule).
Q3 EF measurement	Study	Yes	The standardized expressive task uses three coders unaware of condition; ICCs are .82 for negative and .75 for positive emotion.	p. 147; Behavioral Task, coding, ICCs and EF scoring.
Q4 Outcome measurement	Outcome	Yes	CES-D and STAI-S are identified, with good internal consistency across assessments.	p. 148; Self-Report Measures, CES-D; p. 149, STAI-S.
Q5 Confounders identified	Study	Yes	Baseline symptoms are identified as controls in the longitudinal models; context and feedback sensitivity are also measured.	p. 150; Results, context/feedback-sensitivity moderation, baseline-symptom control; pp. 148–149, CFS-EC and EMBSS.
Q6 Confounding strategy	Outcome	No	The baseline CES-D/STAI associations extracted from Table 1 are zero-order correlations; baseline-outcome control belongs to the later models.	p. 151; Table 1 (baseline correlations); p. 150, prospective baseline adjustment.
Q7 Statistical analysis	Result	Yes	The reported correlation/regression procedures fit the continuous-score association question. Unreported diagnostics are not assumed to have been checked; cross-sectional associations do not establish causality.	p. 149; Analytic Strategy; p. 151, Table 1.
Q8 Subjects and setting	Study	Yes	The introductory-psychology sampling frame at a large Midwestern university, demographics and laboratory/online procedures are described.	p. 146; Participants; p. 149, Study Procedures.

Note. Table 1 uses the 138 longitudinal completers, including for baseline correlations; baseline associations are therefore also subject to selection into complete follow-up.

S5 20 Southward and Cheavens (2017)

Cohort studies | Later anxiety/depression composite

Item	Level / exception	Judgment	Rationale	Evidence location
Q1 Comparable groups	Study	N/A	No separately recruited exposed/unexposed groups: EF is a continuous predictor within one cohort. Q1 is not applicable to a two-group selection comparison; selection and attrition limitations are recorded elsewhere.	p. 146; Participants (single student cohort; review rule).
Q2 Consistent EF assessment	Study	Yes	Participants contributing to the comparison receive the same described EF measurement protocol; exposure ascertainment does not differ according to their score.	pp. 146–147; Behavioral Task, Expressive Flexibility Task.
Q3 EF measurement	Study	Yes	The expressive task uses independent condition-blind coders, with ICCs .82/.75; scoring is specified.	p. 147; Behavioral Task, coder blinding, ICCs and scoring.
Q4 Confounders identified	Study	Yes	Baseline symptoms, context sensitivity and feedback sensitivity are explicitly included.	p. 150; Results, context/feedback-sensitivity moderation and baseline-symptom control.
Q5 Confounding strategy	Outcome	Yes	Models control baseline anxiety/depression and examine context/feedback terms. Low reliability of the feedback measure (alpha .40) weakens inferences involving that variable.	p. 150; baseline-adjusted prospective model; p. 148, CFS-EC reliability.
Q6 Outcome absent initially	Outcome	N/A	The outcome is a continuous symptom/well-being score, not incident disease. Being outcome-free at baseline is not an eligibility requirement for this estimand; temporal ordering and baseline adjustment are stated separately. EF is assessed at baseline, before the two-week/one-month follow-ups.	p. 149; Study Procedures (EF at baseline, later symptoms; review rule).
Q7 Outcome measurement	Outcome	Yes	CES-D and STAI-S are validated and internally consistent; a standardized combined symptom outcome is transparently constructed.	p. 148; CES-D; p. 149, STAI-S and Analytic Strategy (composite).
Q8 Follow-up interval	Outcome	Yes	Two-week and one-month follow-ups are explicit and permit short-term symptom variation; no inference to long-term disorder incidence is warranted.	p. 149; Study Procedures, two-week and one-month follow-ups.
Q9 Follow-up completeness	Result	Unclear	13 of 151 participants lack complete follow-up (9%); measured-variable comparisons are reported, but specific reasons are not.	p. 146; Participants, 13 incomplete cases and retained/lost comparisons; reasons not reported.
Q10 Incomplete follow-up	Result	No	Complete-case analysis of 138 participants is reported, without a statistical strategy for missing follow-up outcomes.	Not reported; checked p. 146, Participants, and p. 149, Study Procedures and Analytic Strategy.
Q11 Statistical analysis	Result	Yes	Baseline-adjusted regressions and bootstrap mediation/moderation fit the stated association models. Poor feedback-measure reliability limits precision of those paths.	p. 149; Analytic Strategy; p. 150, baseline-adjusted models; p. 148, CFS-EC reliability.

Note. The feedback-sensitivity scale has low internal consistency; a Yes for the symptom-outcome measure does not endorse all other model measures.

S5 21 Strickland and Skolnick (2020)

Analytical cross-sectional studies | Trait anxiety

Item	Level / exception	Judgment	Rationale	Evidence location
Q1 Eligibility	Study	Yes	MTurk recruitment, English comprehension/validity checks, incomplete-response and outlier exclusions are described.	p. 2; Section 2.1, Participants.
Q2 Condition criteria	Outcome	N/A	No case definition was used for selection by this outcome; continuous symptom or well-being measurement is assessed under Q4.	p. 3; Section 2.2.3, Trait anxiety (continuous outcome; review rule).
Q3 EF measurement	Study	Yes	The FREE has alpha .84 overall and factor analyses by country. Some country-specific fit indices are modest, so cross-cultural equivalence is not treated as established.	p. 3; Sections 2.2.1, Expressive regulation ability, and 3.1, Confirmatory factor analyses.
Q4 Outcome measurement	Outcome	Yes	The standard STAI-T is used with alpha .93.	p. 3; Section 2.2.3, Trait anxiety.
Q5 Confounders identified	Study	Yes	Country, collectivistic values and habitual regulation strategies are explicitly examined.	p. 3; Sections 2.2.4, Cultural values, and 2.3, Statistical analyses.
Q6 Confounding strategy	Outcome	Yes	Partial correlations control country/cultural values; country-specific regressions include reappraisal and habitual suppression. Separate bivariate correlations remain unadjusted; the affirmative judgment applies to the reported outcome-level adjustment strategy.	p. 4; Sections 3.3, Zero-order correlation analysis, and 3.4, Regression analyses; Table 4.
Q7 Statistical analysis	Result	Yes	Correlation and regression methods fit the associational question; results with and without outliers are provided. Reported degrees of freedom/sample counts are not uniformly transparent.	p. 3; Section 2.3, Statistical analyses (including outlier checks); p. 4, Sections 3.3–3.4.
Q8 Subjects and setting	Study	Yes	MTurk, collection waves, country composition, exclusions and demographics are described.	p. 2; Section 2.1, Participants; p. 3, Section 2.2.4, country composition.

Note. Interpret cross-country differences cautiously: within-country factor fit is not uniformly strong and does not itself demonstrate measurement invariance.

S5 22 Wang et al. (2020) Experiment 1

Randomized controlled experiments | Peer acceptance of the observed target

Item	Level / exception	Judgment	Rationale	Evidence location
Q1 Randomization	Study	Unclear	Random assignment to three conditions is stated, but the sequence-generation method is not described.	p. 5; Experiment 1; p. 6, Preparation and instruction. Random assignment stated; sequence-generation method not reported.
Q2 Allocation concealment	Study	Unclear	Concealment of the forthcoming group allocation from those assigning participants is not reported.	Not reported; checked p. 6, Procedure, Preparation and instruction.
Q3 Baseline similarity	Study	Unclear	Condition sizes and sex proportions are similar; broader baseline comparability by condition is not documented.	p. 6; Preparation and instruction (group sizes and sex); p. 5, Participants (overall age only).
Q4 Participant blinding	Study	Unclear	A cover story is used, but blinding to the assigned video/instruction condition is not established. Participants directly observe the manipulated expressive behavior.	p. 6; Preparation and instruction and Video viewing (cover story and visible condition-specific stimuli).
Q5 Provider blinding	Study	Unclear	The report does not establish that the staff delivering the experiment were blind to condition.	Not reported; checked p. 6, Procedure, Preparation and instruction and Video viewing.
Q6 Other treatment equal	Study	Yes	Procedures, same-sex target matching and outcome timing are otherwise standardized; the presented expressive capacity differs as intended.	p. 6; Video viewing; p. 7, Liking measurement (participant-rated outcome).
Q7 Assessor blinding	Outcome	Unclear	Participants themselves rate liking. Blinding to their assigned exposure condition is not established, although the full study hypothesis may be concealed.	p. 6; Video viewing; p. 7, Liking measurement (common procedure).
Q8 Same outcome method	Outcome	Yes	All groups complete the same nine-item liking questionnaire after the video task.	p. 7; Liking measurement (identical scale across conditions).
Q9 Outcome reliability	Outcome	Yes	The adaptation of Rubin's Liking Scale is explained, items unsuitable for the context are removed and alpha is .79.	p. 7; Liking measurement (item adaptation and reliability).
Q10 Follow-up completeness	Result	Unclear	Six of 153 recruits are excluded for instruction/distraction problems. Their distribution across randomized groups and allocation timing are not sufficiently specified.	p. 5; Participants (six exclusions; exclusions by assigned group not reported).
Q11 Original-group analysis	Result	Unclear	The retained 147 are analyzed by assigned group; handling of all randomized participants cannot be verified because excluded cases are not traced by allocation.	p. 5; Participants; p. 6, Preparation and instruction; p. 8, Liking scores. Analysis of all originally assigned participants not established.
Q12 Statistical analysis	Result	Yes	Between-group ANOVA and reported contrasts fit the immediate liking comparison, with sex included. Estimates concern this standardized target-video task.	p. 8; Results and discussion, Liking scores (factorial ANOVA and comparisons).
Q13 Trial design	Study	Yes	A parallel three-condition experiment with standardized stimuli and randomized presentation order is appropriate for the immediate experimental question.	p. 5; Experiment 1; p. 6, Video viewing; p. 7, Table 1 (parallel conditions and common task).

Note. This experiment tests acceptance of a target displaying manipulated expressive ability, not an intervention that changes participants' own EF or clinical symptoms.

S5 23 Wang et al. (2022)

Analytical cross-sectional studies | Psychological distress at T2

Item	Level / exception	Judgment	Rationale	Evidence location
Q1 Eligibility	Study	Yes	Cisgender sexual-minority men recruited through Grindr in specified US geographic strata form the analytic population; completeness exclusions are stated.	p. 215; Method, Participants and Procedures.
Q2 Condition criteria	Outcome	N/A	No case definition was used for selection by this outcome; continuous symptom or well-being measurement is assessed under Q4.	p. 216; Measures, Psychological Distress (continuous outcome; review rule).
Q3 EF measurement	Study	Yes	The standard FREE, polarity-corrected score and enhancement/suppression reliability (.73/.81) are reported.	p. 216; Measures, Expressive Flexibility.
Q4 Outcome measurement	Outcome	Yes	The BSI-18 distress composite is justified by high subscale correlations and alpha .94.	p. 216; Measures, Psychological Distress.
Q5 Confounders identified	Study	Yes	Age, income, education and employment are identified as covariates.	p. 217; Data Analysis, demographic covariate-selection paragraph.
Q6 Confounding strategy	Outcome	Yes	T2 models control the specified demographic variables. Separate bivariate correlations remain unadjusted; the affirmative judgment applies to the reported outcome-level adjustment strategy.	p. 217; Data Analysis and Cross-Sectional Results; Table 2 (unadjusted correlations).
Q7 Statistical analysis	Result	Yes	The reported correlation/regression procedures fit the continuous-score association question. Unreported diagnostics are not assumed to have been checked; cross-sectional associations do not establish causality.	p. 217; Data Analysis, PROCESS models and interaction probing.
Q8 Subjects and setting	Study	Yes	Geographic sampling strata, platform, eligibility and detailed analytic-sample demographics are given.	pp. 215–216; Participants and Procedures; p. 216, Table 1.

Note. EF was measured at T2 only; the main EF–distress association is concurrent, even though the wider project is longitudinal.

S5 23 Wang et al. (2022)

Cohort studies | Distress at T2 in the supplementary longitudinal model

Item	Level / exception	Judgment	Rationale	Evidence location
Q1 Comparable groups	Study	N/A	No separately recruited exposed/unexposed groups: EF is a continuous predictor within one cohort. Q1 is not applicable to a two-group selection comparison; selection and attrition limitations are recorded elsewhere.	p. 215; Participants and Procedures (single cohort; review rule).
Q2 Consistent EF assessment	Study	Yes	Participants contributing to the comparison receive the same described EF measurement protocol; exposure ascertainment does not differ according to their score.	p. 216; Measures, Expressive Flexibility.
Q3 EF measurement	Study	Yes	The FREE is adequately described and reliable at T2. It was not measured at baseline.	p. 216; Expressive Flexibility; p. 215, EF measured only at follow-up.
Q4 Confounders identified	Study	Yes	Baseline distress and demographic variables are identified.	p. 217; Data Analysis, demographic and baseline-distress covariates.
Q5 Confounding strategy	Outcome	Yes	Models account for T1 distress, age, income, education and employment, but cannot control baseline EF because it was not measured.	p. 217; final Data Analysis paragraph; p. 219, Longitudinal Results.
Q6 Outcome absent initially	Outcome	N/A	The outcome is a continuous symptom/well-being score, not incident disease. Being outcome-free at baseline is not an eligibility requirement for this estimand; temporal ordering and baseline adjustment are stated separately. Crucially, EF and the reviewed outcome are both measured at T2: prospective ordering of EF before distress is not established.	p. 215; Participants and Procedures; p. 217, final Data Analysis paragraph (EF only at T2; review rule).
Q7 Outcome measurement	Outcome	Yes	BSI-18 scores have alpha .94 at both waves and consistent scoring.	p. 216; Measures, Psychological Distress (reliability at both waves).
Q8 Follow-up interval	Outcome	Unclear	Approximately one year separates T1 discrimination/distress and T2; there is no follow-up interval from EF measurement to the outcome. Q8 cannot affirm prospective EF–outcome timing.	p. 215; Participants and Procedures, one-year interval and EF timing.
Q9 Follow-up completeness	Result	Unclear	427 complete T2 and 377 enter the primary analytic sample; the original eligible T1 denominator and outcome-related loss are insufficiently reported for cohort retention.	pp. 215–216; Participants and Procedures, 427 follow-up respondents and 377 analytic cases; eligible baseline denominator not provided.
Q10 Incomplete follow-up	Result	No	Little’s MCAR test and listwise deletion concern missing available data; no strategy addressing loss from the original cohort is established.	pp. 216–217; Data Analysis, MCAR and listwise deletion; original-cohort attrition strategy not reported.
Q11 Statistical analysis	Result	Yes	The model is suitable for a baseline-distress-adjusted association with concurrent EF. It does not support a claim that earlier EF prospectively predicts later distress.	p. 217; final Data Analysis paragraph; p. 219, Longitudinal Results.

Note. The longitudinal analysis is prospective for T1 discrimination, but not for EF, which was measured at T2 only.

S5 24 Wang et al. (2023)

Analytical cross-sectional studies | Concurrent friendship quality and social anxiety

Item	Level / exception	Judgment	Rationale	Evidence location
Q1 Eligibility	Study	Yes	The junior-high cohort, grades and availability of EF measures at all waves define selection; exclusions for school change/withdrawal and random responding are reported.	p. 416; Section 2.1, Participants, including footnote 1.
Q2 Condition criteria	Outcome	N/A	No case definition was used for selection by this outcome; continuous symptom or well-being measurement is assessed under Q4.	pp. 416–417; Sections 2.2.2–2.2.3 (continuous outcomes; review rule).
Q3 EF measurement	Study	Yes	CAFE was developed for Chinese youth; scoring and repeated-wave reliability are provided.	p. 416; Section 2.2.1, Expressive flexibility.
Q4 Outcome measurement	Outcome	Yes	Validated Chinese friendship-quality and social-anxiety measures are used. A poorly performing friendship subdimension is transparently removed, with good reliability and factor support for the retained score.	p. 416; Section 2.2.2, Friendship quality; p. 417, Section 2.2.3, Social anxiety.
Q5 Confounders identified	Study	Yes	Age and sex are identified as controls for the longitudinal model.	p. 417; Section 2.4, Data analyses, age/sex controls.
Q6 Confounding strategy	Outcome	No	The concurrent associations extracted from the correlation table are unadjusted. Age/sex control in the panel model does not adjust these bivariate coefficients.	p. 417; Section 2.4; p. 418, Table 1 (unadjusted concurrent correlations).
Q7 Statistical analysis	Result	Unclear	The reported correlation/regression procedures fit the continuous-score association question. Unreported diagnostics are not assumed to have been checked; cross-sectional associations do not establish causality. The impact of unmodeled school clustering on standard errors cannot be determined; this specific independence concern motivates Unclear.	p. 417; Section 2.4; p. 416, Section 2.1 (two schools); school clustering not addressed in analysis specification.
Q8 Subjects and setting	Study	Yes	Two Shandong schools, grades, ages, sex composition and classroom assessment procedures are described.	p. 416; Section 2.1; p. 417, Section 2.3, Procedure.

Note. Two schools contribute the sample, but clustering is not addressed. The Unclear statistical judgment concerns independence/standard errors, not proof of a biased association.

S5 24 Wang et al. (2023)

Cohort studies | Later friendship quality and social anxiety

Item	Level / exception	Judgment	Rationale	Evidence location
Q1 Comparable groups	Study	N/A	No separately recruited exposed/unexposed groups: EF is a continuous predictor within one cohort. Q1 is not applicable to a two-group selection comparison; selection and attrition limitations are recorded elsewhere.	p. 416; Section 2.1, Participants (single cohort; review rule).
Q2 Consistent EF assessment	Study	Yes	Participants contributing to the comparison receive the same described EF measurement protocol; exposure ascertainment does not differ according to their score.	p. 416; Section 2.2.1, Expressive flexibility, identical scale across waves.
Q3 EF measurement	Study	Yes	CAFE is developed for Chinese youth with repeat-wave reliability and consistent scoring.	p. 416; Section 2.2.1, scoring and repeated-wave reliability.
Q4 Confounders identified	Study	Yes	Age, sex and prior levels of the modeled variables are identified.	p. 417; Section 2.4, Data analyses.
Q5 Confounding strategy	Outcome	Yes	The panel model includes age/sex, autoregressive stability and reciprocal cross-lagged paths.	p. 417; Section 2.4, stability/cross-lagged paths and age/sex controls; Figure 1.
Q6 Outcome absent initially	Outcome	N/A	The outcome is a continuous symptom/well-being score, not incident disease. Being outcome-free at baseline is not an eligibility requirement for this estimand; temporal ordering and baseline adjustment are stated separately. EF is measured before the later outcomes and earlier levels of each modeled outcome are included.	p. 416; Section 2.1, wave timing; p. 417, Section 2.4, prior-level paths (review rule).
Q7 Outcome measurement	Outcome	Yes	Validated Chinese measures are used; the retained friendship score has alpha .93–.94 and longitudinal factor support, and social anxiety alpha .73–.79.	p. 416; Section 2.2.2; p. 417, Section 2.2.3; p. 418, Section 3.2, measurement invariance.
Q8 Follow-up interval	Outcome	Yes	Six- and twelve-month measurements provide explicit intervals suitable for changes in friendship and social anxiety.	p. 416; Section 2.1, six- and twelve-month waves.
Q9 Follow-up completeness	Result	Yes	274 of 287 participants are retained (95.5%). School change/withdrawal and one random-response exclusion are described; the limited loss supports Yes while not proving absence of selection.	p. 416; Section 2.1, 287 initial and 274 retained participants, reasons for exclusions.
Q10 Incomplete follow-up	Result	No	The analysis is restricted to retained valid participants; no additional missing-outcome strategy is reported. The small loss mitigates impact but does not itself constitute a strategy.	Not reported; checked p. 416, Section 2.1, and pp. 417–418, Section 2.4, Data analyses.
Q11 Statistical analysis	Result	Unclear	The cross-lagged model includes stability and reciprocal paths and reports fit. It estimates panel associations, not necessarily within-person causal effects; two-school clustering is not modeled. The impact of unmodeled school clustering on standard errors cannot be determined; this specific independence concern motivates Unclear.	pp. 417–418; Section 2.4, Data analyses; p. 420, Section 3.3 continuation (model fit); school clustering not addressed.

Note. Two schools contribute the sample, but clustering is not addressed. The Unclear statistical judgment concerns independence/standard errors, not proof of a biased association.

S5 25 Wang et al. (2026)

Analytical cross-sectional studies | Drug-related problems

Item	Level / exception	Judgment	Rationale	Evidence location
Q1 Eligibility	Study	Yes	Detailed MDD, recent substance-use, age, language, location and clinical-exclusion criteria are stated.	p. 3; Method, Participants and Procedure, Participants.
Q2 Condition criteria	Outcome	Yes	A structured MINI interview administered by trained staff confirms MDD and assesses comorbidity.	p. 3; Screening and Study Procedure; Measures, MINI.
Q3 EF measurement	Study	Yes	The standard FREE and polarity-corrected EF score are described, with alpha .81.	p. 4; Measures, Expressive Flexibility; p. 5, continuation (reliability).
Q4 Outcome measurement	Outcome	Yes	The validated 15-item Short Inventory of Problems for Drug Use is described; alpha is .96.	p. 5; Measures, Drug Use Problems.
Q5 Confounders identified	Study	Yes	Gender, race and depressive-symptom severity are identified and justified as covariates.	p. 5; Covariates and Data Analysis (covariate rationale).
Q6 Confounding strategy	Outcome	Yes	Drug-problem models adjust for gender, race and depression. Separate bivariate correlations remain unadjusted; the affirmative judgment applies to the reported outcome-level adjustment strategy.	p. 5; Data Analysis (adjusted PROCESS models).
Q7 Statistical analysis	Result	Yes	The skewed drug-problem score is square-root transformed before regression/moderation; the model fits the associational question. Bootstrapping does not remove all regression assumptions.	p. 5; Data Analysis; Results, first paragraph (distribution checks and transformation).
Q8 Subjects and setting	Study	Yes	New Haven recruitment channels, February 2020–January 2024 dates, screening, diagnostic procedures and final analytic sample are reported.	p. 3; Recruitment and Analytic Sample; p. 4, Table 1.

S5 26 Westphal et al. (2010)

Analytical cross-sectional studies | Friend-rated psychological adjustment

Item	Level / exception	Judgment	Rationale	Evidence location
Q1 Eligibility	Study	No	Fourth-year college status and study participation are described; initial cohort eligibility and selection into the 50-person outcome subsample are not fully specified.	Not reported; checked p. 94, Method, Participants and Procedures (sample described but initial eligibility unspecified).
Q2 Condition criteria	Outcome	N/A	No case definition was used for selection by this outcome; continuous symptom or well-being measurement is assessed under Q4.	p. 95; Friend Ratings of Adjustment (continuous adjustment outcome; review rule).
Q3 EF measurement	Study	Yes	The expressive paradigm, randomized block order, observer ratings and manipulation checks support measurement; the balanced EF score is specified.	p. 94; Expressive Regulation Paradigm; p. 95, Manipulation Check.
Q4 Outcome measurement	Outcome	Yes	Two friends rate multiple adjustment domains using an established approach; the composite alpha is .90. Participant nomination may still influence informant selection.	p. 95; Friend Ratings of Adjustment; p. 94, Participants and Procedures (friend-rating subsample).
Q5 Confounders identified	Study	Yes	Baseline expressiveness is explicitly identified as a possible alternative explanation arising from floor/ceiling effects, in addition to the contextual variables.	p. 97; Alternative explanation (baseline expressiveness).
Q6 Confounding strategy	Outcome	Yes	The authors repeat the EF regression controlling baseline expressiveness; component models include the complementary ability. This supports a limited, explicitly stated adjustment strategy.	p. 97; Alternative explanation (adjusted reanalyses); pp. 95–96, Expressive enhancement and suppression (mutual adjustment).
Q7 Statistical analysis	Result	Yes	Interaction regression fits the contextual association question; the small 50-person subsample limits precision.	pp. 95–96; Results, threat-context/cumulative-stress moderation analyses; p. 96, Table 1.
Q8 Subjects and setting	Study	No	Study year and procedures are given, but the current recruitment setting/frame and rationale for outcome-subset selection are not detailed enough.	p. 94; Participants and Procedures (initial cohort and later subsamples).

Note. The friend-adjustment association concerns the fourth-year assessment. The three-year retest demonstrates stability of EF, not prospective prediction of this health outcome.

S5 27 Zhang et al. (2023)

Analytical cross-sectional studies | Depressive symptoms

Item	Level / exception	Judgment	Rationale	Evidence location
Q1 Eligibility	Study	No	Six-school recruitment and achieved ages/ethnicity are described, but explicit eligibility criteria are not separately specified.	Not reported; checked p. 3, Section 2.1, Participants (recruitment described, eligibility not specified).
Q2 Condition criteria	Outcome	N/A	No case definition was used for selection by this outcome; continuous symptom or well-being measurement is assessed under Q4.	pp. 3–4; Section 2.2.2, Depressive symptoms (continuous CES-D; review rule).
Q3 EF measurement	Study	Yes	The Chinese-youth AFREE is referenced as validated; total/enhancement/suppression alpha values are .87/.83/.85.	p. 3; Section 2.2.1, Expressive flexibility.
Q4 Outcome measurement	Outcome	Yes	The Chinese CES-D and scoring are described, with alpha .83.	pp. 3–4; Section 2.2.2, Depressive symptoms (instrument and reliability).
Q5 Confounders identified	Study	Yes	Age and gender are identified as control variables.	p. 4; Section 2.3, Statistical analyses (age and gender).
Q6 Confounding strategy	Outcome	Yes	Depression regressions include age and gender. Separate bivariate correlations remain unadjusted; the affirmative judgment applies to the reported outcome-level adjustment strategy.	p. 4; Section 2.3 (adjusted regressions); Table 1 (separate unadjusted correlations).
Q7 Statistical analysis	Result	Unclear	Linear regressions fit the continuous association. Students are sampled from six schools, but clustering is not addressed; its impact on standard errors cannot be determined.	p. 4; Section 2.3; p. 3, Section 2.1 (six schools; clustering not addressed in analysis specification).
Q8 Subjects and setting	Study	Yes	School count, three provinces, age distribution, ethnicity, sex composition and consent arrangements are reported.	p. 3; Section 2.1, Participants.

Note. The Unclear judgment at Q7 reflects unresolved handling of school clustering; this does not establish that the coefficient itself is biased.

S5 28 Zhao and Wang (2024)

Analytical cross-sectional studies | Depression, anxiety, stress and relationship stress

Item	Level / exception	Judgment	Rationale	Evidence location
Q1 Eligibility	Study	No	Undergraduate classroom recruitment and sample characteristics are reported; explicit initial inclusion/exclusion criteria are not supplied.	Not reported; checked p. 4, Section 2.1, Participants and Procedure (initial eligibility unspecified).
Q2 Condition criteria	Outcome	N/A	No case definition was used for selection by this outcome; continuous symptom or well-being measurement is assessed under Q4.	p. 5; Sections 2.2.3–2.2.4 (continuous outcomes; review rule).
Q3 EF measurement	Study	Unclear	Chinese FREE factor analyses and composite reliability support the enhancement/suppression measures. A negative-suppression subscale has lower reliability; the paper's unexplained reference to reverse-scoring needs clarification.	p. 4; Section 2.2.1, FREE; p. 6, Table 1 and Section 3.2, Factor Analysis.
Q4 Outcome measurement	Outcome	Yes	Chinese DASS-21 and the interpersonal subscale of the College Stress Scale are described with current reliability (.76–.79 and .84).	p. 5; Sections 2.2.3, Symptoms of Depressive, Anxiety, and Stress, and 2.2.4, College Students Stress.
Q5 Confounders identified	Study	Yes	Age, gender and emotion-regulation self-efficacy are explicitly considered.	p. 5; Section 2.3, Data Analyses (age, gender and CRESE).
Q6 Confounding strategy	Outcome	Yes	All health/relationship-stress regression models include the specified covariates. Separate bivariate correlations remain unadjusted; the affirmative judgment applies to the reported outcome-level adjustment strategy.	p. 5; Section 2.3 (covariates in all regressions); p. 6, Table 1 (unadjusted correlations).
Q7 Statistical analysis	Result	Yes	Hierarchical regression and interaction tests fit the association question. Missing-data procedures and factor structure are discussed.	p. 5; Sections 2.3, Data Analyses, and 3.1, Descriptive Statistics (missing-data procedure).
Q8 Subjects and setting	Study	Yes	Recruitment from two mainland-Chinese universities during psychology classes, administration and demographic characteristics are described.	p. 4; Section 2.1, Participants and Procedure.

Note. The statement that FREE items were reverse-scored is unexplained. This scoring ambiguity underlies Q3 Unclear despite supportive composite psychometrics.

S5 29 Zhu and Bonanno (2017)

Analytical cross-sectional studies | Baseline depression in relation to within-session EF change

Item	Level / exception	Judgment	Rationale	Evidence location
Q1 Eligibility	Study	Yes	The authors explicitly state no recruitment exclusions, describe advertisements and enumerate the three post-recruitment exclusions.	p. 4; Method, Participants (recruitment and exclusions).
Q2 Condition criteria	Outcome	N/A	No case definition was used for selection by this outcome; continuous symptom or well-being measurement is assessed under Q4.	p. 4; Questionnaires, Depression level (continuous CES-D; review rule).
Q3 EF measurement	Study	Yes	The expressive task is standardized; four independent observers provide averaged ratings with ICC .84. EF change is distinguished from first-phase ability.	p. 5; EF task and Data reduction and analysis, EF task (four-rater ICC); pp. 8–9, EF change-score analyses.
Q4 Outcome measurement	Outcome	Yes	The validated 11-item CES-D short form is identified and has alpha .86.	p. 4; Questionnaires, Depression level.
Q5 Confounders identified	Study	Yes	Age, gender, race/ethnicity, task order, complementary affective abilities and feedback assignment are considered as alternative explanations.	p. 6; Results, first paragraph (demographic/task-order screening); p. 9, Table 2 (AF change and group).
Q6 Confounding strategy	Outcome	Yes	Hierarchical models include both expressive changes, affective changes and feedback terms; demographic/task-order effects were screened and omitted. This strategy leaves residual confounding possible.	p. 6; Results, first paragraph; p. 9, Table 2 and accompanying regression description.
Q7 Statistical analysis	Result	Yes	Regression of baseline depression on within-session ability-change measures answers an association question. Because depression precedes the task, it cannot demonstrate that task improvement reduced depression.	p. 4; Procedure (depression measured before tasks); p. 9, Table 2 and accompanying regression description.
Q8 Subjects and setting	Study	Yes	Community advertising, compensation, age/sex/ethnic composition and laboratory procedures are described, although socioeconomic status is not assessed.	p. 4; Participants and Procedure (community recruitment, exclusions and demographics).

Note. The randomized feedback manipulation does not make the reviewed baseline-depression association an intervention effect; that association receives a cross-sectional appraisal.
