# Supplementary material for: Emotional Expressive Flexibility and Mental Health: A Systematic Review and Meta-Analysis
Source: Brain Sci. 2026 Sep 29;16(10):1041. doi: 10.3390/brainsci16101041 (PMC13702306; doi:10.3390/brainsci16101041)
Supplement: Supplementary file 1 [file brainsci-16-01041-s001.zip › Supplementary_File_S3_Funnel_Plots_Review_ExpressiveFlexibility.pdf]

Supplementary File S3

Funnel Plots for the Meta-Analyses

Emotional Expressive Flexibility and Mental Health: A Systematic Review and Meta-Analysis

Figure S1. Funnel plot for the association between overall expressive flexibility and depressive symptoms.

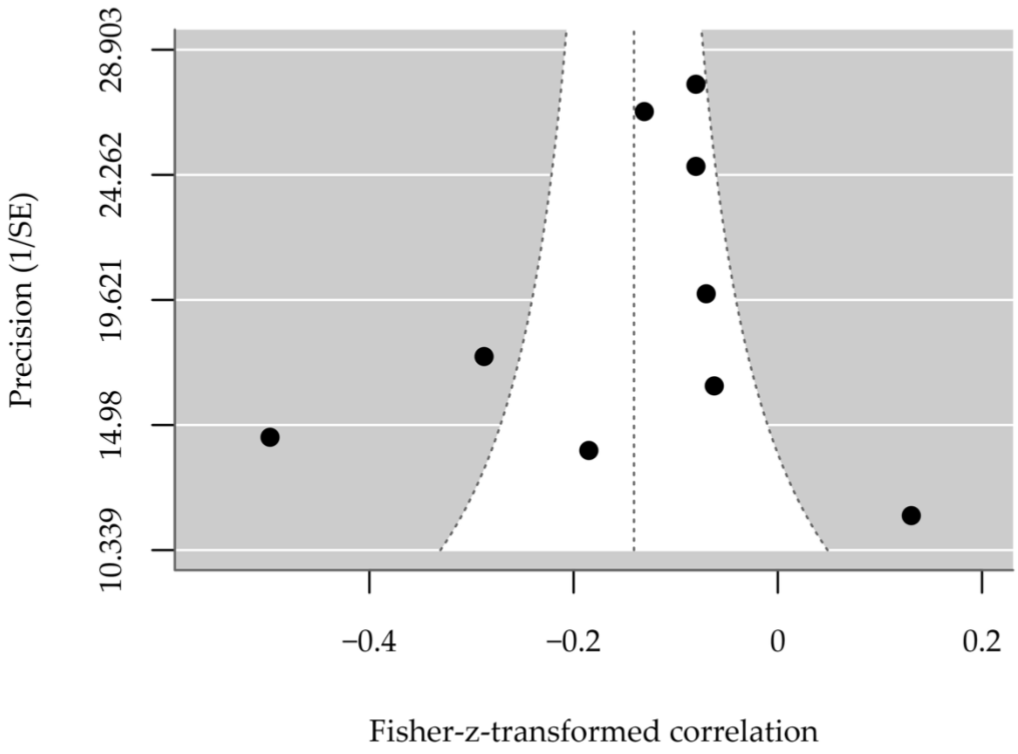

Note. Each point represents one independent study-level effect included in the primary meta-analysis ($k = 9$). The vertical dotted line indicates the pooled Fisher's z-transformed correlation, and the diagonal dotted lines indicate the approximate 95% pseudo-confidence limits. Because fewer than 10 independent effects were available, the funnel plot is presented for descriptive purposes only.

Figure S2. Funnel plot for the association between overall expressive flexibility and anxiety.

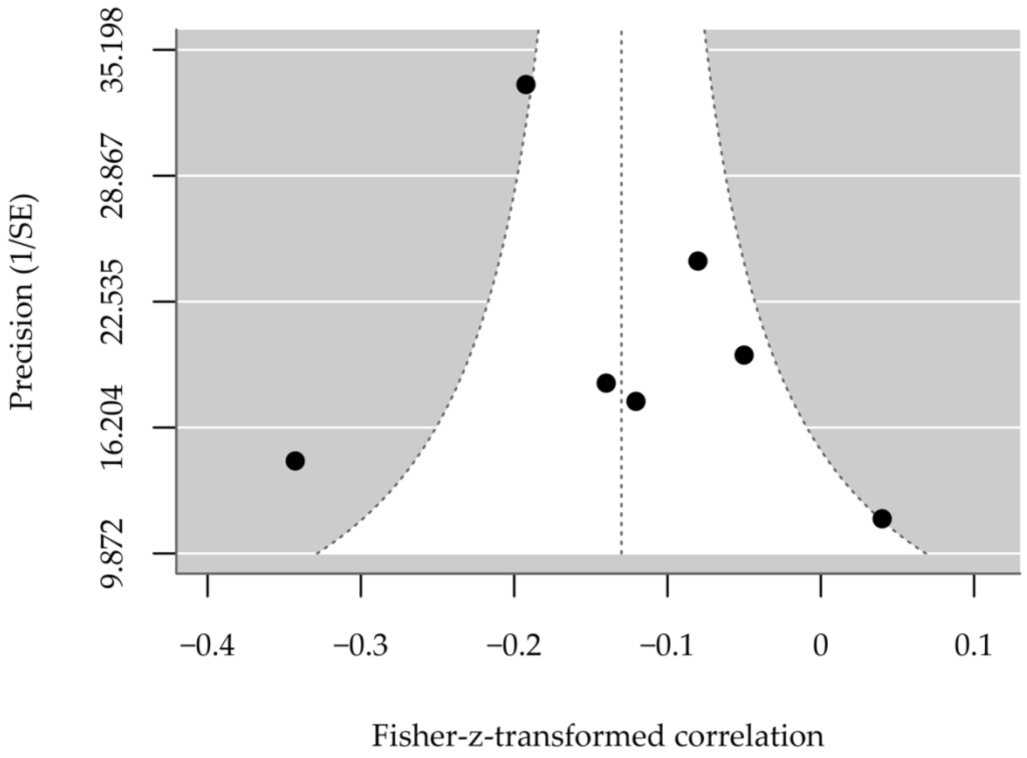

Note. Each point represents one independent study-level effect included in the exploratory meta-analysis (k = 7). The vertical dotted line indicates the pooled Fisher's z-transformed correlation, and the diagonal dotted lines indicate the approximate 95% pseudo-confidence limits. Because fewer than 10 independent effects were available, the funnel plot is presented for descriptive purposes only.
