# Supplementary material for: Emotional Expressive Flexibility and Mental Health: A Systematic Review and Meta-Analysis
Source: Brain Sci. 2026 Sep 29;16(10):1041. doi: 10.3390/brainsci16101041 (PMC13702306; doi:10.3390/brainsci16101041)
Supplement: Supplementary file 1 [file brainsci-16-01041-s001.zip › Supplementary_File_S4_Outcome_Level_Appraisal_Review_ExpressiveFlexibility.pdf]

Outcome-level appraisal of the evidence

Supplementary material for the systematic review of expressive flexibility

Purpose and scope

This structured narrative appraisal evaluates limitations of the evidence for each outcome, integrating the study-level JBI appraisals with the narrative and quantitative syntheses. It was developed during revision. It is not a formal GRADE assessment, assigns no categorical certainty ratings, and does not replace the design-specific JBI appraisals.

The target of inference is an association between expressive flexibility and the specified outcome. Concurrent associations, prospective findings and experimental contrasts are distinguished. The three pooled estimates concern overall expressive flexibility; component-specific and valence-specific findings are not treated as interchangeable with these estimates.

Assessment rules

Risk of bias. Use the JBI items relevant to the particular outcome and analysis. An affirmative confounding judgment for an adjusted model does not apply to a separate unadjusted correlation. Reporting uncertainty is distinguished from a demonstrated methodological failure.

Consistency. Consider effect direction, magnitude, measures, populations and temporal relationships. Do not count significant results as votes. A single study provides no assessment of between-study consistency; multiple analyses of one sample are not independent replications.

Precision and robustness. For pooled outcomes, examine confidence intervals, study numbers and sensitivity analyses. Prediction intervals address between-setting variation and are especially uncertain with few studies. No clinical-importance threshold was prespecified; neither nonsignificance nor proximity to zero establishes equivalence. For unpooled outcomes, the absence of a pooled interval is reported without assigning an aggregate precision judgment unsupported by a comparable synthesis.

Applicability. Relate each finding to its actual population, measure and design. Non-clinical evidence directly informs non-clinical associations; extrapolation to psychiatric diagnoses, demonstrated performance or intervention effects requires separate support.

Publication and reporting bias. Apply the shared assessment below to every outcome, adding outcome-specific reporting gaps. Inability to test bias is not evidence that bias is absent.

Conclusion. State the supported association and the unresolved inference in words. No scores are summed and no GRADE categories are assigned. Related limitations are described without numerical double-counting.

Methodological context

The domains draw on general evidence-appraisal considerations described in the Cochrane Handbook, Chapter 14 (https://www.cochrane.org/authors/handbooks-and-manuals/handbook/current/chapter-14). Their use here does not constitute implementation of GRADE. PRISMA 2020 items 15 and 22 concern reporting of methods and findings, respectively (Page et al., 2021; doi:10.1136/bmj.n71).

Shared limitations and quantitative overview

Publication and reporting bias remain unresolved for all outcomes. Eligibility was restricted to English-language, peer-reviewed reports; grey literature was excluded. The three pooled models contain fewer than ten independent study effects, so formal small-study-effect tests were not performed. Descriptive funnel plots cannot establish the absence of bias. The remaining outcomes have sparse and heterogeneous evidence that does not support a reliable quantitative assessment of publication bias.

The three main models pool concurrent, unadjusted Pearson correlations from non-clinical samples. Shared self-report measurement may contribute to associations: EF was self-reported in eight of nine depression studies, six of seven anxiety studies, and all four life-satisfaction studies. This concern does not invalidate self-report measurement, but limits claims about independently observed expressive performance. Outcome/model choices were made after extraction and multiplicity was not adjusted across the three syntheses; anxiety is exploratory.

Outcome	Studies / *N*	Pooled *r* [95% CI]	95% prediction interval	I^2 / leave-one-out
Depressive symptoms	9 / 3,622	−.14 [−.26, −.01]	[−.47, .23]	90%; 5/9 CIs include 0
Life satisfaction	4 / 1,910	.27 [.02, .49]	[−.28, .69]	92%; 4/4 CIs include 0
Anxiety (exploratory)	7 / 3,134	−.13 [−.23, −.03]	[−.35, .11]	76%; 2/7 CIs include 0

CI = confidence interval. Leave-one-out counts refer to intervals including zero, not reversals of the estimated direction. Estimates are rounded; *N* is outcome-specific and must not be summed across models because samples overlap. The prediction interval for life satisfaction is particularly uncertain with only four studies.

Traceability

Quantitative values are those reported in manuscript Sections 3.5.1–3.5.3 and the corresponding primary-model, prediction-interval, leave-one-out and sensitivity outputs. Study identifiers below use author and year, independently of reference numbering. JBI source judgments and published-page locators are in Section S5 of the companion supplement "JBI critical appraisal of expressive flexibility studies." Narrative outcome membership and findings correspond to Sections 3.4.1–3.4.4 and the accompanying outcome-extraction dataset. Shared limitations on this page apply to every following profile.

For Bonanno et al. (2004), the appraisal concerns psychological distress at follow-up, the outcome used to examine longer-term adjustment in the manuscript. Westphal et al. (2010) is evaluated separately as a concurrent association with friend-rated adjustment in a partly overlapping cohort. Wang et al. (2020), Experiment 2, is contextual evidence about social exclusion affecting expression and is not treated as evidence that EF improves a mental-health outcome.

Appraisal of the pooled outcomes

Depressive symptoms

Evidence and risk of bias. Nine-study pooled model. In addition to the shared unadjusted/self-report limitations, JBI flags unspecified depression measurement in Burton and Bonanno (2016), EF-measurement uncertainty in Lenzo et al. (2021) and Zhao and Wang (2024), and school clustering in Zhang et al. (2023).

Consistency, precision and robustness. The mean is inverse, but its CI extends close to zero and I^2 is 90%. Leave-one-out estimates remain negative; five intervals include zero. Excluding influential Lenzo reduces r to −.10 and I^2 to 73%. Alternative coefficient/timepoint models remain inverse, while their prediction intervals still include zero.

Interpretation. The evidence supports a small average inverse concurrent association in the sampled non-clinical populations, with uncertain magnitude and between-setting generalizability. It does not establish prospective symptom reduction or a treatment effect. Additional narrative clinical, component and prospective findings do not remove these limitations.

Sources. Pooled: Lenzo (2021); Chen (2018); Burton and Bonanno (2016), Study 1; Shangguan (2022); Zhang (2023); Zhao and Wang (2024); Nardelli (2024); Ang and Tsai (2023); Southward and Cheavens (2017). Additional narrative evidence: Gonzalez-Escamilla (2022), Pițur and Miu (2020), Maccallum (2021), Dwyer (2024), Rodin (2017), and Zhu and Bonanno (2017). See JBI S5 and manuscript 3.4.1/3.5.1.

Life satisfaction

Evidence and risk of bias. Four-study model, all using the SWLS and self-reported EF. Adjusted analyses in some source studies do not control confounding in the pooled correlations. Consistent outcome measurement aids comparability, but common method and population selection remain relevant.

Consistency, precision and robustness. The CI spans approximately .02 to .49, I^2 is 92%, and all four leave-one-out CIs include zero, although estimates remain positive. Both Ang studies are influential. The prediction interval is very uncertain. Ang (2023) does not show a general prospective overall-flexibility association after baseline adjustment.

Interpretation. Preliminary support for a positive concurrent association; its magnitude and generalizability are uncertain. The evidence does not establish that higher EF produces later gains in life satisfaction.

Primary sources. Ang and Tsai (2023, 2025); Chen (2018); Shangguan (2022). See JBI S5 and manuscript 3.4.2/3.5.2.

Anxiety

Evidence and risk of bias. Seven-study exploratory model combining trait, state and symptom measures. All correlations are concurrent and unadjusted. EF measurement is uncertain in Lenzo (2021) and Zhao (2024); Shangguan (2024) has unclear condition ascertainment.

Consistency, precision and robustness. I^2 is 76%; two leave-one-out CIs include zero, with negative point estimates throughout. The state/symptom-only model gives r = −.11, 95% CI [−.27, .06]. Replacing Southward's baseline coefficient with the prospective coefficient retains an inverse mean, but does not turn the synthesis into a longitudinal analysis.

Interpretation. Exploratory support for a small inverse average association; magnitude and consistency across anxiety constructs are uncertain. Social anxiety, diagnosed anxiety disorders and causal improvement require separate evidence.

Sources. Pooled: Lenzo (2021); Shangguan (2024); Strickland and Skolnick (2020); Chen (2018); Zhao and Wang (2024); Nardelli (2024); Southward and Cheavens (2017). Additional narrative evidence: Gonzalez-Escamilla (2022), Pițur and Miu (2020), Maccallum (2021), and Dwyer (2024). Social anxiety is appraised separately. See JBI S5 and manuscript 3.4.1/3.5.3.

Appraisal of outcomes synthesized narratively

Stress symptoms

Evidence. Six cross-sectional studies; several inverse associations, with differences between overall flexibility, components and adjusted models.

Risk of bias. Unadjusted findings remain vulnerable to confounding; JBI identifies uncertainty about EF measurement in Lenzo and Zhao. Dwyer reports no individual coefficients.

Consistency and precision. Findings vary; no pooled magnitude or precision estimate is available. Nonsignificant corrected results do not establish absence of association.

Applicability. Different EF methods and populations, including harmful alcohol use, limit a single general conclusion.

Interpretation. Some support for inverse concurrent associations, particularly involving suppression; no established prospective or causal relationship.

Sources. Gonzalez-Escamilla et al. (2022); Dwyer et al. (2024); Lenzo et al. (2021); Maccallum et al. (2021); Nardelli et al. (2024); Zhao and Wang (2024). JBI S5; manuscript 3.4. Shared publication/reporting-bias limitations apply.

Posttraumatic stress outcomes

Evidence. Two cross-sectional studies link enhancement ability to fewer PTSD symptoms. Only Levy-Gigi assesses overall flexibility and its interaction with exposure.

Risk of bias. Levy-Gigi has unresolved measurement/condition-ascertainment items and no confounding strategy; Rodin includes adjusted PTSD analyses.

Consistency and precision. Convergent enhancement findings come from only two studies; overall-flexibility findings lack independent replication within this outcome.

Applicability. Firefighters and combat veterans provide occupational/trauma-specific evidence.

Interpretation. Enhancement ability merits investigation; neither a general protective effect nor diagnostic utility is established.

Sources. Levy-Gigi et al. (2020); Rodin et al. (2017). JBI S5; manuscript 3.4. Shared publication/reporting-bias limitations apply.

General psychological distress

Evidence. Bonanno reports lower later distress after baseline adjustment. Wang reports concurrent EF–distress associations at T2; Nardelli reports no clear corrected association.

Risk of bias. Bonanno has unclear outcome-measure documentation and follow-up limitations. Wang measures EF only at T2; baseline distress adjustment does not establish EF precedence.

Consistency and precision. Measures and temporal estimands differ; no common pooled estimate. Bonanno is also discussed under long-term adjustment in the manuscript.

Applicability. College transition/September 11 exposure, sexual minority men, and online adults are distinct settings.

Interpretation. Prospective support is specific to Bonanno; the broader distress evidence cannot be treated as replicated longitudinal prediction.

Sources. Bonanno et al. (2004); Wang et al. (2022); Nardelli et al. (2024). JBI S5; manuscript 3.4. Shared publication/reporting-bias limitations apply.

Appraisal of outcomes synthesized narratively

Combined anxiety and depression

Evidence. One-month symptom reductions were conditionally associated with EF at lower context sensitivity and indirectly through feedback sensitivity.

Risk of bias. Baseline symptoms were controlled; follow-up completeness was unclear and missing-follow-up strategies received No in JBI.

Consistency and precision. One study; interaction and mediation findings are not independent replications or evidence of an unconditional effect.

Applicability. Undergraduates and a combined symptom endpoint; results cannot be assigned separately to anxiety and depression.

Interpretation. A conditional prospective hypothesis is supported for further testing; a general symptom-reduction effect is not established.

Sources. Southward and Cheavens (2017). JBI S5; manuscript 3.4. Shared publication/reporting-bias limitations apply.

Complicated grief

Evidence. One cross-sectional study found lower overall flexibility in the complicated-grief group than in asymptomatic bereaved and married control groups.

Risk of bias. JBI supports the reported measurement and adjustment procedures; observational group differences remain open to residual confounding and reverse direction.

Consistency and precision. No independent replication or pooled precision estimate.

Applicability. Bereavement groups and laboratory performance; not evidence about treatment response.

Interpretation. A disorder-relevant group difference is suggested, without establishing causation or diagnostic classification accuracy.

Sources. Gupta and Bonanno (2011). JBI S5; manuscript 3.4. Shared publication/reporting-bias limitations apply.

Borderline symptoms

Evidence. No clear corrected associations with overall flexibility or either component in one cross-sectional study.

Risk of bias. No confounding strategy for the reviewed correlations; self-report exposure and outcome share a measurement method.

Consistency and precision. One study; nonsignificance is not evidence of equivalence or absence of a meaningful association.

Applicability. Online community adults; generalization to diagnosed borderline personality disorder is untested.

Interpretation. Evidence is insufficient to establish either a reliable association or its absence.

Sources. Nardelli et al. (2024). JBI S5; manuscript 3.4. Shared publication/reporting-bias limitations apply.

Appraisal of outcomes synthesized narratively

Drug-related problems

Evidence. An inverse bivariate association was reported. In a moderation model adjusted for depressive symptoms, race and gender, expressive flexibility attenuated the associations of aggregated and internalized stigma with drug-related problems, but not those of enacted or anticipated stigma.

Risk of bias. JBI supports measurement and the stated adjustment strategy; cross-sectional moderation does not establish a protective causal process.

Consistency and precision. One study with several related analyses; no independent replication or common pooled effect.

Applicability. Adults with major depression and recent heavy substance use; applicability is specific to this selected sample.

Interpretation. The findings motivate tests of stigma-dependent associations; an independent association after covariate adjustment was not tested separately, and no treatment effect is established.

Sources. Wang et al. (2026). JBI S5; manuscript 3.4. Shared publication/reporting-bias limitations apply.

Social anxiety

Evidence. No concurrent association or prospective prediction by overall flexibility in a three-wave adolescent study.

Risk of bias. JBI flags unmodeled school clustering and absence of strategies for incomplete follow-up; prospective models include prior levels.

Consistency and precision. One study; null findings do not demonstrate equivalence. Social anxiety was not included in the broader anxiety meta-analysis.

Applicability. Chinese adolescents assessed with CAFE; not interchangeable with adult trait/state anxiety.

Interpretation. The available evidence does not establish a social-anxiety association; its absence is also not established.

Sources. Wang et al. (2023). JBI S5; manuscript 3.4. Shared publication/reporting-bias limitations apply.

Positive affect

Evidence. Overall flexibility and both components were positively associated with positive affect; some associations differed by age and gender.

Risk of bias. JBI supports measurement; the overall and component associations are unadjusted bivariate correlations, with age and gender examined as moderators. Concurrent self-reports allow shared-method and reverse-direction explanations.

Consistency and precision. One study; subgroup analyses do not provide independent replication.

Applicability. Online community adults; associations involving overall flexibility and suppression were not evident among older adults.

Interpretation. A positive concurrent association is supported in this sample, with uncertain replication and population generalizability.

Sources. Ang and Tsai (2025). JBI S5; manuscript 3.4. Shared publication/reporting-bias limitations apply.

Appraisal of outcomes synthesized narratively

General well-being

Evidence. Age/gender-adjusted WHO-5 associations: overall flexibility $r = .122$, $p = .071$; suppression $r = .115$, $p = .089$; enhancement $r = .034$, $p = .615$.

Risk of bias. JBI supports stated measurement and adjustment; residual confounding and shared self-report method remain possible.

Consistency and precision. One study; small estimated coefficients do not establish equivalence or a precisely characterized population effect.

Applicability. Predominantly university-based young adults using the German FREE scale.

Interpretation. The positive overall-flexibility and suppression coefficients did not meet the conventional significance threshold; replication is needed to establish the magnitude and reliability of these associations.

Sources. Gonzalez-Escamilla et al. (2022). JBI S5; manuscript 3.4. Shared publication/reporting-bias limitations apply.

Psychological health-related quality of life

Evidence. Suppression showed favorable associations. Quattropani found an inverse adjusted enhancement association; Sardella found overall-flexibility associations at baseline and follow-up.

Risk of bias. Sardella has EF-measurement uncertainty and follow-up limitations; its baseline overall-flexibility association is an unadjusted correlation, and its follow-up model did not adjust for baseline HRQoL.

Consistency and precision. Two studies with different estimands; no pooled magnitude. Follow-up HRQoL is not equivalent to improvement in HRQoL.

Applicability. Online community adults versus geriatric outpatients; Quattropani assessed components only.

Interpretation. Evidence favors further study of suppression and overall flexibility, with component-specific and temporal qualifications.

Sources. Quattropani et al. (2022); Sardella et al. (2021). JBI S5; manuscript 3.4. Shared publication/reporting-bias limitations apply.

Hope

Evidence. Overall flexibility and both components were positively associated with trait hope; the suppression association was absent among older men.

Risk of bias. JBI supports measurement; the overall and component associations are unadjusted bivariate correlations, with age and gender examined as moderators. Concurrent self-reports cannot establish direction or rule out shared-method influences.

Consistency and precision. One study; no between-study consistency or pooled precision estimate.

Applicability. Online community adults with age/gender differences.

Interpretation. A concurrent relationship is suggested; enhancement of hope through EF training has not been tested.

Sources. Ang and Tsai (2025). JBI S5; manuscript 3.4. Shared publication/reporting-bias limitations apply.

Appraisal of outcomes synthesized narratively

Resilience

Evidence. Both studies report positive associations involving overall flexibility and its components, using CD-RISC and RS-11 respectively.

Risk of bias. Chen's resilience correlations have no confounding strategy; Gonzalez includes adjustment. Both use self-report measures.

Consistency and precision. Convergent directions across two studies, but different resilience instruments and no common pooled magnitude.

Applicability. Chinese undergraduates and predominantly university-based German young adults.

Interpretation. A concurrent association is supported; prospective adaptation after adversity cannot be inferred from resilience questionnaire scores alone.

Sources. Chen et al. (2018); Gonzalez-Escamilla et al. (2022). JBI S5; manuscript 3.4. Shared publication/reporting-bias limitations apply.

Ego-resilience

Evidence. Overall flexibility and both components were positively associated with ego-resilience.

Risk of bias. JBI supports outcome measurement but identifies no confounding strategy; exposure and outcome are concurrent self-reports.

Consistency and precision. One study; no independent replication or pooled precision assessment.

Applicability. Community adults recruited online during FREE development.

Interpretation. Initial construct-related evidence is available; temporal direction and broader generalizability remain unestablished.

Sources. Burton and Bonanno (2016), Study 1. JBI S5; manuscript 3.4. Shared publication/reporting-bias limitations apply.

Friend-rated adjustment under stress

Evidence. Overall flexibility was associated with better friend-rated adjustment under higher cumulative stress, particularly in the immediate-threat condition.

Risk of bias. JBI supports measurement and adjustment but flags incomplete eligibility/setting reporting. The reviewed association is concurrent at the follow-up assessment.

Consistency and precision. One sample of 50; conditional findings across task conditions are not independent replications. The cohort partly overlaps with Bonanno.

Applicability. Non-clinical undergraduates; friend ratings reduce same-reporter overlap but do not remove confounding.

Interpretation. Context-dependent adjustment associations merit replication; this result is not a second independent prospective EF effect.

Sources. Westphal et al. (2010); cohort overlap with Bonanno et al. (2004). JBI S5; manuscript 3.4. Shared publication/reporting-bias limitations apply.

Appraisal of outcomes synthesized narratively

Burnout dimensions

Evidence. Overall flexibility was inversely associated with emotional exhaustion after adjustment; no associations were observed for depersonalization or personal accomplishment.

Risk of bias. JBI identifies uncertainty in condition ascertainment, EF measurement and statistical inference, including team dependence.

Consistency and precision. One study of 65 practitioners; no common effect across the three dimensions and no replication.

Applicability. Palliative home-care practitioners; a specific occupational setting.

Interpretation. The signal concerns emotional exhaustion only. Evidence does not establish a broad burnout association or prevention effect.

Sources. Lenzo et al. (2020). JBI S5; manuscript 3.4. Shared publication/reporting-bias limitations apply.

Physical health-related quality of life

Evidence. Quattropani found no adjusted component associations. Sardella found favorable suppression associations at baseline and follow-up.

Risk of bias. Sardella has EF-measurement and follow-up limitations; baseline physical HRQoL was not controlled in the follow-up model.

Consistency and precision. Findings differ across samples and estimands; no pooled magnitude or evidence of change attributable to suppression.

Applicability. Community adults versus geriatric outpatients; SF-12 PCS in both studies.

Interpretation. A suppression-specific association in older outpatients warrants replication; a general overall-flexibility association is not established.

Sources. Quattropani et al. (2022); Sardella et al. (2021). JBI S5; manuscript 3.4. Shared publication/reporting-bias limitations apply.

Perceived social support

Evidence. Overall flexibility was positively associated with support in both studies; some component and age/gender patterns differed.

Risk of bias. Shangguan's reviewed support association lacks confounding control. Concurrent self-reports in both studies leave shared-method and reverse-direction explanations.

Consistency and precision. Two studies; the older-men subgroup differs in Ang. Cross-sectional mediation does not establish the mechanism or temporal order.

Applicability. Chinese college students and online community adults; different support measures.

Interpretation. A positive concurrent relationship has some replication, but universality and a causal support pathway are unestablished.

Sources. Shangguan et al. (2022); Ang and Tsai (2025). JBI S5; manuscript 3.4. Shared publication/reporting-bias limitations apply.

Appraisal of outcomes synthesized narratively

Social functioning deficits

Evidence. Higher overall flexibility and both components were associated with fewer LFQ social-functioning deficits.

Risk of bias. JBI supports measurement but identifies no confounding strategy; concurrent self-reports.

Consistency and precision. One study; no independent replication or pooled precision assessment.

Applicability. Online community adults; perceived functioning rather than observed interaction performance.

Interpretation. Initial evidence links perceived expressive ability with perceived functioning; causal and behavioral implications require testing.

Sources. Burton and Bonanno (2016), Study 1. JBI S5; manuscript 3.4. Shared publication/reporting-bias limitations apply.

Peer acceptance

Evidence. In 147 adolescents, targets displaying both expressive abilities received higher immediate liking ratings than targets displaying only one ability.

Risk of bias. JBI flags reporting gaps in randomization, concealment, baseline comparability, blinding and excluded-participant handling; these are uncertainties, not demonstrated failures.

Consistency and precision. One experiment; no independent replication or pooled effect estimate.

Applicability. Standardized videos and immediate liking of another person; not training of the participant's own flexibility or a clinical outcome.

Interpretation. Experimental support concerns this specific peer-perception contrast. Broader social or therapeutic benefits remain unestablished.

Sources. Wang et al. (2020), Experiment 1. JBI S5; manuscript 3.4. Shared publication/reporting-bias limitations apply.

Friendship quality

Evidence. Concurrent associations were positive. Unconditional prospective paths were not supported; conditional paths varied with social anxiety, with an adverse suppression path at extreme anxiety.

Risk of bias. JBI flags school clustering and missing-follow-up handling; panel adjustment does not establish within-person causality.

Consistency and precision. One three-wave study; multiple paths are not independent replications and conditional results require confirmation.

Applicability. Chinese adolescents, CAFE and self-reported friendship quality.

Interpretation. Concurrent and conditional prospective evidence is available; a general beneficial prospective effect is not established.

Sources. Wang et al. (2023). JBI S5; manuscript 3.4. Shared publication/reporting-bias limitations apply.

Relationship stress

Evidence. Overall flexibility and both components showed inverse bivariate associations. Adjusted pleasant- and unpleasant-expression ability associations had opposite signs.

Risk of bias. JBI flags EF scoring/measurement uncertainty; adjusted valence-specific findings must not be assigned to unadjusted overall-flexibility correlations.

Consistency and precision. One study; distinct scoring approaches and conditional models do not estimate a single common effect.

Applicability. Chinese college students; relationship-stress subscale and self-reported expressive ability.

Interpretation. An inverse concurrent overall-score association is suggested, but expression valence matters and causal benefits are unestablished.

Sources. Zhao and Wang (2024). JBI S5; manuscript 3.4. Shared publication/reporting-bias limitations apply.
